# Synthesis of Aminoethyl‐Substituted Piperidine Derivatives as σ_1_ Receptor Ligands with Antiproliferative Properties

**DOI:** 10.1002/cmdc.202100735

**Published:** 2022-02-09

**Authors:** Catharina Holtschulte, Frederik Börgel, Stefanie Westphälinger, Dirk Schepmann, Gianluca Civenni, Erik Laurini, Domenico Marson, Carlo V. Catapano, Sabrina Pricl, Bernhard Wünsch

**Affiliations:** ^1^ Institut für Pharmazeutische und Medizinische Chemie Westfälische Wilhelms-Universität Münster Corrensstraße 48 48149 Münster Germany; ^2^ Institute of Oncology Research Università della Svizzera Italiana (USI) Via Vincenzo Vela 6 6500 Bellinzona Switzerland; ^3^ Molecular Biology and Nanotechnology Laboratory (MolBNL@UniTS), DEA University of Trieste 34127 Trieste Italy; ^4^ Department of General Biophysics Faculty of Biology and Environmental Protection University of Lodz 90-237 Lodz Poland; ^5^ Chemical biology of ion channels (Chembion) Westfälische Wilhelms-Universität Münster Corrensstraße 48 48149 Münster Germany

**Keywords:** σ_1_ receptor ligands, piperidines, synthesis, σ_1_ affinity, selectivity over σ_2_ receptors, structure affinity relationships, molecular dynamics simulations, logD_7.4_ values, lipophilic ligand efficiency, cytotoxic activity, antitumor activity, human non-small cell lung cancer cells A427, androgen negative human prostate cancer cells DU145.

## Abstract

A series of novel σ_1_ receptor ligands with a 4‐(2‐aminoethyl)piperidine scaffold was prepared and biologically evaluated. The underlying concept of our project was the improvement of the lipophilic ligand efficiency of previously synthesized potent σ_1_ ligands. The key steps of the synthesis comprise the conjugate addition of phenylboronic acid at dihydropyridin‐4(1*H*)‐ones **7**, homologation of the ketones **8** and introduction of diverse amino moieties and piperidine N‐substituents. 1‐Methylpiperidines showed particular high σ_1_ receptor affinity and selectivity over the σ_2_ subtype, whilst piperidines with a proton, a tosyl moiety or an ethyl moiety exhibited considerably lower σ_1_ affinity. Molecular dynamics simulations with per‐residue binding free energy deconvolution demonstrated that different interactions of the basic piperidine‐N‐atom and its substituents (or the cyclohexane ring) with the lipophilic binding pocket consisting of Leu105, Thr181, Leu182, Ala185, Leu186, Thr202 and Tyr206 are responsible for the different σ_1_ receptor affinities. Recorded logD_7.4_ and calculated clogP values of **4a** and **18a** indicate low lipophilicity and thus high lipophilic ligand efficiency. Piperidine **4a** inhibited the growth of human non‐small cell lung cancer cells A427 to a similar extent as the σ_1_ antagonist haloperidol. 1‐Methylpiperidines **20a**, **21a** and **22a** showed stronger antiproliferative effects on androgen negative human prostate cancer cells DU145 than the σ_1_ ligands NE100 and S1RA.

## Graphical abstract

**Seven to eight** reaction steps led to novel σ_1_ receptor ligands with a 4‐(2‐aminoethyl)‐2‐phenylpiperidine scaffold. 1‐Methylpiperidine derivatives show high σ_1_ affinity, considerable selectivity over the σ_2_ subtype, and an improved Lipophilicity‐corrected Ligand Efficiency (LELP) index. Molecular dynamics simulations with per‐residue binding free energy deconvolution demonstrated that different interactions of the basic piperidine N atom and its substituents with the lipophilic binding pocket are responsible for the different σ_1_ receptor affinities. 1‐Methylpiperidine derivatives show antiproliferative activity against the human prostate cancer cell line DU145.

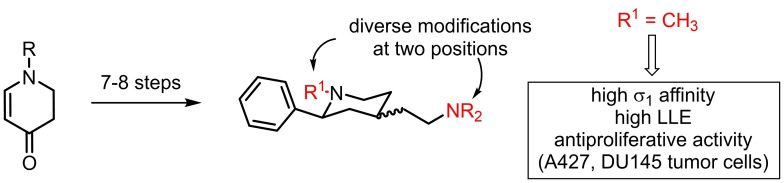

## Introduction

The σ_1_ and σ_2_ receptor subtypes differ in their molecular weight, tissue distribution and ligand binding profile.[^1^, ^2^, ^3^, ^4^] Both σ_1_ and σ_2_ receptors are expressed in fast proliferating cells such as prostate cancer, breast carcinoma or leukemia cells.^[5]^ Since this project was focused on σ_1_ receptor ligands, only the σ_1_ receptor subtype will be discussed further.

Twenty years after the first postulation of σ receptors by Martin et al.,^[1]^ the σ_1_ receptor was cloned from various tissues including liver (guinea pig), brain (rat, mouse), kidney (rat) and chorioncarcinoma cells (human).[^6^, ^7^, ^8^, ^9^, ^10^] The membrane bound protein consists of 223 amino acids resulting in a molecular weight of 25.3 kDa. The σ_1_ receptor proteins of different species have a high level of sequence identity (>93 % identity) yet they do not show any similarity to any other mammalian protein. Interestingly, a similarity of 65 % with the yeast sterol‐Δ^8^/Δ^7^‐isomerase has been detected although the σ_1_ receptor is devoid of sterol isomerase activity. On the other hand, some sterol‐Δ^8^/Δ^7^‐isomerase inhibitors bind with high affinity at the σ_1_ receptor.

In 2016, Kruse and coworkers produced the crystal structure of the σ_1_ receptor, revealing a trimeric form of the receptor protein.^[11]^ The N‐terminus consists of the unique transmembrane domain and a short extracellular peptide sequence. The C‐terminus is located on the cytosolic side of the membrane and forms a β‐barrel, which contains the ligand binding site. Intriguingly, the X‐ray‐determined protein structure differs considerably from that originally derived on the base on homology modeling techniques, nuclear magnetic resonance experiments and molecular biological investigations, all of which supported the existence of two transmembrane domains with both the C‐ and N‐terminal ends located on the cytosolic side.[^12^, ^13^] Two years later, the same group reported the structure of the σ_1_ receptor in complex with the prototypical agonist (+)‐pentazocine and the prototypical antagonist haloperidol.^[14]^

The σ_1_ receptor is not only expressed in the central nervous system (CNS), but also in some peripheral tissues including liver, heart and kidney.^[4]^ On the intracellular level, the σ_1_ receptor is predominantly found at the mitochondria‐associated membranes and at the endoplasmic reticulum (ER).[^15^, ^16^] It plays a key role in the regulation of ion channels (K^+^, Na^+^, Cl^−^ channels), the release and reuptake of neurotransmitters and the intracellular signaling through modulation of Ca^2+^ levels. As a chaperone in the ER, the σ_1_ receptor influences the activity of IP_3_ receptors and the transfer of Ca^2+^‐ions from the ER to mitochondria.[^17^, ^18^, ^19^] Pharmacologically, the σ_1_ receptor is involved in several neurological disorders including depression, alcohol and drug dependence, Parkinson's, Alzheimer‘s, Huntington's disease and neuropathic pain.[^20^, ^21^, ^22^, ^23^, ^24^]

I In addition to its high concentration in the CNS, the expression level of σ_1_ receptor in various human tumor cells is significantly increased compared to non‐tumor cells. This overexpression makes the σ_1_ receptor an attractive target for the development of novel antitumor strategies. Specifically, the σ_1_ receptor appears to be involved in programmed cell death (apoptosis). An increased σ_1_ receptor expression was associated with a poor clinical outcome and high risk of metastasis. Antiproliferative effects were observed after treatment of human tumor cells with various σ_1_ receptor antagonists. Moreover, the high density of σ_1_ receptors in tumor tissue can be exploited for the development of novel diagnostic tools to image tumor cells, to evaluate the treatment with anticancer drugs and to increase the understanding of tumor physiology and pathophysiology.[^5^, ^25^, ^26^, ^27^, ^28^, ^29^, ^30^] Several human tumor cells express an even higher amount of σ_2_ receptors, which represents the rationale to develop σ_2_ receptor‐based anticancer drugs and imaging tools.[^31^, ^32^, ^33^]

**Figure 1:**
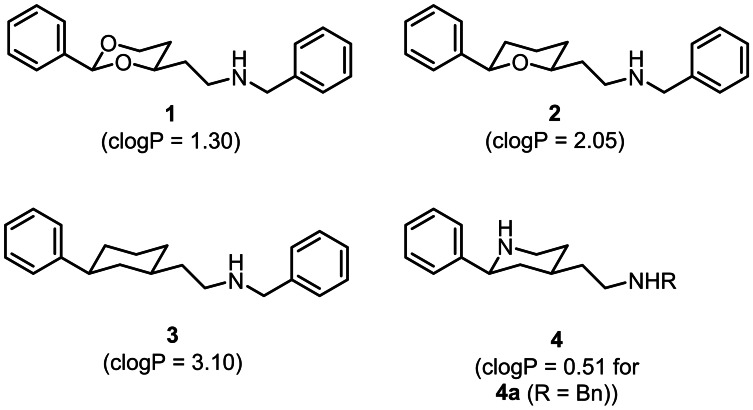
Reported σ_1_ receptor ligands **1**–**3** with 1,3‐dioxane (**1**), tetrahydropyran (**2**) and cyclohexane (**3**) scaffold in comparison with the designed piperidine‐based ligands **4** reported in this manuscript. clogP values were calculated using ChemAxon.

In literature, a large number of structurally diverse ligands interacting with the σ_1_ receptor is reported.^[24.28]^ Recently, the aminoethyl substituted 1,3‐dioxane **1** revealing low nanomolar σ_1_ affinity (*K*_i_=19 nM) and high antiallodynic activity *in vivo* (mouse capsaicin assay), which indicates σ_1_ antagonistic activity, was reported.[^34^, ^35^] (Figure 1) With respect to σ_1_ affinity, the enantiomer (2*S*,4*R*)‐**1** represents the eutomer (*K*_i_=6.0 nM).^[36]^ However, the acid‐labile acetalic substructure of **1** limits its further development. Therefore, ligands with a tetrahydropyran ring containing only one O‐atom ((2*R*,6*S*)‐**2**, *K*_i_=5.4 nM, (2*S*,6*R*)‐**2**, *K*_i_=1.6 nM)^[37]^ and ligands with a cyclohexane ring without O‐atom ((1*R*,3*S*)‐**3**, K_i_=0.61 nM, (1*S*,3*R*)‐**3**, K_i_=1.3 nM, see Table 1),^[38]^ which could not undergo further hydrolysis, were designed, synthesized and pharmacologically evaluated. Both tetrahydropyrans **2** and cyclohexanes **3** exhibit high σ_1_ affinity, selectivity over the σ_2_ subtype and, importantly, growth inhibition of the androgen negative human prostate cancer cell line DU145.[^37^, ^38^]

However, the penalty for increased hydrolytic stability and σ_1_ receptor affinity of tetrahydropyrans **2** and cyclohexanes **3** is an increased molecular lipophilicity. In Figure 1 the calculated clogP values of the σ_1_ ligands **1**–**3** and one designed piperidine **4 a** are summarized. In order to confirm the predicted clogP values, the logD_7.4_ values of the σ_1_ ligands **1**–**4 a** were also determined experimentally using the micro shake flask method.[^39^, ^40^]

In an effort to maintain high σ_1_ affinity of the lead compounds **1**–**3** and, simultaneously, increase hydrolytic stability and reduce lipophilicity, the central 1,3‐dioxane, tetrahydropyran or cyclohexane ring of the lead compounds **1**–**3** was replaced by a piperidine ring (**4**, Figure 1). The calculated clogP value of −0.51 for the designed piperidine **4a** is rather low, which is due to the additional secondary amine in the piperidine ring. Furthermore, the additional N‐atom in the piperidine ring entails the possibility to introduce further and diverse substituents at this position and this, in turn, allows for the modulation of σ_1_ affinity, selectivity over the σ_2_ subtype, lipophilicity, polarity and finally pharmacokinetic properties of the ligands **4**.

Herein, we present the synthesis, receptor affinity and structure activity relationships of novel piperidine derivatives of type **4**. Moreover, the effects on tumor cell growth will be reported.

## Results and Discussion

### Synthesis

**Scheme 1:**
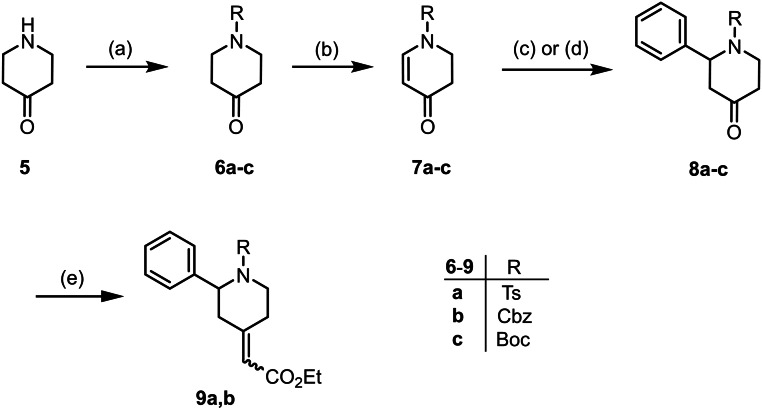
Synthesis of α,β‐unsaturated esters **9a,b**: (a) pTsCl, K_2_CO_3_, CH_3_CN, 18 h, rt, **6a**, 95 %. (b) IBX, NMO, DMSO, 3 d, 30 °C, **7a**, 77 %, **7b**, 83 %, **7c**, 77 %. (c) Phenylboronic acid, [Rh(cod)_2_]BF_4_, dioxane/H_2_O, **8a**, 34 %. (d) Phenylboronic acid, [Rh(cod)_2_]BF_4_, dioxane/KOH, **8b**, 71 %. (e) Ph_3_P=CHCO_2_Et, toluene, 18 h, reflux; **9a**, 103 % (contains small amounts of Ph_3_PO); **9b**, 98 %.

**Scheme 2:**
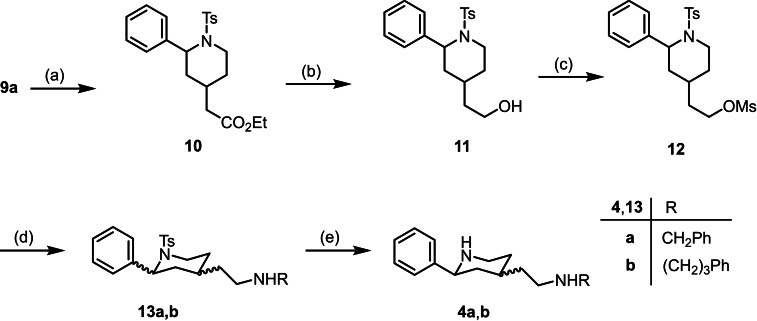
Synthesis of σ receptor ligands **4** from tosyl derivative **9a**: (a) H_2_ (balloon), Pd/C (10 %), CH_3_OH, 20 h, rt, 81 %. (b) LiAlH_4_, THF, 2.5 h, rt, 89 %. (c) CH_3_SO_2_Cl, Et_3_N, CH_2_Cl_2_, 18 h, rt, 94 %. (d) Benzylamine or 3‐phenylpropan‐1‐amine, CH_3_CN, 18 h, reflux, 60 % (**13a**), 87 % (**13b**). (e) Mg^0^ turnings, CH_3_OH, ultrasonic irradiation, 5 h, rt, 80 % (**4a**), 37 % (**4b**).

**Scheme 3:**
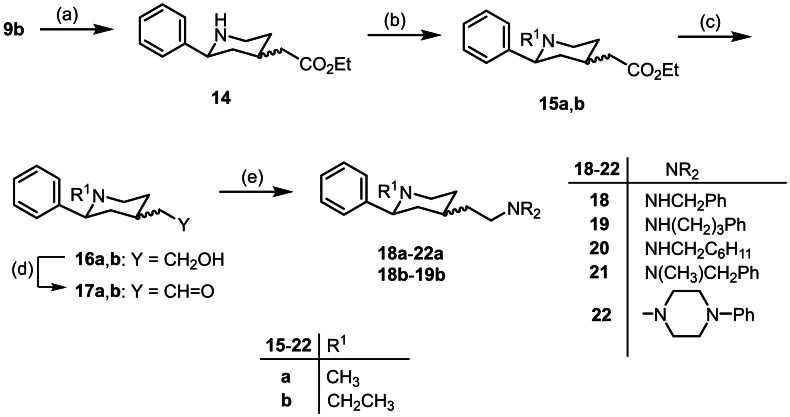
Synthesis of σ receptor ligands from Cbz derivative **9b**: (a) H_2_ (3 bar), Pd/C (10 %), CH_3_OH, 20 h, rt, 70 %. (b) formalin or CH_3_CH=O, NaBH(OAc)_3_, CH_2_Cl_2_, 18 h, rt, 66 % (**15a**), 68 % (**15b**) (c) LiAlH_4_, THF, 2 h, rt, 85 % (**16a**), 85 % (**16b**). (d) DMP, CH_2_Cl_2_, 2 h, rt, 62 % (**17a**), 93 % (**17b**). (e) R_2_NH, NaBH(OAc)_3_, CH_2_Cl_2_, 3 h, rt, 11–69 %.

Piperidines of type **4** were obtained by conjugate addition of a phenyl nucleophile at the α,β‐unsaturated ketones **7** and subsequent introduction of a C_2_ chain by a Wittig reaction (Scheme 1). Transformation of the ester group into an amino moiety and removal of the N‐protective group represent the final steps of the synthesis (Schemes 2 and 3).

Whereas Cbz‐ and Boc‐protected piperidin‐4‐ones **6b** and **6c** were commercially available, the tosyl‐protected piperidin‐4‐one **6a** was prepared by tosylation of piperidin‐4‐one (**5**). Oxidation of piperidin‐4‐ones **6a**‐**c** with iodoxybenzoic acid (IBX)^[41]^ provided the α,β‐unsaturated ketones (vinylogous amides) **7a**–**c** in 77–83 % yield. Addition of *N*‐methylmorpholin‐*N*‐oxide (NMO) allowed conducting the oxidation under very mild reaction conditions (30 °C), which gave high yields (Scheme 1).

The conjugate addition of a phenyl nucleophile at the α,β‐unsaturated ketones **7 a**–**c** served as the key step in the synthesis of the designed ligands. The Rh‐catalyzed ([Rh(cod)_2_]BF_4_) conjugate addition of phenylboronic acid^[42]^ at the tosyl‐protected dihydropyridin‐4‐one **7a** in a dioxane/KOH mixture led to decomposition of **7a**. To prevent decomposition of **7a** the reaction was performed in a dioxane/water mixture without addition of a base. After optimization of the reaction conditions, the addition product **8a** was isolated in 34 % yield. The Cbz‐protected dihydropyridine **7b** turned out to be more stable and tolerated well the conjugate addition in dioxane/KOH resulting in 71 % yield of the addition product **8b**. Despite thorough modification of the reaction conditions, the Rh‐catalyzed conjugated addition of phenylboronic acid at the Boc‐protected dihydropyridine **7c** did not lead to the addition product **8c** (Scheme 1).

Next, the ketones **8a** and **8b** were expanded by a two‐carbon chain. To this purpose, the ketones **8a** and **8b** were reacted with the stabilized P‐ylide Ph_3_P=CHCO_2_Et to form α,β‐unsaturated esters **9a** and **9b**. The α,β‐unsaturated esters **9a** and **9b** were obtained as mixtures of (*E*)‐ and (*Z*)‐diastereomers. The ratio was 60 : 40 for the tosyl derivatives (*Z*)‐**9a** : (*E*)‐**9a** and 55 : 45 for the Cbz derivatives (*Z*)‐**9b** : ( *E*)‐**9b** (Scheme 1).

The α,β‐unsaturated ester **9a** with a tosyl protective group was hydrogenated using the catalyst Pd/C. The saturated ester was isolated as a mixture of *cis*‐**10** : *trans*‐**10** (35 : 65). LiAlH_4_ reduction of the ester **10** led to the primary alcohol **11** (*cis*‐**11** : *trans*‐**11**=83 : 17), which was reacted with methanesulfonyl chloride to afford the mesylate **12** (*cis*‐**12** : *trans*‐**12**=83 : 17). Nucleophilic substitution of the mesylate **12** with benzylamine or 3‐phenylpropan‐1‐amine led to the secondary amines **13a** and **13b** in 60 % and 87 % yield, respectively. Both amines were isolated as 65 : 35‐mixture of *cis*‐ and *trans*‐configured diastereomers. Finally, the tosyl moiety of **1 a** and **1 b** was removed with Mg^0^ in methanol to provide the diamines **4a** and **4b**. The benzylamine **4a** was isolated in 80 % yield (*cis*‐**4a** : *trans*‐**4a**=75 : 25) and the phenylpropylamine **4b** in 37 % yield (*cis*‐**4b** : *trans*‐**4b**=65 : 35) (Scheme 2).

The structure of the signal for the axially oriented proton in 3‐position of the main diastereomer unequivocally proves its *cis*‐configuration. As an example, a dt (*J*=13.5/10.3 Hz) at 1.54 ppm and a broad q *(J*=12.1 Hz) at 1.20 ppm are observed for 3‐H_ax_ of **13a** and **4b**, respectively. The large coupling constants originate from germinal coupling with 3‐H_eq_ and vicinal couplings with two axially oriented protons in 2‐ and 4‐pposition indicating the equatorial orientation of both substituents at 2‐ and 4‐position at the piperidine ring. Since the signal structures for 2‐H_ax_ (dd, J=9.7–11.3 Hz and 2.4‐2.9 Hz) of both diastereomers of **4a** and **4b** are identical, the phenyl ring of both diastereomers adopts the equatorial orientation. Thus, *cis‐* and *trans*‐configured diastereomers differ in the orientation of the aminoethyl moiety at the 4‐position.

During hydrogenation of the α,β‐unsaturated ester **9b**, hydrogenolytic cleavage of the Cbz moiety at the piperidine ring occurred as well. The saturated ester **14** was isolated in 70 % yield as mixture of *cis*‐ and *trans*‐diastereomers (ratio 75 : 25). The secondary amine **14** was reductively alkylated with formalin or acetaldehyde using NaBH(OAc)^[43]^ as reducing agent to afford the methyl and ethyl derivatives **15a** and **15b**, respectively. LiAlH_4_ reduction of the esters **15a** and **15b** provided the primary alcohols **16a** and **16b**. Activation of the primary alcohol **16a** with methanesulfonyl chloride as shown for the alcohol **11** led to a mesylate, which reacted directly with the tertiary amino moiety of the piperidine ring to form a 1‐azoniabicyclo[2.2.2]octane derivative. Therefore, the alcohols **16a** and **16b** were oxidized with Dess‐Martin‐Periodinane (DMP)^[44]^ to give the aldehydes **17a** and **17b**, which were reductively aminated with various primary and secondary amines and NaBH(OAc)_3_ ^[43]^ to provide the secondary and tertiary amines **18**–**22** (Scheme 3). The final amines **18**–**22** were isolated as mixtures of diastereomers (*cis* : *trans*=60 : 40 to 85 : 15). The quartet‐like structure or the dt structure (J >11 Hz, respectively) of the signal for the axially oriented proton in 3‐postion confirms the *cis*‐configuration of the main diastereomer.

Since for tetrahydropyrans and cyclohexanes the σ_1_ affinities of *cis*‐ and *trans*‐configured diastereomers were very similar[^37^, ^38^] and, moreover, the separation of *cis*‐ and *trans*‐configured piperidines turned out to be very difficult, mixtures of diastereomers **4**, **13** and **18**–**22** were tested, respectively.

### σ_1_ and σ_2_ receptor affinity

The affinity of the synthesized piperidines towards σ_1_ and σ_2_ receptors was determined in radioligand receptor binding assays. In the σ_1_ assay, homogenates of guinea pig brains were used as receptor material and [^3^H]‐(+)‐pentazocine as σ_1_ selective radioligand. The receptor material in the σ_2_ assay was a membrane preparation from rat liver. As a σ_2_ selective radioligand is not available, the assay was performed with the non‐selective radioligand [^3^H]‐1,3‐di(o‐tolyl)guanidine ([^3^H]DTG). In order to occupy σ_1_ receptors and render the assay selective for the σ_2_ subtype, an excess of non‐tritiated (+)‐pentazocine was added.[^45^, ^46^, ^47^] Affinity data obtained with receptor preparations containing guinea pig and human σ_1_ receptors are well comparable, since the amino acid sequences of guinea pig and human σ_1_ receptors are 93 % identical.^[48]^ Furthermore, binding studies with rat and human σ_2_ receptors result in comparable affinity data for potent and selective σ_2_ ligands.[^49^, ^50^] In Table 1, the σ affinity of the synthesized compounds is compared with the σ affinity of some lead and reference compounds.

**Table 1:** σ_1_ and σ_2_ receptor affinity of synthesized piperidines and some lead and reference compounds.

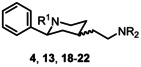					
Compd	R^1^	NR_2_	*K*_i_±SEM [nM] (*n*=3)		σ_1_ : σ_2_
			σ_1_	σ_2_	selectivity
**1^36^**	1,3‐dioxane^[a]^	NHCH_2_Ph	6.0±1.0	4200	14
**2^37^**	tetrahydropyran^[a]^	NHCH_2_Ph	1.6±0.2	378	236
**3^38^**	cyclohexane^[a]^	NHCH_2_Ph	0.61±0.1	49±31	80
**4a**	H	NHCH_2_Ph	165	372	2.3
**4b**	H	NH(CH_2_)_3_Ph	849	0 %^[b]^	–
**13a**	Ts	NHCH_2_Ph	57±21	763	13
**13b**	Ts	NH(CH_2_)_3_Ph	291±139	567	2
**18a**	CH_3_	NHCH_2_Ph	7.9±0.2	483	61
**18b**	CH_2_CH_3_	NHCH_2_Ph	129±38	131	1
**19a**	CH_3_	NH(CH_2_)_3_Ph	50±17	0 %^[b]^	>20
**19b**	CH_2_CH_3_	NH(CH_2_)_3_Ph	2400	334	0,14
**20a**	CH_3_	NHCH_2_C_6_H_11_	16±5	285	18
**21a**	CH_3_	N(CH_3_)CH_2_Ph	19±9	77±5	4
**22a**	CH_3_	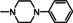	27±11	1600	59
(+)‐pentazocine		–	5.4±0.5	–	–
haloperidol		–	6.6±0.9	125±33	19
di‐*o*‐tolylguanidine		–	71±7.9	54±8	0.76

[a] Structures of compounds **1**–**3** are shown in Figure 1. [b] For compounds with low affinity the inhibition (in %) of radioligand binding at a test compound concentration of 1 μM is given. The piperidines were tested as mixtures of *cis*‐ and *trans*‐configured diastereomers. *cis* : *trans*=60 : 40 to 85 : 15.

Replacement of the central cyclohexane ring of the lead compound **3** (*K*_i_(σ_1_)=0.61 nM) by a piperidine ring without N‐substituent led to remarkably reduced σ_1_ affinity of the secondary amine **4 a** (*K*_i_(σ_1_)=165 nM). Introduction of an ethyl (**18b**) or tosyl moiety (**13a**) increased the σ_1_ affinity slightly, but a small methyl moiety at the piperidine N‐atom resulted in rather high σ_1_ affinity. The σ_1_ affinity of the piperidine **18a** (*K*_i_(σ_1_)=7.9 nM) is only 10‐fold lower than the σ_1_ affinity of the cyclohexane derivative **3** and equipotent with the 1,3‐dioxane derivative **1**. It has to be noted that **18a** was tested as mixture of diastereomers *cis*‐**18a** : *trans*‐**18a**=85 : 15.

Extension of the distance between the basic N‐atom and the terminal phenyl moiety from one methylene moiety (benzylamines) to three methylene moieties (3‐phenylpropylamines) led to reduced σ_1_ affinity of **4b**, **13b**, **19a** and **19b**. As observed for the benzylamine **18a**, the piperidine derivative **19a** with the small N‐methyl moiety showed the highest σ_1_ affinity (*K*_i_(σ_1_)=50 nM) of the series of 3‐phenylpropylamines. Therefore, further variations at the terminal N‐atom were performed starting with the piperidine ring bearing the small methyl moiety. Although the cyclohexylmethylamine **20a** and the tertiary amines **21a** and **22a** revealed slightly reduced σ_1_ affinity compared to the benzylamine **18a**, their *K*_i_ values are still in the low nanomolar range (*K*_i_(σ_1_) <27 nM).

The most potent ligands bearing a methyl moiety at the piperidine N‐atom reveal high selectivity for the σ_1_ over the σ_2_ receptor. In particular, the benzylamine **4a**, the cyclohexylmethylamine **20a** and the phenylpiperazine **22a** exhibit 60‐, 18‐ and 60‐fold σ_1_:σ_2_ selectivity, respectively. The lowest σ_1_:σ_2_ selectivity (4‐fold) was found for the N‐benzyl‐N‐methylamine **21a**.

In contrast, piperidine derivatives **18b** and **19b** bearing an ethyl moiety at the piperidine N‐atom display higher σ_2_ affinity. Whereas the benzylamine **18b** has the same affinity towards both σ_1_ and σ_2_ receptors, the homologous phenylpropylamine **19b** reveals a 7‐fold preference for the σ_2_ receptor over the σ_1_ receptor.

### Molecular dynamics simulation

**Figure 2:**
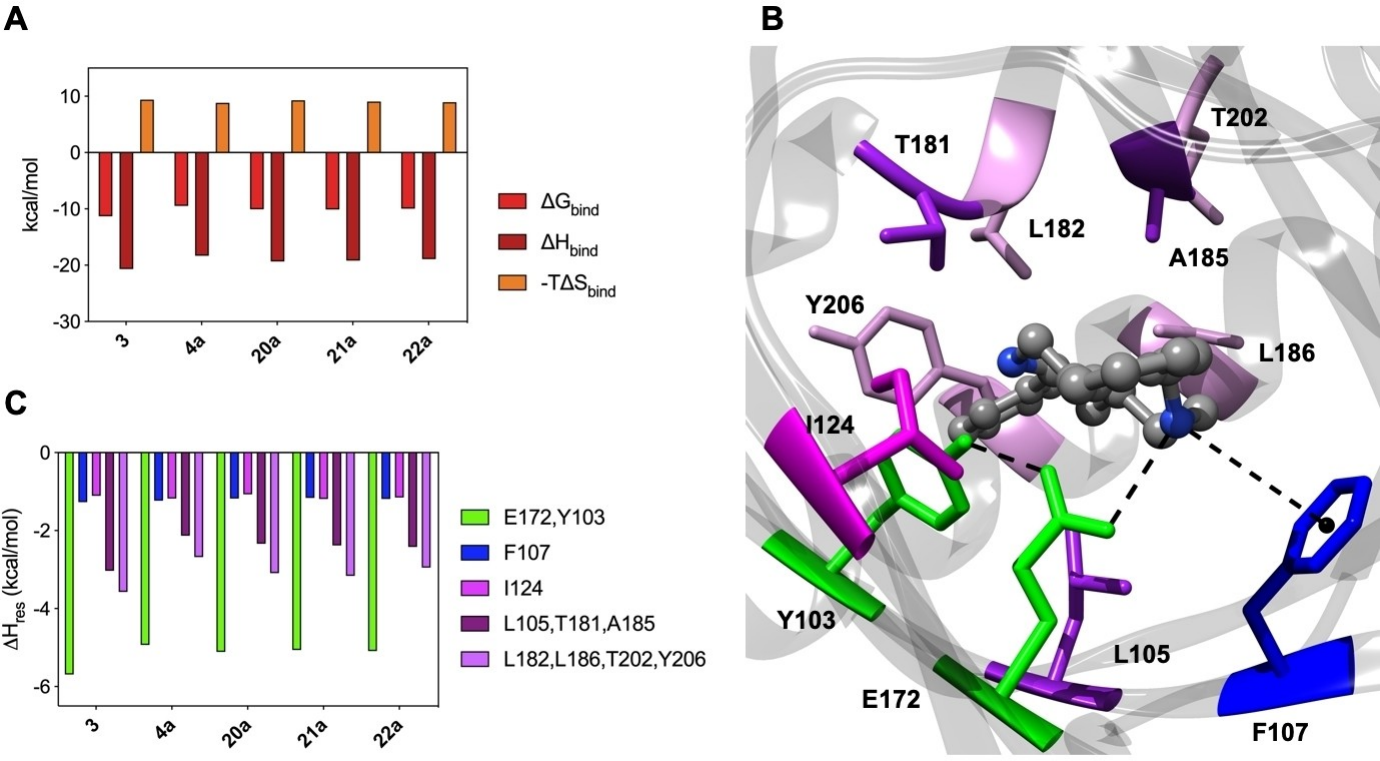
(A) Calculated free energy of binding (ΔG_bind_), and enthalpic (ΔH_bind_) and entropic (‐TΔS_bind_) components for the σ_1_ *receptor* complexed with **3**, **4a**, **20a**, **21a** and **22a** (B) Details of compound **4a** in the binding pocket of the σ_1_ receptor. **4a** is shown as atom‐colored sticks‐and‐balls (C, grey, N, blue, O, red) while the side chains of the protein residues mainly interacting with **4a** are depicted as colored sticks and labelled. Hydrogen atoms, water molecules, ions, and counterions are omitted for clarity. (C) Per‐residue binding free energy decomposition of the main involved amino acids of the complex between σ_1_ receptor and **3**, **4a**, **20a**, **21a** and **22a**.

Piperidine **4a** and the methylated derivatives **20a**, **21a**, and **22a** are provided with high σ_1_ affinity. Accordingly, we carried out Molecular Dynamics (MD) simulations to investigate the interactions of these compounds with the σ_1_ receptor. Initially, the putative binding modes were identified using a well‐validated docking protocol.[^37^, ^38^] Next, MD simulations of the resulting σ_1_ receptor/piperidine derivative complexes were carried out, and the corresponding ligand/protein free energy of binding (ΔG_bind_) values were obtained via the Molecular Mechanics/Poisson‐Boltzmann Surface Area (MM/PBSA) approach.^[51]^ According to the simulations, and in agreement with the corresponding experimental profiles, binding of the piperidine derivatives at σ_1_ receptor provided a lower Gibbs free energy of binding ΔG_bind_ than binding of the previously reported cyclohexane derivatives.^[37]^ In terms of enthalpic and entropic contributions, the piperidines exhibit a similar thermodynamics trend as cyclohexane **3** (Figure 2A), but their corresponding ΔG_bind_ values are more than 1 kcal/mol higher (Figure 2A, Table S1, ΔG_bind_(**3**)=−11.31 kcal/mol; ΔG_bind_(**4a**)=−9.48 kcal/mol; ΔG_bind_(**20a**)=−10.12 kcal/mol; ΔG_bind_(**21a**)=−10.06 kcal/mol; ΔG_bind_(**22a**)=−9.97 kcal/mol).

To explain the lower σ_1_ binding capability of the new piperidine derivatives, the individual intermolecular interactions were analyzed by performing a per‐residue binding free energy deconvolution (PRBFED) of the enthalpic terms ΔH_res_ (Figures 2B, 2C, Table S2). As expected, the **4a**/σ_1_ receptor complex revealed the prototypical pattern of intermolecular interactions underlying σ_1_ receptor ligand binding (Figure 2B). Specifically, the N‐atom of the basic benzylamino moiety of **4a** is engaged in two interactions in the σ_1_ binding site: i) a persistent salt bridge with the carboxylate moiety of Glu172, stabilized by an internal hydrogen bond with Tyr103 (ΣΔH_res_=−4.93 kcal/mol, Figure 2C and Table S2); and ii) a π‐cation interaction with the phenyl ring of Phe107 (ΔH_res_=−1.23 kcal/mol). Moreover, the side chain of Ile124 can support the appropriate orientation of the benzylamino moiety of **4a** in the receptor binding cavity with favorable hydrophobic interactions (ΔH_res_=−1.27 kcal/mol). On the other hand, the highly hydrophobic σ_1_ receptor binding site should assist nestling of the phenylpiperidine moiety of **4a**, but the presence of a further protonated amino moiety in this apolar region interferes with the lipophilic interactions with σ_1_ receptor residues (Figure 2B). Accordingly, a considerable decrease of the corresponding enthalpic contribution is detected by our PRBFED analysis compared to the cyclohexane derivative **3** (**4a**: ΣΔH_L105,T181,A185_=−2.23 kcal/mol, ΣΔH_L182,L186,T202,Y206_=−2.68 kcal/mol; **3**: ΣΔH_L105,T181,A185_=−3.03 kcal/mol, ΣΔH_L182,L186,T202,Y206_=−3.57 kcal/mol; Figure 2C and Table S2).

The N‐methylpiperidine derivatives **20a**, **21a**, and **22a** show very similar binding modes as **4a** and their interactions with σ_1_ receptor residues Tyr103, Phe107, Ile124 and Glu172 are practically unchanged (Figures 2C and S1). The presence of the small CH_3_ group on the N‐atom of the piperidine ring increases the lipophilic interactions with the σ_1_ receptor binding pocket compared with the secondary amine **4a**, but does not achieve the same value as the cyclohexane derivative **3**. Accordingly, the favorable enthalpic contribution provided by the interactions with the hydrophobic cavity of the σ_1_ receptor is significantly lower than the contribution of the analogous cyclohexane derivative **3** (**20a**: (ΣΔH_L105,T181,A185_=−2.34 kcal/mol, ΣΔH_L182,L186,T202,Y206_=−3.09 kcal/mol; **21a**: ΣΔH_L105,T181,A185_=−2.38 kcal/mol, ΣΔH_L182,L186,T202,Y206_=−3.16 kcal/mol; **22a**: ΣΔH_L105,T181,A185_=−2.42 kcal/mol, ΣΔH_L182,L186,T202,Y206_=−2.95 kcal/mol; Figure 2C and Table S2).

### Lipophilicity and lipophilic ligand efficiency

In order to argue with reliable lipophilicity values, the logD_7.4_ values of key compounds were determined experimentally following our micro‐shake‐flask protocol.[^39^, ^40^] According to this method, each compound of interest was distributed between an *n*‐octanol layer and an aqueous MOPS buffer pH 7.4. Subsequently, the amount of compound in the buffer layer was determined by mass spectrometry.

In Table 2, the experimentally determined logD_7.4_ values for lead compounds **1**–**3** and piperidines **4a** and **18a** are summarized. As expected, the most lipophilic compound is the cyclohexane derivative **3** with a logD_7.4_ value of 3.25. Introduction of one O‐atom into the cyclohexane ring (tetrahydropyran **2**) reduces the logD_7.4_ value by one order of magnitude. A second O‐atom as in 1,3‐dioxane **1** further reduces the lipophilicity by one order of magnitude. However, introduction of an NCH_3_ (**18a**) or NH (**4a**) moiety into the cyclohexane ring instead of one O‐atom resulted in very low logD_7.4_ values of 0.52 and −0.79.

**Table 2:** Experimentally determined logD_7.4_ values, calculated clogP values and lipophilic ligand efficiency indices, LLE and LELP.

no.	Compd	σ_1_ affinity *K*_i_ [nM]	logD_7.4_ (exp., *n*=3)	clogP^[a]^ (calcd.)	LLE^[b]^	LELP^[c]^
**1**	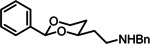	6.0	1.36±0.02	1.30	6.92	3.48
**2**	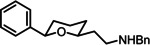	1.6	2.52±0.05	2.05	6.75	5.13
**3**	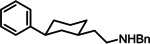	0.61	3.25±0.02	3.10	6.11	7.40
**4a**	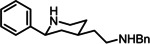	165	−0.79±0.07	−0.51	7.29	1.65^[d]^
**18a**	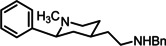	7.9	0.52±0.01	0.01	8.09	0.03

[a] clogP values were calculated with ChemAxon. [b] Lipophilic Ligand Efficiency (LLE) index is defined as: LLE=p*K*_i_−clogP. [c] Lipophilicity‐corrected Ligand Efficiency (LELP) index is defined as: LELP=clogP : LE; LE=p*K*_i_ : HAC (HAC: number of non‐H‐atoms of a drug). [d] For the calculation, the negative sign of the clogP value was ignored.

In addition, the corresponding clogP values for the same set of compounds were calculated by ChemAxon. As shown in Table 2, the calculated clogP values correlate well with the experimentally recorded logD_7.4_ values indicating that ChemAxon is a method leading to reliable predicted clogP values for this type of compounds.

Improving the potency of compounds is commonly achieved by increasing the molecular complexity in order to find the adequate interactions of the molecule with its target protein. However, addition of unnecessary molecular complexity often leads to “molecular obesity”.^[52]^ Obese molecules, *i. e*., rather complex molecules with high lipophilicity, often suffer from unfavorable pharmacokinetics (poor bioavailability) and non‐acceptable toxicological profile. Lipinski's “rule of five” is one of the earliest attempts to overcome the risk of obese drugs.[^52^, ^53^] In order to quickly analyze the impact of molecular complexity and lipophilicity for the quality of drugs at an early stage during the drug discovery process, several ligand efficiency indices have been defined and validated.[^54^, ^55^, ^56^] The Lipophilic Ligand Efficiency (LLE) index describes the contribution of the lipophilicity of a drug in form of the clogP value to its biological activity in form of *K*_i_, *K*_d_, or *IC*_50_ value (LLE=p*K*_i_ or p*K*_d_ or p*IC*_50_–clogP).^[57]^ Since the LLE index is not useful for very small and polar drugs, the Lipophilicity‐corrected Ligand Efficiency (LELP) index was defined taking the number of non‐H atoms (HAC) of a drug into account in addition to its clogP value (LELP=(clogP ⋅ HAC) : p*K*_i_).^[58]^ The LELP index describes the reduction of the drug efficiency of even very potent drugs by increasing their lipophilicity and size.^[59]^ Promising physicochemical properties are usually expected for drugs with a LLE index >5 and a LELP index <10.

With respect to efficiency, the benzylamines of all four compound classes fulfill the criteria of LLE>5 and LELP<10 (Table 2). However, the novel piperidines **4a** and **18a** show considerably higher LLE values than the corresponding 1,3‐dioxane **1**, tetrahydropyran **2** and cyclohexane **3**. Analogously, the LELP values of the piperidines **4a** and **18a** are very low, thereby rendering **4a** and **18a** particularly efficient drugs. The low σ_1_ affinity of **4a** (*K*_i_=165 nM) is compensated by its high polarity (low lipophilicity, logD_7.4_=−0.79).

### Growth inhibition of human tumor cell lines

In a preliminary experiment, the human non‐small cell lung cancer cell line A427^[60]^ was incubated with the low affinity σ_1_ ligand **4a** and the proliferation of the tumor cells was observed using the Live Cell Imager IncuCyte® allowing the continuous observation of the morphology, behavior and growth of the tumor cells. In this assay **4a** (*IC*_50_=17 μM) showed comparable growth inhibition as the prototypical σ_1_ antagonist haloperidol (*IC*_50_=16 μM, see Table S3 in Supporting Information). The effects of both compounds on A427 cells were considerably reduced in the presence of the prototypical σ_1_ agonist (+)‐pentazocine (10 μM) indicating a contribution of σ_1_ receptors to this effect (Table S3 in Supporting Information). Moreover, **4a** behaved as σ_1_ receptor antagonist in this A427 tumor cell proliferation assay.

Stimulated by the promising antiproliferative effect of **4a** on human non‐small cell lung cancer cells A427, the growth inhibition of the androgen negative human prostate cancer cells DU145^[61]^ was investigated. For this purpose, the methylated piperidines **20a**, **21a** and **22a** were selected, due to their promising σ_1_ affinity. In the assay, DU145 tumor cells were incubated in 96‐well plates for 24 h. Different concentrations of the test compounds were added and after incubation for additional 72 h, the amount of living cells was recorded by staining with Sulforhodamine B.^[62]^ In Table 3 the activity of the prototypical σ_1_ antagonists NE‐100 and S1RA is included. Figure S1 in the Supporting Information displays the corresponding graphics.

**Table 3:** Growth inhibition of androgen negative human prostate cancer cells DU145 by potent σ_1_ ligands.

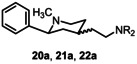			
Compd	NR_2_	σ_1_ affinity *K*_i_±SEM [nM]	cytotoxicity (DU145) *IC*_50_ [μM]
**20a**	NHCH_2_C_6_H_11_	16±5	4.9
**21a**	N(CH_3_)CH_2_Ph	19±9	5.5
**22a**	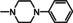	27±11	4.0
NE‐100		1.3^[63]^	>10
S1RA		17±7.0^[64]^	>10

σ_1_ affinity and cytotoxicity represent the data of three experiments (*n*=3).

The methylated piperidines **20a**, **21a**, and **22a** inhibit the growth of DU145 tumor cells with *IC_50_* values in the low micromolar range. Both the σ_1_ affinity and the antitumor activity of the three compounds are very similar. In this assay, the piperidines **20a**, **21a**, and **22a** are more potent than the reference σ_1_ antagonists NE‐100 and S1RA.

## Conclusion

Saturated six‐membered rings bearing an aminoethyl side chain show high σ_1_ receptor affinity and high selectivity over the σ_2_ subtype. However, 1,3‐dioxane **1** containing an acetal is not stable under acidic conditions (*e. g*., stomach) and the cyclohexane derivative **3** is rather lipophilic. Therefore, piperidines of type **4** were designed, which are hydrolytically stable and rather polar.

Piperidines **4** and **18**–**22** were prepared in a nine‐step synthesis. Piperidines with a methyl moiety at the piperidine N‐atom show high σ_1_ receptor affinity and σ_1_:σ_2_ selectivity indicating that it is possible to replace bioisosterically the 1,3‐dioxane ring of **1** or the cyclohexane ring of **3** by the piperidine ring with only slightly reduced σ_1_ affinity.

In addition to the high σ_1_ affinity, the piperidines **4a** and **18a** are polar compounds with very low experimentally determined logD_7.4_ values. As a result, the lipophilic ligand efficiency (LLE) index of the piperidines is considerably higher than the LLE of the lead compounds **1**–**3** even for **4a** exhibiting only low σ_1_ affinity (*K*_i_=165 nM). In case of **4a**, the low σ_1_ affinity is compensated by the low lipophilicity.

Molecular dynamics simulations and analysis of the per‐residue binding free energy revealed that the very polar protonated piperidine ring of **4a** reduces crucial lipophilic interactions within the lipophilic binding pocket of the σ_1_ receptor. Introduction of a NCH_3_ moiety (compounds **20a**, **21a**, **22a**) compensates partially these unfavorable interactions. However, the σ_1_ receptor affinity of the very lipophilic cyclohexane derivative **3** could not be achieved.

Due to their promising physicochemical properties, the inhibition of tumor cell growth by selected piperidines was investigated. The piperidine **4a** reduced the proliferation of non‐small cell lung cancer A427 cells similar to the σ_1_ antagonist haloperidol and the σ_1_ agonist (+)‐pentazocine abolished its effect. The methylated piperidines **20a**, **21a** and **22a** inhibited the growth of the androgen negative human prostate cancer cell line DU145. The piperidines are more active than the prototypical σ_1_ antagonists NE100 and S1RA, which underlines the favorable physicochemical properties of the piperidine‐based σ_1_ ligands.

## Experimental Section

### Chemistry, general

Unless otherwise noted, moisture sensitive reactions were conducted under dry nitrogen. CH_2_Cl_2_ was distilled over CaH_2_. THF was distilled over sodium/benzophenone. Et_2_O and toluene were dried over molecular sieves 4. Thin layer chromatography (tlc): Silica gel 60 F254 plates (Merck). Flash chromatography (fc): Silica gel 60, 40–64 μm (Merck); parentheses include: diameter of the column (d), length of the stationary phase (l), fraction size (V), eluent. Melting point: Melting point apparatus Mettler Toledo MP50 Melting Point System, uncorrected. MS: microTOF−Q II (Bruker Daltonics); APCI, atmospheric pressure chemical ionization. IR: FT‐IR spectrophotometer MIRacle 10 (Shimadzu) equipped with ATR technique. Nuclear magnetic resonance (NMR) spectra were recorded on Agilent 600‐MR (600 MHz for ^1^H, 151 MHz for ^13^C) or Agilent 400‐MR spectrometer (400 MHz for ^1^H, 101 MHz for ^13^C); *δ* in ppm related to tetramethylsilane and measured referring to CHCl_3_ (*δ*=7.26 ppm (^1^H NMR) and *δ*=77.2 ppm (^13^C NMR)), CHD_2_OD (*δ*=3.31 ppm (^1^H NMR) and *δ*=49.0 ppm (^13^C NMR)) and DMSO‐*d_6_* (*δ*=2.54 ppm (^1^H NMR) and *δ*=39.5 ppm (^13^C NMR)); coupling constants are given with 0.5 Hz resolution; the assignments of ^13^C and ^1^H NMR signals were supported by 2‐D NMR techniques where necessary.

### HPLC equipment and methods

HPLC method to determine the purity of compounds: Pump: L‐7100, degasser: L‐7614, autosampler: L‐7200, UV detector: L‐7400, interface: D‐7000, data transfer: D‐line, data acquisition: HSM‐Software (all from LaChrom, Merck Hitachi); Equipment 2: Pump: LPG‐3400SD, degasser: DG‐1210, autosampler: ACC‐3000T, UV‐detector: VWD‐3400RS, interface: DIONEX UltiMate 3000, data acquisition: Chromeleon 7 (Thermo Fisher Scientific); column: LiChropher® 60 RP‐select B (5 μm), LiChroCART® 250–4 mm cartridge; flow rate: 1.0 mL/min; injection volume: 5.0 μL; detection at λ=210 nm; solvents: A: demineralized water with 0.05 % (V/V) trifluoroacetic acid, B: acetonitrile with 0.05 % (V/V) trifluoroacetic acid; gradient elution (% A): 0–4 min: 90 %; 4–29 min: gradient from 90 % to 0 %; 29–31 min: 0 %; 31–31.5 min: gradient from 0 % to 90 %; 31.5–40 min: 90 %. Unless otherwise noted, the purity of all test compounds is greater than 95 %.

### Synthetic procedures

The compounds 6a and 7a have been reported in ref..^[65]^ The procedures have been modified and are described in the Supporting Information.

### Benzyl 4‐oxo‐3,4‐dihydropyridine‐1(*2H*)‐carboxylate (7b)

Iodoxybenzoic acid (IBX with 20 % benzoic acid as stabilizer, 2.77 g, 11.8 mmol, 1.3 eq) and 4‐methylmorpholin‐4‐oxide (NMO), (4.32 g, 36 mmol, 3.4 eq) were dissolved in DMSO (15 mL) and the piperidone **6b** (2.73 g, 10.8 mmol) dissolved in DMSO (20 mL) was added to the solution. The mixture was stirred for 72 h at 30 °C in the dark. The reaction mixture was poured into a saturated solution of NaHCO_3_ (50 mL), the mixture was extracted with Et_2_O (3×50 mL) and the combined Et_2_O layers were washed with NaHCO_3_, brine and water. The organic layer was dried (Na_2_SO_4_) and concentrated in vacuo. The crude product was purified by automated fc (Snap, 340 g, V=1600 mL, CH_2_Cl_2_ : ethyl acetate=9 : 1, Rf=0.44). Colorless solid, mp 65 °C, yield 2.26 g (83 %) C_13_H_13_NO_3_ (231.3 g/mol). HR‐MS (APCI): m/z=232.0975 (calcd. 232.0968 for C_13_H_14_NO_3_ [M+H]^+^). ^1^H NMR (600 MHz, CDCl_3_): δ (ppm)=2.56 (t, *J=*7.3 Hz, 2H, 3‐H), 4.04 (t, *J=*7.4 Hz, 2H, 2‐H), 5.26 (bs, 2H, CH_2_‐bnz), 5.34 (bs, 1H, 5‐H), 7.33–7.44 (m, 5H, H_arom._), 7.85 (bs, 1H, 6‐H). ^13^C NMR (151 MHz, CDCl_3_): δ (ppm)=35.8 (C‐3), 42.7 (C‐2), 69.2 (CH_2_‐bnz), 107.9 (C‐5), 127.1, 128.6, 128.7, 128.9, 129.0 (5 C, C_arom._), 135.0 (C‐1_arom._), 141.1 (N*C*OO‐benz), 143.3 (C‐6), 193.5 (C‐4). Purity (HPLC): 87.2 %, *t*_R_=17.6 min.

### *tert*‐Butyl 4‐oxo‐3,4‐dihydropyridine‐1(*2H*)‐carboxylate (7c)

Iodoxybenzoic acid (IBX with 20 % benzoic acid as stabilizer, 4.3 g, 13 mmol, 1.3 eq) and 4‐methylmorpholin‐4‐oxide (NMO), (3.5 g, 30 mmol, 3.0 eq) were dissolved in DMSO (15 mL) and the piperidone **6c** (1.99 g, 10.1 mmol) dissolved in DMSO (20 mL) was added to the solution. The mixture was stirred for 70 h at 30 °C in the dark. The reaction mixture was poured into a saturated solution of NaHCO_3_ (50 mL), the mixture was extracted with Et_2_O (3×50 mL) and the combined Et_2_O layers were washed with NaHCO_3_, brine and water. The organic layer was dried (Na_2_SO_4_) and concentrated in vacuo. The crude product was purified by automated fc (Snap, 340 g, V=540 mL, CH_2_Cl_2_ : ethyl acetate=9 : 1, R_f_=0.44). Colorless solid, mp 53 °C, yield 1.51 g (77 %) C_10_H_15_NO_3_ (197.2 g/mol). HR‐MS (APCI): m/z=198.1125 (calcd. 198.1161 for C_10_H_15_O_3_ [M+H]^+^). ^1^H NMR (400 MHz, DMSO‐d_6_): δ (ppm)=7.83 (d, *J=*8.2 Hz, 1H, 6‐H), 5.18 (d, *J=*8.2 Hz, 1H, 5‐H), 3.92–3.84 (m, 2H, 2‐CH_2_), 2.48–2.41 (m, 2H, 3‐CH_2_), 1.48 (s, 9H 3x CH_3_). ^13^C NMR (151 MHz, DMSO‐d_6_): δ (ppm)=28.2 (3 C, CH_3_), 35.8 (C‐3), 42.4 (C‐2), 83.7 (C‐(CH_3_)_3_) 106.4 (C‐5), 144.2 (C‐6), 154.6 (COOR), 193.8 (C‐4). Purity (HPLC): 86.7 %, *t*_R_=16.5 min.

### 2‐Phenyl‐1‐tosylpiperidin‐4‐one (8a)

Phenylboronicacid (1.46 g, 11.9 mmol, 3.0 eq) and [Rh(cod)_2_]BF_4_ (64.0 mg, 0.16 mmol, 0.04eq) were dissolved in degassed H_2_O/dioxane (1 : 11, 12 mL) and the mixture was stirred for 30min. Enone **7a** (1.02g, 4.06mmol, 1.0 eq) dissolved in H_2_O/dioxane (1 : 11, 8 mL) was added dropwise and the mixture was heated to 85 °C for 5 h. The mixture was filtered through a short pad of silica gel with Et_2_O washing, the filtrate was dried (Na_2_SO_4_), concentrated in vacuo and the crude product was purified by automated fc (Snap, 100 g, V=200 mL, diethyl ether/cyclohexane=4 : 1, R_f_=0.55). Yellow resin, yield 460mg (34 %). C_18_H_19_NO_3_S (329.4 g/mol). HR‐MS (APCI): m/z=330.1184 (calcd. 330.1158 for C_18_H_20_NO_3_S [M+H]+). ^1^H NMR (400 MHz, CDCl3): δ [ppm]=2.25 (ddt, *J=*15.5/3.8/2.1 Hz, 1H, 5‐H_eq_), 2.39–2.45(m, 1H, 5‐H_ax_), 2.45 (s, 3H, CH_3_), 2.72 (dd, *J=*15.3/7.0 Hz, 1H, 3‐H_ax_), 2.93 (dt, *J=*15.3/1.9 Hz, 1H, 3‐H_eq_), 3.14 (ddd, *J=*14.5/12.1/3.6 Hz, 1H, 6‐H_ax_), 4.01 (ddt, *J=*14.5/6.9/2.1 Hz, 1H, 6‐H_eq_), 5.63 (d, *J=*7.1 Hz, 1H, 2‐H), 7.21–7.37 (m, 7H, 3‐H_Tos_, 5‐H_Tos_, 5× H_phenyl_), 7.79–7.86 (m, 2H, 2‐H_Tos_, 6‐H_Tos_).^13^C NMR (151 MHz, CdCl_3_): δ [ppm]=δ 21.7 (CH_3_), 40.4 (C‐5), 40.4 (C‐6), 43.5 (C‐3), 56.6 (C‐2), 127.3 (2 C, C‐2_Tos_, C‐6_Tos_), 127.5 (2 C, C‐2_phenyl_, C‐6_phenyl_), 128.2 (C‐4_phenyl_), 128.9 (2 C, C‐3_phenyl_, C‐5_phenyl_), 130.2 (2 C, C‐3_Tos_, C‐5_Tos_), 137.6 (C‐4_Tos_), 138.5 (C‐1_Tos_), 144.2 (C‐1_phenyl_), 206.4 (C‐4). Purity (HPLC): 96.6 %, 22.1 min. FT‐IR (neat): *ν* [cm^−1^]=2971 (C‐Harom.), 1715 (C=O), 1152 (SO2 N).

### Benzyl 4‐oxo‐2‐phenylpiperidine‐1‐carboxylate (8b)

Phenylboronicacid (688 mg, 5.6mmol, 1.3eq) and [Rh(cod)_2_]BF_4_ (36.8 mg, 0.09 mmol, 0.02eq) were dissolved in a mixture of degassed KOH (1.5 M, 2 mL) and dioxane (6 mL) and the mixture was stirred for 30min at rt. Enone **7b** (1.0g, 4.3mmol, 1.0eq) dissolved as well in a mixture of KOH (2 mL) and dioxane (6 mL) was added dropwise to the first mixture and heated to 90 °C for 7 h. After cooling down to rt, brine (45 mL) was added and the mixture was extracted with CH_2_Cl_2_ (4×, 40 mL). The combined organic layers were dried (Na_2_SO_4_), concentrated *in vacuo* and the crude product was purified by automated fc (Snap, 100 g, V=400 mL, cyclohexane : ethyl acetate=75 : 25, R_f_=0.26). Yellow resin, yield 954mg (71 %). C_19_H_19_NO_3_ (309.4). HR‐MS (APCI): m/z=310.1446 (calcd. 310.1438 for C_19_H_20_NO_3_ [M+H]^+^). ^1^H NMR (400 MHz, CDCl_3_): δ [ppm]=2.30–2.43 (m, 1H, 5‐H), 2.54 (ddd, *J=*15.9/11.3/6.7 Hz, 1H, 5‐H), 2.86 (ddd, *J=*15.5/6.9/0.8 Hz, 1H; 3‐H_ax_), 2.99 (ddd, *J=*15.5/3.3/1.4 Hz, 1H, 3‐H_eq_), 3.20 (ddd, *J=*14.5/11.3/3.9 Hz, 1H, 6‐H), 4.29 (d, *J=*10.7 Hz, 1H, 6‐H), 5.16–5.29 (m, 2H, CH_2_‐bnzl), 5.84 (bs, 1H, 2‐H), 7.19–7.39 (m, 10H, H_arom._).^13^C NMR (101 MHz, CdCl_3_): δ [ppm]=39.1 (C‐6), 40.7 (C‐5), 44.3 (C‐3), 54.8 (C‐2), 68.0 (CH_2_‐ph), 126.8, 127.1, 127.8, 127.9, 128.1, 128.4, 128.68, 128.71, 129.0 (10 C, C_arom._), 136.4 (C‐1_benzyl_), 139.8 (C‐1_phenyl_), 155.6 (N*C*OO‐bnz), 207.4 (C‐4). Purity (HPLC): 99.9 %, t_R_=20.5 min.

### Ethyl (*E*)‐ and (*Z*)‐2‐(2‐phenyl‐1‐tosylpiperidin‐4‐ylidene)acetate (9a)

Piperidone **8a** (624 mg, 1.89 mmol) was dissolved in dry toluene (8 mL) Then Ph_3_P=CHCO_2_Et (1.05g, 3.01mmol, 1.6eq) was added and the mixture was heated to reflux for 18h. The solvent was removed in vacuo and the crude product was purified by automated fc (Snap 100 g, V=200 mL, cyxlohexane : ethyl acetate=75 : 25, R_f_=0.77 and 0.67). The diastereomers (*Z*)‐**9a** and (*E*)‐**9a** were not separated. Colorless resin, yield 782 mg (103 %, Ph_3_P=O impurity). C_22_H_25_NO_4_S (399.5). HR‐MS (APCI): m/z=400.1601 (calcd. 400.1577 for C_22_H_26_NO_4_S [M+H]+). ^1^H NMR (600 MHz, CDCl_3_): δ [ppm]=1.22 (t, *J=*7.2 Hz, 1.2H, *OCH_2_ *C*H_3_), 1.27 (t, *J=*7.2 Hz, 1.8H, ^#^OCH_2_ *C*H_3_), 2.03 (d, *J=*13.9 Hz, 0.6H, 5‐H_eq_^#^), 2.19–2.28 (m, 1H, 5‐H_ax_^#^, 5‐H_ax_*), 2.30 (dd, *J=*14.5/6.2 Hz, 0.6H, 3‐H_ax_^#^), 2.42 (s, 1.2 H, *CH_3Tos_), 2.44 (s, 1.8H, ^#^CH_3Tos_), 2.61 (dd, *J=*14.5/6.2 Hz, 0.4H, 3‐H_ax_*), 2.74 (dd, *J=*14.5/2.9 Hz, 0.4H, 3‐H_eq_*), 3.05 (ddd, *J=*14.1/12.1/3.3 Hz, 0.6H, 6‐H_ax_^#^), 3.12 (ddd, *J=*14.1/12.1/3.3 Hz, 0.4H, 6‐H_ax_*), 3.49 (dt, *J=*15.5/3.0 Hz, 0.6H, 5‐H_eq_*), 3.86 (ddd, *J=*14.1/5.0/3.1 Hz, 0.4H, 6‐H_eq_*), 3.92 (dtd, *J=*14.0/3.5/2.0 Hz, 0.6H, 6‐H_eq_^#^), 4.04–4.12 (m, 0.8H, *OC*H_2_* CH_3_), 4.16 (qd, *J=*7.1/3.1 Hz, 1.2H, *OC*H_2_* CH_3_), 4.42 (d, broad, *J=*15.0 Hz, 0.6H, 3‐H_eq_^#^), 5.33 (dd, *J=*6.3/2.8 Hz, 0.4H, 2‐H_eq_*), 5.42 (d, *J=*6.0 Hz, 0.6H, 2‐H_eq_^#^), 5.64 (s, 0.6H, ^#^=C*H*CO_2_R), 5.70 (s, 0.4H, *=C*H*CO_2_R), 7.19–7.25 (m, 1H, H‐4_phenyl_^#^*), 7.25–7.34 (m, 4.8H, 2‐H_phenyl_*, 6‐H_phenyl_*, 3‐H_phenyl_^#^*, 5‐H_phenyl_^#^*, 3‐H_Tos_^#^*, H‐5_Tos_^#^*), 7.40 (d, *J=*8.2 Hz, 1.2H, 2‐H_phenyl_^#^, 6‐H_pheny_^#^_l_), 7.69–7.74 (m, 0.8H, 2‐H_Tos_*, 6‐H_Tos_*), 7.77–7.84 (m, 1.2H, 2‐H_Tos_^#^, 6‐H_Tos_^#^). The ratio of diastereomers (*Z*)‐**9a**:(*E*)‐**9a** is 60 : 40. Signals of (*Z*)‐**9a** are marked with ^#^, signals of (*E*)‐**9a** with *. ^13^C NMR (151 MHz, CDCl_3_) δ [ppm]=14.3*, 14.4 (OCH_2_ *C*H_3_ *)*, 21.67*, 21.69 (CH_3Tos_ *)*, 28.4*, 34.9 (C‐5), 30.4, 38.5* (C‐3), 41.1*, 41.6 (C‐6), 56.37, 56.42* (C‐2), 60.01*, 60.04 (O*C*H_2_CH_3_), 117.6, 117.8* (=*C*HCO_2_R), 127.19, 127.21, 127.23, 127.31, 127.44, 127.57 (5 C, C‐4_‐phenyl_, C‐2_phenyl_, C‐6_phenyl_, C‐2_Tos_, C‐6_Tos_), 128.6, 128.7* (2 C, C‐3_phenyl_, C‐5_phenyl_), 130.0*, 130.1 (2 C, C‐3_Tos;_ C‐5_Tos_), 137.8*, 138.2 (C‐1_Tos_), 138.7, 139.4* (C‐1_phenyl_), 143.6*, 143.7 (C‐4_Tos_), 154.3 (C‐4), 166.0*, 166.2 (*C*O_2_R). Signals of the minor diastereomer (*E*)‐**9a** are marked with *. Purity (HPLC): 96.2 %, t_R_=23.4 min and 23.8 min.

### Benzyl (*E*)‐ and (*Z*)‐4‐(ethoxycarbonylmethylene)‐2‐phenylpiperidine‐1‐carboxylate (9b)

Piperidone **8b** (904.8 mg, 2.92 mmol, 1.0 eq) was dissolved in dry toluene (6 mL) Then Ph_3_P=CHCO_2_Et (2.04g, 5.85mmol, 2.0eq) was added and the mixture was heated to 115°C for 18h. The solvent was removed in vacuo and the crude product was purified by automated fc (Snap, 100 g, V=1700 mL, CH_2_Cl_2_ : ethyl acetate=4 : 1, R_f_=0.52 and 0.44). Colorless resin, yield 1.09 g (98 %). C_23_H_25_NO_4_ (379.5). HR‐MS (APCI): m/z=380.1884 (calcd. 380.1856 for C_23_H_26_NO_4_ [M+H]^+^). ^1^H NMR (400 MHz, CDCl_3_) δ [ppm]=1.27 (t, *J=*7.1 Hz, 1.35H, CH_3_*), 1.29 (t, *J=*7.1 Hz, 1.65H, CH_3_^#^), 2.22 (d, *J=*14.2 Hz, 0.55H, 5‐H_ax_^#^), 2.45 (td, *J=*13.3/12.8, 5.6 Hz, 0.55H, 5‐H_eq_^#^), 2.54–2.68 (m, 1H, 3‐H^#^, 5‐H*), 2.74–2.88 (m, 0.9H, 3‐H*, 6‐H*), 2.97 (ddd, *J=*13.3/11.9/3.5 Hz, 0.55H, 6‐H_eq_^#^), 3.19 (td, *J=*12.5/2.5 Hz, 0.45H, 3‐H_eq_*), 3.44 (d, *J=*16.8 Hz, 0.45H, 5‐H_eq_*), 4.13–4.20 (m, 2.55H, 6‐H^#^, C*H_2_* CH_3_*^#^), 4.44 (d, *J=*15.2 Hz, 0.55H, 3‐H_eq_), 5.07–5.27 (m, 2H, C*H_2_*‐bnz), 5.42 (s, broad, 0.45H, 2‐H*), 5.68 (s, broad, 0.55H, 2‐H^#^), 5.74 (s, 0.55H, =C*H*COOR^#^), 5.79 (s, 0.45H, =C*H*COOR*), 7.11–7.44 (m, 10H, H_arom._). The ratio of diastereomers (*Z*)‐**9b**:(*E*)‐**9b** is 55 : 45. Signals of (*Z*)‐**9b** are marked with ^#^, signals of (*E*)‐**9b** with *.^13^C NMR (101 MHz, CdCl3): δ [ppm]=14.3, 14.4* (*C*H_3_), 27.1*, 28.2 (C‐5), 31.02, 31.04* (C‐3), 37.3, 40.5* (C‐6), 51.4*, 54.9 (C‐2), 59.9*, 60.9 (*C*H_2_CH_3_), 66.8*, 67.5 (*C*H_2_‐bnz), 117.4, 125.2* (=*C*HCOOR), 127.3, 127.7, 128.06, 128.111, 128.16, 128.40, 128.5, 128.60, 128.63, 128.76 (10 C, C_arom._), 131.2, 131.1* (C‐1_phenyl_), 136.78, 136.80* (C‐1_bnz_), 140.5, 140.7* (N*C*OOR), 155.22, 155.24* (C‐4), 171.1*, 171.2 (C=O). Signals of the minor diastereomer (*E*)‐**9b** are marked with *. Purity (HPLC): 98.4 %, t_R_=23.5 min and 23.8 min.

### Ethyl *cis*‐ and *trans*‐2‐(2‐phenyl‐1‐tosyl‐piperidin‐4‐yl)acetate (10)

A solution of α,β‐unsaturated ester **9a** (738mg, 1.80mmol) in CH_3_OH (20mL) was added to a suspension of Pd/C (10 %, 197mg, 0.18mmol, 0.1eq.) in CH_3_OH (5mL) and the mixture was stirred for 20h under H_2_ (1 bar). Then, the mixture was filtered through Celite® 45 and the filtrate was concentrated in vacuo. The crude product was purified by automated fc (Snap, 100 g, cyclohexane : ethyl acetate=75 : 25, V=360 mL, R_f_=0.53 and 0.47). Colorless oil, yield 591mg (81 %). C_22_H_27_NO_4_S (401.5). HR‐MS (APCI): m/z=402.1759 (calcd. 402.1734 for C_22_H_28_NO_4_S [M+H]^+^). ^1^H NMR (600 MHz, CDCl_3_): δ [ppm]=0.85 (m, 0.35H, H‐5_ax_*), 1.01 (qd, *J=*12.7/4.6 Hz, 0.65H, H‐5_ax_^#^), 1.20 (t, *J=*7.1 Hz, 1H, OCH_2_ *C*H3*), 1.23 (t, *J=*7.2 Hz, 2H, OCH_2_ *C*H3^#^), 1.33 (ddd, *J=*13.7/12.2,/5.3 Hz, 0.65H, 3‐H^#^_ax_), 1.37–146 (m, 1H, H‐5_eq_^#^*), 1.67 (ddd, *J=*13.8/9.7/3.8 Hz, 0.35H, 3‐H*_ax_), 1.85–2.04 (m, 1.35H 3‐H*_eq_, 4‐H^#^*), 2.03–2.20 (m, 2H, *C*H_2_CO^#^*), 2.31–2.38 (m, 0.65H, 3‐H^#^_eq_), 2.41 (s, 1.05H, CH_3Tos_*), 2.44 (s, 1.95H, CH_3Tos_^#^), 2.99 (ddd, *J=*14.7/13.1/2.9 Hz, 0.65H, 6‐H_ax_^#^), 3.10 (ddt, *J=*12.9/8.4/4.2 Hz, 0.35H, 6‐H_ax_*), 3.88 (m, 1H, 6‐H_eq_^#^*), 4.02–4.15 (m, 2H, O*C*H_2_CH_3_^#^*), 4.18 (dd, *J=*9.7/4.3 Hz, 0.35H, 2‐H_ax_*), 5.34 (d, *J=*5.2 Hz, 0.65H, 2‐H_eq_^#^), 7.17–7.39 (m, 7H, H_phenyl_^#^*, H‐3_Tos_^#^*, H‐5_Tos_^#^*), 7.44–7.49 (m, 0.7H, H‐2_Tos_*, H‐6_Tos_*), 7.72–7.79 (m, 1.3H, H‐2_Tos_^#^, H‐6_Tos_^#^). The ratio of diastereomers *cis*‐**10**:*trans*‐**10** is 35 : 65. Signals of *trans*‐**10** are marked with ^#^, signals of *cis*‐**10** with *. ^13^C NMR (151 MHz, CDCl_3_): δ [ppm]=14.3*, 14.4 (OCH_2_ *C*H_3_), 21.6*, 21.7 (CH_3Tos_), 27.6, 30.9* (C‐4), 30.7*, 30.8 (C‐5), 33.1, 39.6* (C‐3), 39.7*, 41.0 (*C*H_2_CO), 41.5, 45.0* (C‐6), 55.2, 60.8* (C‐2), 60.5, 60.6 (O*C*H_2_CH_3_), 127.0, 127.11, 127.14, 127.4, 127.8, 128.2, 128.8,128.9, 129.4, 129.9 (9 C, C_phenyl_, C‐2_Tos_, C‐3_Tos_, C‐5_Tos_, C‐6_To_) 138.7, 138.6 (C‐1_phenyl_), 141.6*, 141.7 (C‐1_Tos_), 143.3, 143.4 (C‐4_Tos_) 172.1, 172.6* (*C*O_2_R). Signals of *cis*‐**10** are marked with *. Purity (HPLC): 67.1 %, t_R_=23.1 and 23.4 min.

### *cis*‐ and *trans*‐2‐(2‐Phenyl‐1‐tosylpiperidin‐4‐yl)ethan‐1‐ol (11)

A mixture of LiAlH_4_ (76.3 mg, 2.0 mmol, 2.0 eq) and THF (10 mL) was stirred for 10 min at 0 °C. Then a solution of **10** (405 mg, 1.05 mmol) in THF (25 mL) was added dropwise to the LiAlH_4_ suspension under ice cooling. The mixture was stirred for 20 min at 0 °C and then at rt. for 2.5 h. Under ice cooling H_2_O was added dropwise and the mixture was heated to reflux for 30 min. After cooling to rt, the mixture was filtered over Celite® 45 and the celite layer was washed with ethyl acetate. The solvent was removed in vacuo and the crude product was purified by automated fc (Snap 50 g, cylxohexane : ethyl acetate=Gradient 80 : 20 to 60 : 40, V=400 mL, R_f_=0.18 (cylxohexane : ethyl acetate=60 : 40)). Colorless resin, yield 333.8 mg (89 %). C_20_H_25_NO_3_S (359.5 g/mol). HR‐MS (APCI): m/z=360.1640 (calculated 360.1628 for C_20_H_26_NO_3_S [M+H]+). ^1^H NMR (600 MHz, CDCl_3_): δ [ppm]=0.88 (m, 0.17H, 5‐H_ax_*), 0.98 (tdd, *J=*13.1/11.9/4.6 Hz, 0.83H, 5‐H_ax_^#^), 1.35 (ddd, *J=*13.8/12.2/5.4 Hz, 0.85 Hz, 3‐H_ax_^#^), 1.30–1.40 (m, 1.83H, 3‐H_ax_^#^*, *C*H_2_CH_2_OH^#^), 1.41–1.48 (m, 1H, 5‐H_eq_^#^*), 1.50–1.70 (m, 1H, 4‐H), 1.84–1.89 (m, 0.34H, *C*H_2_CH_2_OH*) 2.29 (ddt, *J=*13.8/3.5/2.0 Hz, 1H, 3‐H_eq_), 2.40 (s, 0.5H, *CH_3_), 2.44 (s, 2.5H, CH_3_), 2.99 (ddd, *J=*14.5/13.1/2.9 Hz, 0.83H, 6‐H_ax_^#^), 3.07 (ddd, *J=*12.9/8.7/4.3 Hz, 0.17H, 6‐H_ax_*), 3.56–3.67 (m, 2H, CH_2_OH^#^*), 3.84–3.93 (m, 1H, 6‐H_eq_^#^*), 4.10–4.14 (m, 0.17H, 2‐H*), 5.34 (d, *J=*4.8 Hz, 0.83H, 2‐H_eq_^#^), 7.18–7.25 (m, 2H, 2x H_phenyl_), 7.28–7.34 (m, 5H, 3‐H_Tos_, 5‐H_Tos_, 3x H_phenyl_), 7.46 (d, *J=*8.3 Hz, 0.34H, 2‐H_Tos_*, 6‐H_Tos_*), 7.74–7.78 (m, 1.66H, 2‐H_Tos_, 6‐H_Tos_). The ratio of diastereomers *trans*‐**11**:*cis*‐**11** is 83 : 17. Signals of *trans*‐**11** are marked with ^#^, signals of *cis*‐**11** with *. ^13^C NMR (151 MHz, CDCl_3_): δ [ppm]=24.1, 24.2 (CH_3_), 29.7, 33.1* (C‐4), 33.2*, 33.6 (C‐5), 36.4, 43.0 (C‐3), 41.0*, 41.9 (*C*H_2_CH_2_OH), 44.2, 47.8* (C‐6), 57.8, 63.8* (C‐2), 62.7, 62.8* (*C*H_2_OH), 129.4, 129.5, 129.7, 129.82, 129.85, 130.4, 130.6, 131.3, 131.8, 132.3 (9 C, 5x C_phenyl_, 4× C_Tos_), 138.3*, 141.3 (C‐1_Tos_), 141.5, 144.6* (C‐1_phenyl_), 145.6*, 145.7 (C‐4_Tos_). Signals of *cis*‐**11** are marked with *. Purity (HPLC): 82.3 %, t_R_=20.2 min, 20.4 min.

### *cis*‐ and *trans*‐2‐(2‐Phenyl‐1‐tosylpiperidin‐4‐yl)ethyl methanesulfonate (12)

A solution of alcohol **11**(130 mg, 0.36 mmol) in CH_2_Cl_2_ (10 mL) was cooled to 0 °C, Et_3_N (170 μL, 1.23 mmol, 3.4 eq) was added and the solution was stirred for 10 min under ice cooling before methanesulfonyl chloride (40 μL, 0.52 mmol, 1.5 eq) was added. The reaction mixture was stirred at rt. for 18 h. Then, the mixture was washed with NaOH (2 x, 0.5 M, 5 mL) and NH_4_Cl (5 mL), dried (Na_2_SO_4_) and the solvent was removed in vacuo. The crude product was purified with automated fc (Snap 50 g, cyclohexane : ethyl acetate=50 : 50, V=200 mL, R_f_=0.48). Colorless resin, yield 148 mg (94 %). C_21_H_27_NO_5_S_2_ (437.6 g/mol). HR‐MS (APCI): m/z=438.1422 (calcd 438.1403 for C21H28NO5S2 [M+H]+). ^1^H NMR (600 MHz, CDCl_3_): δ [ppm]=0.88 (m, 0.17H, 5‐H_ax_*), 1.03 (qd, *J=*13.1/4.6 Hz, 0.83H, 5‐H_ax_^#^), 1.38 (ddd, *J=*13.7/12.3/5.4 Hz, 1H, 3‐H_ax_^#^*), 1.46 (dt, *J=*13.3/2.9 Hz, 0.83H, 5‐H_eq_^#^), 1.56 (q, *J=*6.8 Hz, 2H, C*H_2_* CH_2_OSO_2_^#^*), 1.66 (dddt, *J=*14.4/11.8/5.3/2.7 Hz, 1H, 4‐H), 1.87 (m, 0.34H, 3‐H_eq_*, 5‐H_eq_*) 2.31 (dq, *J=*13.7, 2.2 Hz, 0.83H, 3‐H_eq_^#^), 2.41 (s, 0.5H, *CH_3Tos_), 2.44 (s, 2.5H, CH_3Tos_), 2.93 (s, 2.5H, SO_2_ *C*H_3_), 2.95 (s, 0.5H, *SO_2_ *C*H_3_), 3.00 (ddd, *J=*14.5/13.1/2.9 Hz, 0.83H, 6‐H_ax_^#^), 3.10 (ddd, *J=*12.9/8.5,/4.3 Hz, 0.17H, 6‐H_ax_*), 3.87 (ddd, *J=*13.1/6.6/4.4 Hz, 0.17H, 6‐H_eq_*), 3.89–3.95 (d, broad, *J=*14.2 Hz, 0.83H, 6‐H_eq_^#^), 4.19 (ddt, *J=*10.0, 6.4 Hz, 2H, *C*H_2_OSO_2_), 4.11–4.17 (m, 0.17H, 2‐H*), 5.36 (d, *J=*4.9 Hz, 0.83H, 2‐H_eq_^#^), 7.17–7.25 (m, 2H, 3‐H_Tos_^#^*, 5‐H_Tos_^#^*), 7.28–7.34 (m, 5H, H_pheny_^#^*_l_), 7.43–7.48 (m, 0.34H, 2‐H_Tos_*, 6‐H_Tos_*), 7.72–7.79 (m, 1.66H, 2‐H_Tos_^#^, 6‐H_Tos_^#^). The ratio of diastereomers *trans*‐**12**:*cis*‐**12** is 83 : 17. Signals of *trans*‐**12** are marked with ^#^, signals of *cis*‐**12** with *. ^13^C NMR (151 MHz, CDCl_3_): δ [ppm]=21.68*, 21.70 (CH_3Tos_), 27.1*, 27.2 (C‐4), 30.9 (C‐5), 33.5 (C‐3), 34.7*, 35.7 (*C*H_2_CH_2_OSO_2_CH_3_), 37.5, 37.6 (SO_2_ *C*H_3_), 41.5, 44.9* (C‐6), 55.1, 60.8* (C‐2), 66.9, 67.4* (*C*H_2_OSO_2_CH_3_), 126.9, 127.0, 127.1, 127.3*, 127.4*, 127.8*, 128.2*, 128.9, 129.5*, 129.9 (9 C, 5x C_phenyl_, 4× C_Tos_), 135.8*, 138.5 (C‐1_Tos_), 138.6, 141.7* (C‐1_phenyl_), 143.33, 143.38* (C‐4_Tos_). Signals of *cis*‐**12** are marked with *. Purity (HPLC): 99.6 %, t_R_=21.9 min, 22.1 min.

### *cis*‐ and *trans*‐*N*‐Benzyl‐2‐(2‐phenyl‐1‐tosyl‐piperidin‐ 4‐yl)ethan‐1‐amine (13a)

Mesylate **12** (200 mg, 0.45 mmol) was dissolved in CH_3_CN (15 mL), dest. benzylamine (147 μL, 1.35 mmol, 3.0 eq) was added and the reaction mixture was stirred under reflux for 18 h. The solvent was removed in vacuo, the crude product was dissolved in ethyl acetate and the solution was washed with 0.5 M NaOH (2 x, 10 mL), dried (Na2SO4) and concentrated in vacuo. The crude product was purified by fc (d=2 cm, l=18 cm, V=35 mL, CH_2_Cl_2_ : MeOH : NH_3_=94 : 5 : 1, R_f_=0.43 and 0.35). Colorless solid, mp 79 °C, yield 122 mg (60 %). C_27_H_32_N_2_O_2_S (448.6 g/mol). HR‐MS (APCI): m/z=449.2228 (calcd. 449.2257 for C_27_H_33_N_2_O_2_S [M+H]^+^). ^1^H NMR (600 MHz, CDCl_3_): δ [ppm]=0.86–0.98 (qd, *J=*12.1/4.9 Hz, 0.35H, 5‐H_ax_*), 1.25–1.40 (m, 3.35H, H‐3_ax_*, H‐5_ax_^#^, 5‐H_eq_*, C*H_2_* CH_2_NH^#^*), 1.43–1.50 (m, 1H, 4‐H^#^*), 1.54 (dt, *J=*13.5/10.3 Hz, 0.65H, 3‐H_ax_^#^), 1.78–1.85 (m, 1.3H, 5‐H_eq_^#^, 3‐H_eq_^#^), 2.24 (ddt, *J=*13.8/3.6/2.0 Hz, 0.35H, 3‐H_eq_*), 2.39 (s, 1.95H, CH_3Tos_), 2.43 (s, 1.05H, CH_3Tos_*), 2.48–2.62 (m, 2H, CH_2_ *C*H_2_NH^#^*), 2.96 (ddd, *J=*14.5/13.2/2.9 Hz, 0.35H, 6‐H_ax_*), 3.08 (ddd, *J=*12.9/8.4/4.4 Hz, 0.65H, 6‐H_ax_^#^), 3.71 (s, 1.3H, N*C*H_2_‐ph), 3.72 (s, 0.7H, N*C*H_2_‐ph), 3.84 (ddd, *J=*12.8/6.6/4.5 Hz, 0.65H, 6‐H_eq_^#^), 3.86–3.91 (m, 0.35H, 6‐H_eq_*), 4.13 (dd, *J=*10.1/4.5 Hz, 0.65H, 2‐H_ax_^#^), 5.32 (d, *J=*4.5 Hz, 0.35H, 2‐H_eq_*), 7.16–7.23–7.33 (m, 12H, 3‐H_Tos_, 5‐H_Tos_, 5x H_phenyl_, 5x H_benzyl_), 7.43–7.48 (m, 1.3H, 2‐H_Tos_^#^, 6‐H_Tos_^#^), 7.73–7.77 (m, 0.7H, 2‐H_Tos_*, 6‐H_Tos_*). The ratio of diastereomers *cis*‐**13a**:*trans*‐**13a** is 65 : 35. Signals of *cis*‐**13a** are marked with ^#^, signals of *trans*‐**13a** with *. ^13^C NMR (151 MHz, CDCl_3_): δ [ppm]=21.6, 21.7* (CH_3_), 28.4*, 31.9 (C‐4*), 30.7, 33.7* (C‐3), 31.1, 40.4 (C‐5*), 36.0, 37.0* (*C*H_2_CH_2_NH), 41.8*, 45.2 (C‐2), 46.4*, 46.7 (CH_2_ *C*H_2_NH), 54.1, 54.2* (*C*H_2_‐bnz), 55.3*, 61.2 (C‐2), 127.0, 127.1, 127.29, 127.31, 127.8, 128.1, 128.2, 128.3, 128.6, 128.8, 129.3, 129.8 (14 C, C_aromat_), 135.9 (C‐1_Tos_), 139.1*, 142.2 (C‐1_phenyl_), 140.2 (C‐1_bnz_), 143.1, 143.2* (C‐4_Tos_). Signals of *trans*‐**13a** are marked with *. Purity (HPLC): 96.3 %, t_R_=20.2 min, 20.5 min.

### *cis*‐ and *trans*‐*N*‐(3‐Phenylpropyl)‐2‐(2‐phenyl‐1‐tosylpiperidin‐4‐yl)ethan‐1‐amine (13b)

Mesylate **12** (69.2 mg, 0.16 mmol,) was dissolved in CH_3_CN (8 mL), phenylpropylamine (88 μL, 0.6 mmol, 3.8 eq) was added and the reaction mixture was stirred under reflux for 18 h. The solvent was removed in vacuo, the crude product was dissolved in ethyl acetate and the solution was washed with 0.5 M NaOH (2×, 10 mL), dried (Na_2_SO_4_) and concentrated in vacuo. The crude product was purified twice by fc. First column (d=1 cm, l=18 cm, V=25 mL, CH_2_Cl_2_ : MeOH : Et_3_N=93 : 5 : 2). Second column (d=1 cm, l=18 cm, V=12 mL, CH_2_Cl_2_ : ethyl acetate:EtNMe_2_=90 : 8 : 2, R_f_=0.27). Light‐yellow resin, yield 65.6 mg (87 %). C_29_H_36_N_2_O_2_S (476.7 g/mol). HR‐MS (APCI): m/z=477.2568 (calcd. 477.2570 for C_29_H_37_N_2_O_2_S [M+H]^+^). ^1^H NMR (600 MHz, CDCl_3_) δ [ppm]=0.94 (qd, *J=*13.0/4.8 Hz, 0.35H, H‐5_ax_*), 1.22–1.48 (m, 4H, 3‐H_ax_*, 4‐H^#^*, 5‐H_ax_^#^, C*H_2_* CH_2_NH−R), 1.54 (dt, *J=*13.5/10.3 Hz, 0.65H, H‐3_ax_^#^), 1.68–1.91 (m, 3.65H, R‐NH‐CH_2_ *CH_2_* CH_2_‐phenyl, 5‐H_eq_^#^*, 3‐H_ax_^#^), 2.24 (d, *J=*13.3 Hz, 0.35H, 3‐H_eq_*), 2.39 (s, 1.95H, CH_3Tos_^#^), 2.43 (s, 1.05H, CH_3Tos_*), 2.45–2.72 (m, 6H, CH_2_ *CH_2_* NH−R, R‐NH*CH_2_* CH_2_ *CH_2_*‐phenyl), 2.96–2.98 (m, 0.35H, 6‐H_ax_*), 3.08 (ddd, *J=*12.8/8.3/4.4 Hz, 0.65H, 6‐H_ax_^#^), 3.84 (ddd, *J=*12.8/6.5/4.4 Hz, 0.65H, 6‐H_eq_^#^), 3.88–3.94 (m, 0.35H, 6‐H_eq_*), 4.08–4.19 (m, 0.65H, 2‐H_ax_^#^), 5.32 (d, *J=*5.2 Hz, 0.35H, 2‐H_eq_*), 7.11–7.33 (m, 12H, H_arom._), 7.38–7.48 (m, 1.3, 2‐H_Tos_^#^, 6‐H_Tos_^#^), 7.68–7.80 (m, 0.7H, 2‐H_Tos_*, 6‐H_Tos_*). The ratio of diastereomers *cis*‐**13b**:*trans*‐**13b** is 65 : 35. Signals of *cis*‐**13b** are marked with ^#^, signals of *trans*‐**13b** with *. ^13^C NMR (151 MHz, CdCl_3_) δ [ppm]=21.63, 21.68* (CH_3Tos_), 30.7 (C‐5), 31.7 (R‐NHCH_2_ *C*H_2_CH_2_‐phenyl), 32.0 (C‐4), 33.8 (R‐NHCH_2_CH_2_ *C*H_2_‐phenyl), 40.5 (C‐3), 41.7*, 45.2 (C‐6), 47.0*, 47.3 (CH_2_ *C*H_2_NH−R), 49.6 (R‐NH*C*H_2_CH_2_CH_2_‐phenyl), 55.3*, 61.2 (C‐2), 125.9, 126.96, 126.98, 127.2, 127.3, 127.4, 127.8, 128.1, 128.5, 128.8, 129.3, 129.8 (14 C, C_arom._), 136.0 (C‐1_Tos_), 138.8 (C‐1_phenyl_) 142.2, 142.3* (C‐1_arom._), 143.1, 143.2* (C‐4_Tos_). Where signals of *cis* and *trans* could be distinguished, signals of *trans*‐**13b** are marked with *. Purity (HPLC): 87.7 %, t_R_=20.9 min.

### *cis*‐ and *trans*‐*N*‐Benzyl‐2‐(2‐phenylpiperidin‐4‐yl)ethan‐1‐amine (4a)

Sulfonamide **13a** (30.5 mg, 0.07 mmol) and Mg^0^ turnings (27.5 mg, 1.13 mmol, 16.0 eq) were suspended in in MeOH (5 mL) and the mixture was stirred under irradiation with ultrasound for 8 h. Then the mixture was acidified with HOAc to pH=5 and then the pH‐value was adjusted to pH 10 with NH_3_. The organic layer was separated and the aqueous layer was extracted with CH_2_Cl_2_ (3×5 mL). The combined organic layers were dried (Na_2_SO_4_) and concentrated in vacuo. The crude product was purified by fc (d=1 cm, l=15 cm, V=30 mL, CH_2_Cl_2_ : MeOH : NH_3_=94 : 5 : 1, R_f_=0.15 (CH_2_Cl_2_:CH_3_OH:NH_3_=93 : 5 : 2)). Colorless resin, yield 16.1 mg (80 %). C_20_H_26_N_2_ (294.4 g/mol). HR‐MS (APCI): m/z=295.2177 (calcd. 295.2169 for C_20_H_27_N_2_ [M+H]^+^). ^1^H NMR (600 MHz, CDCl_3_): δ [ppm]=0.79–0.96 (m, 0.2H, 5‐H*), 1.17–2.03 (m, 7H, 3‐H_ax_, 3‐H_eq_, 4‐H, 5‐H_ax_, 5‐H_eq_, *C*H_2_CH_2_NH), 2.68 (q, *J=*7.7 Hz, 2H, CH_2_ *C*H_2_NH), 2.80 (td, *J=*12.1/2.6 Hz, 0.8, 6‐H), 2.90–2.31 (m, 0.2H, 6‐H*), 3.21 (ddd, *J=*11.8/4.2/2.4 Hz, 0.8H, 6‐H), 3.45–3.52 (m, 0.2H, 6‐H*), 3.63 (dd, *J=*11.3/2.5 Hz, 0.8H, 2‐H), 3.79 (s, 1.6H, *C*H_2_‐ph), 3.81 (s, 0.4H, *C*H_2_‐ph), 3.89 (dd, *J=*9.7/2.9 Hz, 0.2H, 2‐H*), 7.18–7.45 (m, 10H, H_arom_). The ratio of diastereomers *cis*‐**4a**:*trans*‐**4a** is 80 : 20. Due to low intensity, some of the signals for *trans*‐**4a** could not be detected. Signals of *trans*‐**4a** are marked with *. ^13^C NMR (101 MHz, CDCl_3_): δ [ppm]=32.1 (C‐5), 34.8 (C‐4), 37.9 (*C*H_2_CH_2_NH), 41.2 (C‐3), 46.4 (CH_2_ *C*H_2_NH), 47.1 (C‐6), 53.8 (*C*H_2_‐ph), 61.9 (C‐2), 127.0, 127.4, 127.5, 128.5, 128.6, 128.7 (10 C, C_arom_), 139.1 (C‐1_bnz_), 141.3 (C_phenyl_). Signals of *trans*‐**4 a** are not visible in the ^13^C NMR spectrum. Purity (HPLC): 99.0 %, t_R_=11.9 min.

### *cis*‐ and *trans*‐*N*‐(3‐Phenylpropyl)‐2‐(2‐phenyl‐piperidin‐ 4‐yl)ethan‐1‐amine (4b)

Sulfonamide **13b** (27.6 mg, 0.06 mmol) and Mg^0^ turnings (28.3 mg, 1.16 mmol, 20.0 eq) were suspended in in MeOH (5 mL) and the mixture was irradiated with ultrasound for 5 h. Then, the mixture was acidified with HOAc to pH=5 and then, the pH‐value was adjusted to pH =10 with NH_3_. The organic layer was separated and the aqueous layer was extracted with CH_2_Cl_2_ (3×5 mL). The combined organic layers were dried (Na_2_SO_4_) and concentrated in vacuo. The crude product was purified by fc (d=1 cm, l=18 cm, V=25 mL, CH2Cl2 : ethyl acetate:EtNMe_2_=90 : 8 : 2, R_f_=0.13). Brown resin, yield 6.9 mg (37 %). C_22_H_30_N_2_ (322.5 g/mol). HR‐MS (APCI): m/z=323.2486 (calcd. 323.2482 for C C_22_H_31_N_2_ [M+H]^+^). ^1^H NMR (600 MHz, CDCl_3_) δ [ppm]=1.16–1.26 (m, 2H, 3‐H_ax_, 5‐H_ax_), 1.48 (tq, *J=*13.8/6.7/6.2 Hz, 2H, *C*H_2_CH_2_NH−R), 1.59 (m, 0.75H, H‐4^#^), 1.72 (d, broad, *J=*15.4 Hz, 1H, 5‐H_eq_), 1.75‐1.85 (m, 3H, 3‐H_eq_, R‐NH‐CH_2_ *CH_2_* CH_2_‐ph), 1.90–1.94 (m, 0.25H, 4‐H*), 2.64 (m, 6H, R‐NH‐*CH_2_* CH_2_ *CH_2_*‐ph, CH_2_ *CH_2_* NH−R), 2.80 (td, *J=*12.1/2.6 Hz, 0.75H, 6‐H_ax_^#^), 2.93 (dt, *J=*12.2/4.3 Hz, 0.25H, 6‐H_ax_*), 2.99 (td, *J=*11.7/2.9 Hz, 0.25H, 6‐H_eq_*), 3.22 (ddd, *J=*11.6/4.1/2.6 Hz, 0.75H, 6‐H_eq_^#^), 3.60 (dd, *J=*11.2/2.4 Hz, 0.75H, 2‐H_ax_^#^), 3.89 (dd, *J=*10.4/2.8 Hz, 0.25H, 2‐H_ax_*), 7.13–7.38 (m, 10H, H_arom_^#^*). The ratio of diastereomers *cis*‐**4b**:*trans*‐**4b** is 75 : 25. Signals of *cis*‐**4b** are marked with ^#^, signals of *trans*‐**4b** with *. ^13^C NMR (151 MHz, CDCl_3_) δ [ppm]=30.4*, 32.7 (C‐5), 31.6*, 31.7 (R‐NHCH_2_ *C*H_2_CH_2_‐ph), 33.8, 33.8* (R‐NHCH_2_CH_2_ *C*H_2_‐ph), 35.1 (C‐4), 37.4 (*C*H_2_CH_2_NH−R), 38.7*, 41.8 (C‐3), 42.3*, 47.4 (C‐6) 47.3, 48.4* (CH_2_ *C*H_2_NH−R), 49.7, 49.8 (R‐NH*C*H_2_CH_2_CH_2_‐ph), 56.1*, 62.1 (C‐2), 125.9, 126.8, 127.3, 128.48, 128.50, 128.55 (10 C, C_arom_, C_phenyl_), 142.2 (C‐1_arom_), 145.0*, 145.3 (C‐1_phenyl_). Signals of *trans*‐**4b** are marked with *, where they could be distinguished from signals of *cis*‐**4b**. Purity (HPLC): 97.8 % t_R_=14.2 min.

### Ethyl *cis*‐ and *trans*‐2‐(2‐phenylpiperidin‐4‐yl)‐acetate (14)

A solution ion of α,β‐unsaturated ester **9b** (2.99 g, 1.0mmol) in CH_3_OH (27mL) was added to a suspension of Pd/C (10 %, 841.0 mg, 0.79mmol, 0.1eq.) in CH_3_OH (3mL) and the mixture was stirred for 20h under H_2_ (3 bar). Then, the mixture was filtered through Celite® 45 and concentrated in vacuo. The crude product was purified by fc (d=6 cm, h=16 cm, V=1500 mL, CH_2_Cl_2_ : MeOH : dimethylethylamin,=97 : 2 : 1, R_f_=0.27). Colorless resin, yield 1.37 g (70 %). C_15_H_21_NO_2_ (247.3). HR‐MS (APCI): m/z=248.1635 (calcd. 248.1645 for C_15_H_22_NO_2_ [M+H]^+^). ^1^H NMR (600 MHz, CH_3_OD): δ [ppm]=1.22–1.29 (m, 3H, CH_3_^#^*), 1.26–1.35 (m, 1.5H, 5‐H_ax_^#^, 3‐H_ax_^#^), 1.51–1.58 (d, broad, *J=*13.4 Hz, 0.25H, 5‐H_ax_*), 1.68–1.73 (m, 0.25H, 3‐H_ax_*), 1.78 (dt, *J=*13.8/2.7 Hz, 0.75H, 5‐H_eq_^#^), 1.81–1.90 (m, 1H, 5‐H_eq_*, 3‐H_eq_^#^), 1.91–2.03 (m, 0.25H, 3‐H_eq_*), 2.09 (dddt, *J=*15.6/11.5/7.9/3.9 Hz, 0.75H, 4‐H^#^), 2.24–2.35 (m, 1.5H, C*H_2_* COOR^#^), 2.39–2.49 (m, 0.25H, 4‐H*), 2.59 (d, broad *J=*7.6 Hz, 0.5H, C*H_2_* COOR*), 2.82 (td, *J=*12.5/2.9 Hz, 0.75H, 6‐H_ax_^#^), 2.90–3.00 (m, 0.5H, 6‐H_ax_*,6‐H_eq_*), 3.14–3.28 (d, broad *J=*12.5 Hz, 0.75H, 6‐H_eq_^#^), 3.66 (dd, *J=*11.6/2.5 Hz, 0.75H, 2‐H_ax_^#^), 3.92 (dd, *J=*10.6/2.9 Hz, 0.25H, 2‐H_ax_*), 4.14 (dqd, *J=*14.2/7.1/1.2 Hz, 2H, C*H_2_* CH_3_^#^*), 7.14–7.41 (m, 5H, H_phenyl_^#^*). The ratio of diastereomers *cis*‐**14**:*trans*‐**14** is 75 : 25. Signals of *cis*‐**14** are marked with ^#^, signals of *trans*‐**14** with *. ^13^C NMR (151 MHz, CD_3_OD): δ [ppm]=14.57*, 14.60 (CH_3_), 30.4*, 32.6 (C‐5), 30.5*, 35.1 (C‐4), 38.2*, 42.4 (*C*H_2_COOR), 38.3*, 41.3 (C‐3) 42.5*, 47.5 (C‐6), 56.6*, 62.6 (C‐2), 61.4, 61.5* (*C*H_2_CH_3_), 126.9, 127.7, 128.2, 128.4, 129.4, 129.5 (5 C, C_phenyl_), 144.7*, 145.0 (C‐1_phenyl_), 174.2 174.6* (*C*=O). Signals of *trans*‐**14** are marked with *. Purity (HPLC): 84.4 %, t_R_=13.9 min.

### Ethyl *cis*‐ and *trans*‐2‐(1‐methyl‐2‐phenyl‐piperidin‐ 4‐yl)acetate (15a)

NaBH(OAc)_3_ (2.46 g, 11.6 mmol, 3.0 eq) was added to a stirred solution of formalin (37 %, 866 μL, 11.6 mmol, 3.0 eq) and amine **14** (960 mg, 3.88 mmol) in CH_2_Cl_2_ (25 mL). The reaction mixture was stirred over night at rt. A saturated solution of NaHCO_3_ (20 mL) was added. The organic layer was separated and the aqueous layer was extracted with CH_2_Cl_2_ (3×20 mL). The combined organic layers were dried (NaSO_4_), filtered and concentrated in vacuo. The crude product was purified by fc (d=3 cm, l=16 cm, V=270 mL, cyclohexane : ethyl acetate=3 : 1+1 % dimethylethylamine, R_f_=0.31). Colorless oil, yield 671 mg (66 %). C_16_H_23_NO_2_ (261.4). HR‐MS (APCI): m/z=262.1823 (calcd. 262.1802 for C_16_H_24_NO_2_ [M+H]^+^). ^1^H NMR (400 MHz, CDCl_3_): δ [ppm]=1.23 (t, *J=*7.1 Hz, 2.25H, CH_3_^#^), 1.26 (t, *J=*7.1 Hz, 0.75H, CH_3_*), 1.30–1.42 (m, 0.75H, 3‐H_ax_^#^), 1.43–1.55 (m, 0.75H, 5‐H_ax_^#^), 1.61 (m, 0.5H, 3‐H_ax_*, 5‐H_ax_*), 1.75–1.83 (m, 1.5H, 3‐H_eq_^#^, 5‐H_eq_^#^), 1.91–2.00 (m, 0.75H, 4‐H^#^), 2.01 (s, 2.25H, N‐CH_3_^#^), 2.03 (s, 0.75H, N‐CH_3_*), 2.15–2.26 (m, 2.25H, C*H_2_* COOR^#^, 6‐H_ax_^#^), 2.26–2.40 (m, 0.25H, 6‐H_ax_*), 2.38–2.52 (m, 0.25H, 4‐H*), 2.51–2.55 (m, 0.5H, C*H_2_* COOR*), 2.85 (d, *J=*11.4 Hz, 1H, 2‐H_ax_^#^, 6‐H_eq_*), 3.04 (m, 1H, 2‐H_ax_*, 6‐H_eq_^#^), 4.04–4.17 (m, 2H, C*H_2_* CH_3_^#^*), 7.23 (dt, *J=*8.5/4.2 Hz, 1H, 4‐H_phenyl_^#^*), 7.31 (d, *J=*4.8 Hz, 4H, 2‐H_phenyl_^#^*, 3‐H_phenyl_^#^*, 5‐H_phenyl_^#^*, 6‐H_phenyl_^#^*). The ratio of diastereomers *cis*‐**15a**:*trans*‐**15a** is 75 : 25. Signals of *cis*‐**15a** are marked with ^#^, signals of *trans*‐**15a** with *. ^13^C NMR (101 MHz, cdcl_3_): δ [ppm]=14.39, 14.43* (CH_3_), 28.9*, 33.7 (C‐4), 32.3, 39.6* (C‐5), 36.6*, 41.4 (*C*H_2_COOR), 42.1 (C‐3), 44.2, 44.5* (N‐CH_3_), 51.7*, 57.0 (1 C, C‐6), 60.4, 60.5* (CO_2_ *C*H_2_CH_3_), 65.3*, 70.5 (C‐2), 127.3, 127.6, 128.6 (5 C, C_phenyl_), 144.1 (C‐1_phenyl_), 172.7, 173.1* (*C*=O). Signals of *trans*‐**15 a** are marked with *. Purity (HPLC): 98.9 %, t_R_=14.5 min.

### Ethyl *cis*‐ and *trans*‐2‐(1‐ethyl‐2‐phenyl‐piperidin‐4‐yl)acetate (15b)

NaBH(OAc)_3_ (58.6 mg, 0.28 mmol, 1.6 eq) was added to a solution of acetaldehyde (12.3 mg, 0.28 mmol, 1.6 eq) and amine **14** (40 mg, 0.17 mmol, 1.0 eq) in CH_2_Cl_2_ (5 mL). The reaction mixture was stirred for 18 h at rt, before a saturated solution of NaHCO_3_ (10 mL) and CH_2_Cl_2_ (5 mL) was added. The organic layer was separated and the aqueous layer was extracted with CH_2_Cl_2_ (3×10 mL). The combined organic layers were dried (NaSO_4_), filtered and concentrated in vacuo. The crude product was purified by fc (d=3 cm, l=12 cm, V=30 mL, CH_2_Cl_2_:MeOH=97 : 2+1 % Et_3_N, R_f_=0.23). Pale yellow oil, yield 30 mg (68 %). C_15_H_21_NO_2_ (275.4). HR‐MS (APCI): m/z=276.1972 (calcd. 276.1958 for C_15_H_23_NO [M+H]^+^). ^1^H NMR (400 MHz, CDCl_3_): δ [ppm]=00.92 (t, *J=*7.0 Hz, 3H, N‐CH_2_‐C*H_3_*), 1.23 (t, *J=*7.1 Hz, 3H, CO_2_CH_2_C*H_3_*), 1.25–1.35 (m, 0.8H, 5‐H_ax_^#^), 1.35–1.48 (m, 0.8H, 3‐H_ax_^#^), 1.53–1.62 (m, 0.4 H, 3‐H_ax_*, 5‐H_ax_*), 175 −1.88 (m, 1.8H, 3‐H_eq_^#^, 5‐H_eq_^#^, 3‐H_eq_*), 1.89–2.07 (m, 1.4H, 4‐H^#^, 5‐H_eq_*, N‐*CH_2_* CH_3_*), 2.08–2.18 (m, 0.8H, 6‐H_ax_^#^), 2.17–2.28 (m, 2H, C*H_2_* COOR^#^*), 2.32– 2.46 (m, 0.4 H, 4‐H*, 6‐H_ax_*) 2.47–2.61 (m, 1.6H, N‐C*H_2_* CH_3_^#^), 2.93–3.03 (m, 0.2H, 6‐H_eq_*), 3.09 (dd, *J=*2.6/11.2 Hz, 0.8H, 2‐H_ax_^#^), 3.18 (dt, *J=*3.6/11.6 Hz, 0.8H, 6‐H_eq_^#^), 3.29 (dd, *J=*11.1/2.8 Hz, 0.2 H, 2‐H_ax_*), 4.02–4.18 (m, 2H, CO_2_ *CH_2_* CH_3_^#^*), 7.22 (m, 1H, 4‐H_phenyl_^#^*), 7.27–7.34 (m, 4H, 2‐H_phenyl_^#^*, 3‐H_phenyl_^#^*, 5‐H_phenyl_^#^*, 6‐H_phenyl_^#^*). The ratio of diastereomers *cis*‐**15b**:*trans*‐**15b** is 80 : 20. Signals of *cis*‐**15b** are marked with ^#^, signals of *trans*‐**15b** with *. ^13^C NMR (101 MHz, CdCl_3_): δ [ppm]=11.3, 11.5* (N‐CH_2_‐*CH_3_*), 14.4, 14.5* (CO_2_CH_2_ *CH_3_*), 29.1*, 33.8 (C‐4), 29.5*, 42.9 (C‐5), 32.4, 36.9* (C‐3), 41.5 (*C*H_2_COOR), 46.7*, 52.0 (C‐6), 48.8, 49.0* (N‐*C*H_2_‐CH_3_), 60.3, 60.4* (CO_2_ *C*H_2_CH_3_), 62.9*, 68.2 (C‐2), 127.1, 127.6, 127.61, 128.5, 128.6 (5 C, C_phenyl_), 144.9 (C‐1_phenyl_), 172.8 (C=O). Signals of *trans*‐**15b** are marked with *.Purity (HPLC): 80.8 %, t_R_=15.4 min.

### *cis*‐ and *trans*‐2‐(1‐Methyl‐2‐phenylpiperidin‐4‐yl)ethan‐1‐ol (16a)

A solution of ester **15a** (400 mg, 1.53 mmol) in THF (15 mL) was added dropwise to an ice‐cooled suspension of LiAlH_4_ (123 mg, 3.25 mmol, 2.1 eq) in THF (20 mL). The mixture was stirred for 30 min at 0 °C. Ice cooling was removed and the reaction mixture was stirred for 2 h at rt. H_2_O was added under ice cooling until the gas formation has stopped and the mixture was heated to reflux for 30 min. After cooling to rt, the organic layer was separated and the aqueous layer was extracted with EtOAc (3 x 10 mL). The combined organic layers were dried (Na_2_SO_4_), filtered and concentrated in vacuo. The crude product was purified by fc (d=3 cm, l=15 cm, V=140 mL, CH_2_Cl_2_:MeOH=97 : 2+1 % Et_3_N, R_f_=0.23). Colorless oil, yield 287 mg (85 %). C_14_H_21_NO (219.3). HR‐MS (APCI): m/z=220.1701 (calcd. 220.1696 for C_14_H_22_NO [M+H]^+^). ^1^H NMR (600 MHz, CDCl_3_): δ [ppm]=1.33 (m, 0.8H, 3‐H_ax_^#^), 1.40–1.48 (m, 1H, 5‐H_ax_^#^*), 1.48–1.58 (m, 2H, C*H_2_* CH_2_OH), 1.58–1.69 (m, 1H, 4‐H^#^, 3‐H_ax_*), 1.75–1.83 (m, 1.6H, 3‐H_eq_^#^, 5‐H_eq_^#^), 1.83–1.91 (m, 0.2H, 5‐H_eq_*), 2.01 (d, *J=*6.8 Hz, 3H, N‐CH_3_^#^*), 2.17 (t, *J=*11.9 Hz, 0.8H, 6‐H_ax_^#^), 2.37 (t, *J=*11.7 Hz, 0.2H, 6‐H_ax_*), 2.81 (m, 1H, 2‐H_ax_^#^, 6‐H_eq_*), 3.01–3.10 (m, 1H, 6‐H_eq_^#^, 2‐H_ax_*), 3.68 (dt, *J=*11.7/5.3 Hz, 2H, CH_2_C*H_2_* OH^#^*), 7.24 (tt, *J=*5.9/3.1 Hz, 1H, 4‐H_phenyl_^#^*), 7.31 (d, *J=*5.2 Hz, 4H, 2‐H_phenyl_^#^*, 3‐H_phenyl_^#^*, 5‐H_phenyl_^#^*, 6‐H_phenyl_^#^*). The ratio of diastereomers *cis*‐**16a**:*trans*‐**16a** is 80 : 20. Signals of *cis*‐**16a** are marked with ^#^, signals of *trans*‐**16a** with *. ^13^C NMR (151 MHz, CDCl_3_): δ [ppm]=29.8*, 42.5 (C‐3), 32.7 (C‐5), 33.1, 34.2* (C‐4), 39.6, 39.9* (*C*H_2_CH_2_OH), 44.3, 44.5* (N‐CH_3_), 51.9*, 57.3 (C‐6), 60.5, 61.5* (CH_2_ *C*H_2_OH), 65.5*, 68.5 (C‐2), 127.2, 127.56, 127.62, 128.6, 128.6 (5 C, C_phenyl_), 144.5 (C‐1_phenyl_). Signals of *trans*‐**16a** are marked with *. Purity (HPLC): 99.6 %, t_R_=10.4 min and 10.5 min.

### *cis*‐ and *trans*‐2‐(1‐Ethyl‐2‐phenylpiperidin‐4‐yl)ethan‐1‐ol (16b)

A solution of ester **15b** (535 mg, 1.95 mmol) in THF (15 mL) was added dropwise to an ice‐cooled suspension of LiAlH_4_ (158 mg, 4.17 mmol, 2.1 eq) in THF (15 mL). The reaction was stirred for 30 min at 0 °C. Ice cooling was removed and the reaction mixture was stirred for 2 h at rt. H_2_O was added under ice cooling until the gas formation has stopped and the mixture was heated to reflux for 30 min. After cooling to rt, K‐Na‐tartrate (10 mL) was added to the mixture and the organic layer was separated and the aqueous layer was extracted with EtOAc (3×10 mL). The combined organic layers were dried (Na_2_SO_4_), filtered and concentrated in vacuo. The crude product was purified by automated flash column chromatography (Snap, 100 g, V=200 mL, CH_2_Cl_2_:EtOAc=3 : 1+1 % ethyldimethylamine, R_f_=0.2). Colorless resin, yield 287 mg (85 %). C_15_H_23_NO (233.4). HR‐MS (APCI): m/z=234.1858 (calcd. 234.1852 for C_15_H_24_NO [M+H]^+^). ^1^H NMR (600 MHz, CDCl_3_): δ [ppm]=0.91 (t, *J=*7.1 Hz, 3H, N‐CH_2_‐C*H_3_*), 1.26–1.34 (m, 1H, 3‐H_ax_), 1.38 (qd, *J=*12.5/3.9 Hz, 1H, 5‐H_ax_), 1.43–1.58 (m, 2H, C*H_2_* CH_2_OH), 1.62 (m, 1H, 4‐H), 1.73–1.84 (m, 2 H, 5‐H_eq_, 3‐H_eq_), 1.97 (dq, *J=*13.7/7.0 Hz, 1H, N‐*CH_2_*‐CH_3_), 2.10 (td, *J=*12.0/2.7 Hz, 0.85H, 6‐H_ax_^#^), 2.25–2.35 (m, 0.15H, 6‐H_ax_*), 2.54 (dq, *J=*13.7/7.4 Hz, 2H, N‐*CH_2_*‐CH_3_), 2.92–2.97 (m, 0.15 H, 6‐H_eq_*), 3.05 (dd, *J=*11.2/2.8 Hz, 0.85H, 2‐H_ax_^#^), 3.19 (dt, *J=*3.5/11.6 Hz, 0.85H, 6‐H_eq_^#^), 3.27–3.32 (m, 0.15 H, 2‐H_ax_*), 3.68 (t, *J=*6.7 Hz, 1.7H, CH_2_C*H_2_* OH^#^), 3.71 (t, *J=*6.7 Hz, 0.3H, CH_2_C*H_2_* OH*), 7.22 (tt, *J=*6.3/2.2 Hz, 1H, 4‐H_phenyl_), 7.27–7.35 (m, 4H, H‐H_phenyl_, 3‐H_phenyl_, 5‐H_phenyl_, 6‐H_phenyl_). The ratio of diastereomers *cis*‐**16b**:*trans*‐**16b** is 85 : 15. Signals of *cis*‐**16b** are marked with ^#^, signals of *trans*‐**16b** with *.^13^C NMR (151 MHz, CDCl_3_): δ [ppm]=11.3, 11.4* (N‐CH_2_‐*C*H_3_), 28.3*, 33.2 (C‐4), 29.8*, 32.7 (C‐5), 34.4*, 39.7 (*C*H_2_CH_2_OH), 40.4*, 43.3 (C‐3), 46.8*, 52.2 (1 C, C‐6), 48.9, 49.2* (N‐*C*H_2_‐CH_3_), 60.6, 61.5* (CH_2_ *C*H_2_OH), 63.0*, 68.5 (C‐2), 126.9, 127.0, 127.5, 127.6, 128.5, 128.6 (5 C, C_phenyl_), 145.2 (C‐1_phenyl_). Signals of *trans*‐**16b** are marked with *. Purity (HPLC): 91.1 %, t_R_=11.3 min.

### *cis*‐ and *trans*‐2‐(1‐Methyl‐2‐phenylpiperidin‐4‐yl)acetaldehyde (17a)

A solution of alcohol **16a** (197 mg, 0.90 mmol, 1.0 eq) in CH_2_Cl_2_ (5 mL) was added to a solution of Dess‐Martin Periodinane (576 mg, 1.36 mmol, 1.5 eq) in CH_2_Cl_2_ (3 mL). The reaction mixture was stirred for 5 h at rt, before a solution of saturated NaHCO_3_ and 10 % Na_2_S_2_O_3_ (1 : 1, 8 mL) was added. The organic layer was separated and the aqueous layer was extracted with CH_2_Cl_2_ (3×10 mL). The combined organic layers were dried (Na_2_SO_4_), filtered and concentrated in vacuo. The crude product was purified by fc (d=2 cm, l=18 cm, V=90 mL, cyclohexane : ethyl acetate=3 : 2+1.5 % ethyldimethylamine, R_f_=0.30 and 0.37 first and second diastereomer). Yellow resin, yield 122 mg (62 %). C_14_H_19_NO (217.3). HR‐MS (APCI): m/z=218.1533 (calcd. 218.1539 for C_14_H_20_NO [M+H]^+^). ^1^H NMR (400 MHz, CDCl_3_): δ [ppm]=1.33–1.68 (m, 2H, 3‐H_ax_^#^*, 5‐H_ax_^#^*), 1.80 (dt, *J=*13.6/3.0 Hz, 1.3H, 3‐H_eq_^#^, 5‐H_eq_^#^), 2.04 (s, 3H, N‐CH_3_^#^*), 1.89–2.18 (m, 1.35H, 4‐H^#^, 3‐H_eq_*, 5‐H_eq_*), 2.19–2.37 (m, 1H, 6‐H_ax_^#^*), 2.38 (ddd, *J=*6.4/3.3/1.8 Hz, 1.3H, CH_2_CHO^#^), 2.59 (m, 0.35H, 4‐H*), 2.62–2.73 (m, 0.7H, CH_2_CHO*), 2.88 (t, *J=*12.3 Hz, 1H, 2‐H_ax_^#^, 6‐H_eq_*), 3.00 (d, *J=*11.7 Hz, 0.35H, 2‐H_ax_*), 3.09 (d, *J=*11.7 Hz, 1H, 6‐H_eq_^#^), 7.20–7.35 (m, 5H, H_phenyl_), 9.77 (t, *J=*2.0 Hz, 0.65H, CHO^#^), 9.79 (t, *J=*2.0 Hz, 0.35H, CHO*). The ratio of diastereomers *cis*‐**17a**:*trans*‐**17a** is 65 : 35. Signals of *cis*‐**17a** are marked with ^#^, signals of *trans*‐**17a** with *. ^13^C NMR (101 MHz, CDCl_3_): δ [ppm]=26.3*, 31.3 (C‐4) 29.6*, 32.4 (C‐5), 39.7*, 42.1 (C‐3), 44.1, 44.4* (N‐CH_3_), 45.8*, 50.5 (CH_2_CHO), 51.6*, 56.9 (C‐6), 65.4*, 70.5 (C‐2), 127.40, 127.47, 127.55, 127.6, 128.65, 128.70 (5 C, C_phenyl_), 143.6 (C‐1_phenyl_), 201.9, 202.1* (CHO). Signals of *trans*‐**17a** are marked with *. Purity (HPLC) Rkt 180: 98.3 %, t_R_=10.3 min and 10.9 min.

### *cis*‐ and *trans*‐2‐(1‐Ethyl‐2‐phenylpiperidin‐4‐yl)acetaldehyde (17b)

Dess‐Martin Periodinane (409 mg, 0.96 mmol, 1.5 eq) was added to a solution of alcohol **16b** (149 mg, 0.64 mmol) in CH_2_Cl_2_ (4 mL). The reaction mixture was stirred for 1.5 h at rt. Then 10 % Na_2_S_2_O_3_ (4 mL) and a saturated solution of NaHCO_3_ (4 mL) was added and the mixture was stirred for 10 min. The organic layer was separated and the aqueous layer was extracted with ethyl acetate (3×10 mL). The combined organic layers were dried (Na_2_SO_4_) and concentrated in vacuo. The crude product was purified by automated fc (Snap, 25 g, V=100 mL, CH_2_Cl_2_:EtOAc=4 : 1+2 % ethyldimethylamine, R_f_=0.32). Orange resin, yield 137 mg (93 %). C_15_H_21_NO (231.3). HR‐MS (APCI): m/z=232.1685 (calcd. 232.1695 for C_15_H_22_NO [M+H]^+^). ^1^H NMR (600 MHz, CDCl_3_): δ [ppm]=0.91 (m, 3H, N‐CH_2_‐*CH_3_*), 1.34 (q, *J=*12.2 Hz, 0.85H, 3‐H_ax_^#^), 1.39–1.65 (m, 1.15H, 5‐H_ax_^#^*, 3‐H_ax_*), 1.73–1.84 (m, 1.7H, 3‐H_eq_^#^, 5‐H_eq_^#^), 1.83–1.94 (m, 0.3H, 3‐H_eq_*, 5‐H_eq_*), 1.98 (tt, *J=*13.7/6.7 Hz, 2H, N‐*CH_2_*‐CH_3_^#^*), 2.01–2.10 (m, 1H, 4‐H^#^*), 2.11–2.18 (m, 0.85H, 6‐H_ax_^#^), 2.25–2.43 (m, 2.15H, CH_2_CHO^#^*, 6‐H_ax_*), 2.49–2.57 (m, 1H, N‐*CH_2_*‐CH_3_^#^*), 2.97 (dt, *J=*12.5/4.0 Hz, 0.15H, 6‐H_eq_*), 3.07–3.12 (m, 0.85H, 2‐H_ax_^#^), 3.19 (m, 0.85H, 6‐H_eq_^#^) 3.22–3.28 (m, 0.15H, 2‐H_ax_*), 7.22 (m, 1H, 4‐H_phenyl_^#^*), 7.26–7.34 (m, 4H, H_phenyl_^#^*), 9.73–9.76 (m, 0.85H, CHO^#^), 9.73–9.76 (m, 0.15H, CHO*). The ratio of diastereomers *cis*‐**17b**:*trans*‐**17b** is 85 : 15. Signals of *cis*‐**17b** are marked with ^#^, signals of *trans*‐**17b** with *.^13^C NMR (151 MHz, CDCl_3_): δ [ppm]=11.2, 11.4* (N‐CH_2_‐*CH_3_*), 29.7*, 32.5 (C‐5), 31.5 (C‐4), 40.1*, 43.0 (C‐3), 46.0*, 48.8 (N‐*CH_2_*‐CH_3_), 46.6*, 51.9 (C‐6), 50.7 (CH_2_CHO), 62.9*, 68.2 (C‐2), 127.0, 127.1, 127.5, 127.6, 128.5 (5 C, C_phenyl_), 144.7 (C‐1_phenyl_), 202.1, 202.3* (CHO). Signals of *trans*‐**17b** are marked with *. Purity (HPLC): 94.0 %, t_R_=11.5 min.

### *cis*‐ and *trans*‐*N*‐Benzyl‐2‐(1‐methyl‐2‐phenyl‐piperidin‐ 4‐yl)ethan‐1‐amine (18a)

Benzylamine (49.3 mg, 0.46 mmol, 2.5 eq) and aldehyde **17a** (20.6 mg, 0.09, 0.5 eq) were solved in CH_2_Cl_2_ (3 mL) and the mixture was stirred for 1 h at rt. Then, additional 0.5 equivalents of aldehyde **17a** dissolved in CH_2_Cl_2_ were added and the mixture was stirred for 1 h, before NaBH(OAc)_3_ (97.5 mg, 0.46 mmol, 2.5 eq) was added to the solution. The reaction mixture was stirred over night at rt. Then a saturated solution of NaHCO_3_ (6 mL) was added and the aqueous layer was extracted with ethyl acetate (3×10 mL). The combined organic layers were dried (Na_2_SO_4_), filtered and concentrated in vacuo. The crude product was purified twice by fc (1. d=1 cm, l=20 cm, V=12 mL, cyclohexane : ethyl acetate=1 : 1+1.5 % ethyldimethylamine; and 2. d=1 cm, l=20 cm, V=30 mL, cyclohexane : ethyl acetate=1 : 1+1 % ethyldimethylamine, R_f_=0.26). Yellow resin, yield 18.2 mg (31 %). C_21_H_28_N_2_ (308.5). MS (APCI): m/z=309.2337 (calcd. 309.2325 for C_21_H_29_N_2_ [M+H]^+^). ^1^H NMR (600 MHz, CDCl_3_): δ [ppm]=1.24–1.36 (m, 1H, 3‐H_ax_), 1.37–1.61 (m, 4H, 5‐H, 4‐H, *CH_2_* CH_2_N), 1.73 (d, broad, *J=*13.3 Hz, 2H, 3‐H, 5‐H), 2.00 (m, 3H, N‐CH_3_), 2.14 (td, *J=*12.2/2.6 Hz, 0.85H, 6‐H_ax_^#^), 3.37 (t, *J=*11.0 Hz, 0.15H, 6H_ax_*), 2.60–2.70 (m, 2H, CH_2_ *CH_2_* N), 2.79 (d, broad, *J=*11.3 Hz, 1H, 2‐H_ax_^#^, 6‐H_eq_*), 3.04 (dt, *J=*12.0/3.2 Hz, 1H, 6‐H_eq_^#^, 2‐H_ax_*), 3.77 (s, 1.7H, NC*H_2_*‐bnz^#^), 3.81 (s, 0.3H, NC*H_2_*‐bnz*), 7.20–7.25 (m, 2H, 4‐H_phenyl_), 7.28–7.35 (m, 8H, H_phenyl_). The ratio of diastereomers *cis*‐**18a**:*trans*‐**18a** is 85 : 15. Signals of *cis*‐**18a** are marked with ^#^, signals of *trans*‐**18a** with *. ^13^C NMR (151 MHz, CDCl_3_): δ [ppm]=29.7*, 32.8 (C‐5), 34.6, (C‐4), 37.1 (*CH_2_* CH_2_N), 39.9*, 42.6 (C‐3), 44.3, 44.5* (N‐CH_3_), 46.9, 47.9* (CH_2_‐*CH_2_*‐N), 54.3 (NCH_2_‐bnz), 51.9*, 57.3 (C‐6), 65.4*, 70.8 (C‐2), 127.1, 127.2, 127.6, 128.26, 128.28, 128.5, 128.6, 128.9 (8 C, C_benzyl_, C_phenyl_), 140.5 (C‐1_phenyl_), 144.6 (C‐1_benzyl_). Signals of *trans*‐**18a** are marked with *. Purity (HPLC): 95.6 %, t_R_=21.1 min.

### *cis*‐ and *trans*‐*N*‐Benzyl‐2‐(1‐ethyl‐2‐phenyl‐piperidin‐ 4‐yl)ethan‐1‐amine (18b)

A solution of aldehyde **17b** (49.3 mg, 0.21 mmol, 1.0 eq) in CH_2_Cl_2_ (3 mL) was added dropwise over 30 min to a solution of benzylamine (46.0 mg, 0.43 mmol, 2.0 eq) in CH_2_Cl_2_ (1 mL) and the reaction mixture was stirred for 3 h at rt, before NaBH(OAc)_3_ (91.1 mg, 0.43 mmol, 2.0 eq) was added to the solution. The reaction mixture was stirred over night at rt. Then a saturated solution of NaHCO_3_ (6 mL) was added to the solution and the aqueous layer was extracted with CH_2_Cl_2_ (3×10 mL). The combined organic layers were dried (Na_2_SO_4_), filtered and concentrated in vacuo. The crude product was purified three times by fc. First by automated fc (Snap, 10 g, V=50 mL, CH_2_Cl_2_:EtOAc=7 : 3+2 % ethyldimethylamine). Second (d=1 cm, l=22 cm, V=28 mL, cyclohexane : ethyl acetate=7 : 3+1 % ethyldimethylamine) and third (d=1 cm, l=22 cm, V=7 mL, cyclohexane : ethyl acetate=2 : 1+1 % ethyldimethylamine, R_f_=0.13). Yellow resin, yield 4.8 mg (7 %). C_22_H_30_N_2_ (322.5). HR‐MS (APCI): m/z=323.2476 (calcd. 323.2482 for C_22_H_31_N_2_ [M+H]^+^). ^1^H NMR (400 MHz, CDCl_3_): δ [ppm]=0.84–0.97 (m, 3H, N‐CH_2_‐*CH_3_*), 1.21–1.41 (m, 2H, 3‐H_ax_, 5‐H_ax_), 1.40–1.62 (m, 3H, 4‐H, *CH_2_*‐CH_2_‐N), 1.67–1.80 (m, 2H, 3‐H_eq_, 5‐H_eq_), 1.93–2.04 (m, 2H, N‐*CH_2_*‐CH_3_), 2.09 (td, *J=*11.7/2.0 Hz, 0.9H, 6‐H_ax_^#^), 2.35 (t, *J=*12.1 Hz, 0.1H, 6‐H_ax_*), 2.48–2.61 (m, 2H, N‐*CH_2_*‐CH_3_), 2.61–2.71 (m, 2H, CH_2_‐*CH_2_*‐N), 2.89–2.97 (m, 0.1H, 6‐H_eq_*), 3.04 (dd, *J=*11.2/2.6 Hz, 0.9H, 2‐H_ax_^#^), 3.19 (dt, *J=*11.4/3.5 Hz, 0.9H, 6‐H_eq_^#^), 3.28–3.36 (m, 0.1H, 2‐H*), 3.77 (s, 1.8H, CH_2_‐bnz^#^), 3.81 (s, 0.2H, CH_2_‐bnz*), 7.20–7.36 (m, 10H, H_arom._). The ratio of diastereomers *cis*‐**18b**:*trans*‐**18b** is 90 : 10. Where signals could be distinguished, signals of *cis*‐**18b** are marked with ^#^, signals of *trans*‐**18b** with *.^13^C NMR (151 MHz, CDCl_3_): δ [ppm]=32.7 (C‐5), 34.7 (C‐4), 37.2 (*CH_2_*‐CH_2_‐N), 43.4 (C‐3), 47.0 (CH_2_‐*CH_2_*‐N), 49.0 (N‐*CH_2_*‐CH_3_), 52.2 (C‐6), 54.3 (CH_2_‐bnz), 68.6 (C‐2), 126.85, 126.95, 126.98 127.1, 127.56, 127.65, 128.2, 128.3, 128.5, 128.6 (10 C, C_phenyl_, C_benzyl_), 140.7 (C‐1_benzyl_), 145.3 (C‐1_phenyl_). Purity (HPLC): 96.1 %, t_R_=12.7 min and 11.5 min first and second diastereomer.

### *cis*‐*N*‐(2‐(1‐Methyl‐2‐phenylpiperidin‐4‐yl)‐ethyl)‐3‐ phenylpropan‐1‐amine (19a)

3‐Phenylpropan‐1‐amine (62.2 mg, 0.46 mmol, 2.5 eq) and aldehyde **17 a** (20.6 mg, 0.09, 0.5 eq) were dissolved in CH_2_Cl_2_ (3 mL) and the mixture was stirred for 1 h at rt. Then, additional 0.5 equivalents of aldehyde **17a**, dissolved in CH_2_Cl_2_ were added and the reaction mixture was stirred for 1 h, before NaBH(OAc)_3_ (97.5 mg, 0.46 mmol, 2.5 eq) was added to the solution. The reaction mixture was stirred over night at rt. Then a saturated solution of NaHCO_3_ (6 mL) was added and the aqueous layer was extracted with ethyl acetate (3×10 mL). The combined organic layers were dried (Na_2_SO_4_), filtered and concentrated in vacuo. The crude product was purified twice by fc (d=1 cm, l=20 cm, V=75 mL, cyclohexane : ethyl acetate=1 : 1+1.5 % ethyldimethylamine) and (d=1 cm, l=22 cm, V=40 mL, cyclohexane : ethyl acetate=1 : 1+1.5 % ethyldimethylamine, R_f_=0.09). The first diastereomer and dialkylated by‐product eluted together from the column. Just one diastereomer was isolated. Colorless resin, yield 7.1 mg (11 %). C_23_H_32_N_2_ (336.5). HR‐ MS (APCI): m/z=337.2637 (calcd. 337.2638 for C_23_H_33_N_2_ [M+H]^+^). ^1^H NMR (600 MHz, CDCl_3_): δ [ppm]=1.24–1.35 (m, 1H, 3‐H_ax_), 1.37–1.49 (m, 4H, 5‐H_ax_, 4‐H, *CH_2_* CH_2_‐N), 1.70–1.76 (m, 2H, 3‐H_eq_, 5‐H_eq_), 1.82 (dq, *J=*9.1/7.1 Hz, 2H, N‐CH_2_ *CH_2_* CH_2_‐ph), 2.00 (s, 3H, N‐CH_3_), 2.14 (td, *J=*12.0/2.4 Hz, 1H, 6‐H_ax_), 2.58–2.69 (m, 6H, CH_2_ *CH_2_*‐N, N‐*CH_2_* CH_2_ *CH_2_*‐phenyl, CH_2_‐ph), 2.78 (dd, *J=*11.3/2.6 Hz, 1H, 2‐H_ax_), 3.04 (ddd, *J=*11.6/3.9/2.7 Hz, 1H, 6‐H_eq_), 7.15–7.36 (m, 10H, H_arom._). ^13^C NMR (151 MHz, CDCl_3_): δ [ppm]=31.7 (N‐CH_2_ *CH_2_* CH_2_‐phenyl), 32.8 (C‐5), 33.8 (CH_2_‐ph), 34.6 (C‐4), 37.0 (*CH_2_* CH_2_‐N), 42.7 (C‐3), 44.3 (N‐CH_3_), 47.4 (CH_2_ *CH_2_*‐N), 49.7 (N‐*CH_2_* CH_2_CH_2_‐phenyl), 57.3 (C‐6), 70.8 (C‐2), 125.9, 127.2, 127.5, 128.8, 128.50, 128.57 (10 C, C_arom._), 142.2 (C‐1_benzyl_), 144.7 (C‐1_phenyl_). Purity (HPLC): 94.2 %, t_R_=14.2 min.

### *cis*‐*N*‐(2‐(1‐Ethyl‐2‐phenylpiperidin‐4‐yl)ethyl)‐3‐phenylpropan‐1‐amine (19b)

A solution of aldehyde **17b** (50.0 mg, 0.21 mmol) in CH_2_Cl_2_ (3 mL) was added dropwise over 30 min to a solution of 3‐phenylpropan‐1‐amine (58.1 mg, 0.43 mmol, 2.0 eq)) in CH_2_Cl_2_ (1 mL) and the mixture was stirred for 3 h at rt, before NaBH(OAc)_3_ (91.1 mg, 0.43 mmol, 2.0 eq) was added to the solution. The reaction mixture was stirred over night at rt. Then a saturated solution of NaHCO_3_ (6 mL) was added and the aqueous layer was extracted with CH_2_Cl_2_ (3×10 mL). The combined organic layers were dried (Na_2_SO_4_), filtered and concentrated in vacuo. The crude product was purified by fc (d=1 cm, l=22 cm, V=35 mL, CH_2_Cl_2_ : ethyl acetate=7 : 3+1 % ethyldimethylamine, R_f_=0.15). Yellow resin, yield 46.9 mg (62 %). C_24_H_34_N_2_ (350.6). HR‐MS (APCI): m/z=351.2817 (calcd. 351.2795 for C_24_H_35_N_2_ [M+H]^+^). ^1^H NMR (400 MHz, CDCl_3_): δ [ppm]=0.89 (t, *J=*7.1 Hz, 3H, N‐CH_2_‐*CH_3_*), 1.20–1.41 (m, 2H, 3‐H_ax_, 5‐H_ax_), 1.45 (m, 3H, 4‐H, *CH_2_* CH_2_‐NH,), 1.73 (tt, *J=*12.9/2.8 Hz, 2H, 3‐H_eq_, 5‐H_eq_), 1.82 (ddd, *J=*15.0/8.6/6.8 Hz, 2H, HN‐CH_2_ *CH_2_* CH_2_), 1.95 (dd, *J=*12.9/6.9 Hz, 2H, N‐*CH_2_*‐CH_3_), 2.00–2.15 (m, 1H, 6‐H_ax_), 2.52 (dq, *J=*12.8/7.4 Hz, 2H, N‐*CH_2_*‐CH_3_), 2.57–2.67 (m, 6H, HN‐*CH_2_* CH_2_ *CH_2_*, CH_2_ *CH_2_*‐NH), 3.02 (dd, *J=*11.2/2.7 Hz, 1H, 2‐H_ax_), 3.17 (dt, *J=*11.6/3.4 Hz, 1H, 6‐H_eq_), 7.12–7.33 (m, 10H, H_phenyl_). ^13^C NMR (151 MHz, CDCl_3_): δ [ppm]=δ [ppm]=11.3 (CH_3_), 31.5 (HN‐CH_2_ *CH_2_* CH_2_), 32.7 (C‐5), 33.8 (HN‐CH_2_CH_*2*_ *CH_2_*, 34.7 (C‐4), 36.9 (*CH_2_*‐CH_2_‐NH), 43.3 (C‐3), 47.4 (CH_2_ *CH_2_*‐NH), 49.0 (N‐*CH_2_* CH_3_), 49.6 (HN‐*CH_2_* CH_*2*_CH_2_), 52.2 (C‐6), 68.5 (C‐2), 125.9, 126.9, 127.5, 128.45, 128.48, 128.50 (10 C, C_phenyl_), 142.2 (C‐1_propylphenyl_), 145.2 (C‐1_phenyl_). Purity (HPLC): 98.3 %, t_R_=14.9 min.

### *cis*‐ and *trans*‐*N*‐(Cyclohexylmethyl)‐2‐(1‐methyl‐ 2‐phenylpiperidin‐4‐yl)ethan‐1‐amine (20a)

A solution of aldehyde **17a** (51.3 mg, 0.24 mmol) and cyclohexylmethylamine (65.0 mg, 0.57 mmol, 2.5 eq) in CH_2_Cl_2_ (4 mL).was stirred for 10 min, before NaBH(OAc)_3_ (97.5 mg, 0.46 mmol, 2.0 eq) was added. After 2 h, a solution of Na_2_S_2_O_3_ (10 %) and sat. NaHCO_3_ (1 : 1, 5 mL) was added. and the aqueous layer was extracted CH_2_Cl_2_ (4×10 mL). The combined organic layers were dried (Na_2_SO_4_), filtered and concentrated in vacuo. The crude product was purified by fc (d=1 cm, l=20 cm, V=28 mL, cyclohexane : ethyl acetate=3 : 2+1,5 % ethyldimethylamine, R_f_=0.11). Colorless resin, yield 48.8 mg (66 %). C_21_H_34_N_2_ (314.5). HR‐MS (APCI): m/z=315.2811 (calcd. 315.2795 for C_21_H_35_N_2_ [M+H]^+^). ^1^H NMR (600 MHz, CDCl_3_): δ [ppm]=0.88 (qd, *J=*14.0/13.1/4.1 Hz, 2H, cyclohexane), 1.09–1.27 (m, 4.17H, 3‐H_ax_*, 4× cyclohexane), 1.31 (dt, *J=*13.3/11.2 Hz, 0.83H, 3‐H_ax_^#^), 1.37–1.62 (m, 5H, 4‐H, 5‐H_ax_, *CH_2_*‐CH_2_‐N, 1× cyclohexane), 1.62–1.92 (m, 5H, 3‐H_eq_, 5‐H_eq_, 3× cyclohexane), 2.00 (d, *J=*5.7 Hz, 3H, N‐CH_3_), 2.14 (td, *J=*12.1/2.6 Hz, 1H, 6‐H_ax_^#^), 2.32–2.39 (m, 0.17H, 6‐H_ax_*), 2.42 (dd, *J=*6.8/2.3 Hz, 1.66H, N‐CH_2_‐cyclohexane^#^), 2.46 (dd, *J=*6.8/2.3 Hz, 0.34H, N‐CH_2_‐cyclohexane*) 2.56–2.65 (m, 2H, CH_2_‐*CH_2_*‐N), 2.78 (dd, *J=*11.3/2.7 Hz, 1H, 2‐H_ax_^#^, 6‐H*), 3.04 (ddd, *J=*11.7/3.9/2.7 Hz, 1H, 6‐H_eq_^#^, 2‐H_ax_*), 7.23 (dt, *J=*8.6/4.5 Hz, 1H, 4‐H_phenyl_), 7.30 (d, *J=*4.5 Hz, 4H, H_phenyl_). The ratio of diastereomers *cis*‐**20a**:*trans*‐**20a** is 83 : 17. Signals of *cis*‐**20a** are marked with ^#^, signals of *trans*‐**20a** with *. ^13^C NMR (151 MHz, CDCl3): δ [ppm]=28.7, 28.7*, 29.3, 32.4*, 34.1, 34.2* (5 C, cyclohexane), 35.3 (C‐5), 37.2 (C‐4), 39.4 (1 C, *CH_2_* CH_2_‐N), 40.4, 42.5* (C‐1_cyclohexane_), 45.2 (C‐3), 46.9, 47.1* (N‐CH_3_), 50.1, 51.2* (CH_2_‐*CH_2_‐*N), 54.4*, 59.8 (C‐6), 59.5 (N‐*C*H_2_‐cyclohexane), 67.9*, 73.3 (C‐2), 129.6*,129.7 (C‐4_phenyl_), 130.0, 130.1*, 131.0, 131.1* (4 C, C_phenyl_), 147.3 (C‐1_phenyl_). Signals of *trans*‐**20a** are marked with *. Purity (HPLC): 96.6 %, t_R_=13.3 min.

### *cis*‐ and *trans*‐*N*‐Benzyl‐*N*‐methyl‐2‐(1‐methyl‐ 2‐phenylpiperidin‐4‐yl)ethan‐1‐amine (21a)

A mixture of aldehyde **17a** (40.0 mg, 0.18 mmol, 1.0 eq), N‐methylbenzylamine (36.9 μL, 0.27 mmol, 1.5 eq) and NaBH(OAc)_3_ (57.2 mg, 0.27 mmol, 1.5 eq) in CH_2_Cl_2_ (3 mL) was stirred for 3 h at rt. Then a solution of Na_2_S_2_O_3_ (10 %) and sat. NaHCO_3_ (1 : 1, 4 mL) was added to the reaction mixture and the aqueous layer was extracted with CH_2_Cl_2_ (4×6 mL). The combined organic layers were dried (Na_2_SO_4_), filtered and concentrated in vacuo. The crude product was purified twice by fc. First column (d=1 cm, l=25 cm, V=20 mL, cyclohexane : ethyl acetate=2 : 1+1.5 % ethyldimethylamine). Second column (d=1 cm, l=30 cm, V=20 mL, cyclohexane : ethyl acetate=3 : 1+2 % ethyldimethylamine, R_f_=0.39). Colorless oil, yield 28.2 mg (49 %). C_22_H_30_N_2_ (322.5). HR‐MS (APCI): m/z=323.2490 (calcd. 323.2482 for C_22_H_31_N_2_ [M+H]^+^). ^1^H NMR (600 MHz, CDCl_3_): δ [ppm]=1.31 (d, *J=*12.2 Hz, 1H, 3‐H_ax_), 1.38–1.64 (m, 2.65H, 5‐H_ax_, 4‐H*, *CH_2_* CH_2_‐N), 1.67–1.76 (m, 2.7H, 3‐H_eq_, 5‐H_eq_, *CH_2_* CH_2_‐N*), 1.81–1.94 (m, 0.4H, 4‐H*), 2.03 (m, 3H, piperidine‐N‐CH_3_), 2.17 (m, 3H, 6‐H_ax_, N‐CH_3_^#^), 2.22 (s, 1H, N‐CH_3_*), 2.33–2.46 (m, 2H, CH_2_‐*CH_2_*‐N), 2.78–2.85 (m, 1H, 2‐H, 6‐H_ax_*), 3.00–3.10 (m, 1H, 6‐H_eq_, 2‐H_ax_*), 3.42–3.49 (m, 0.8H, N‐*CH_2_*‐pheny*), 3.48–3.55 (m, 1.2H, N‐*CH_2_*‐phenyl^#^), 7.24 (tdd, *J=*8.7/4.0/2.1 Hz, 2H, 4‐H_phenyl_), 7.27–7.36 (m, 8H, H_phenyl_). The ratio of diastereomers *cis*‐**21a**:*trans*‐**21a** is 60 : 40. Signals of *cis*‐**21a** are marked with ^#^, signals of *trans*‐**21a** with *.^13^C NMR (151 MHz, CDCl_3_): δ [ppm]=29.8 (*CH_2_* CH_2_‐N), 32.7 (C‐5), 34.6 (C‐4), 39.8*, 42.6 (C‐3), 42.4, 42.5* (N‐CH_3_), 44.3, 44.4* (N‐CH_3_‐piperidine), 52.0*, 57.3 (C‐6), 54.8, 55.9* (CH_2_ *CH_2_*‐N), 62.6 (N‐*CH_2_*‐phenyl), 65.5*, 70.9 (C‐2), 127.07, 127.11, 127.2, 127.58, 127.65, 128.32, 128.32, 128.37, 128.6, 129.2 (10 C, C_phenyl_), 139.1 (2 C, C‐1_phenyl_). Signals of *trans*‐**21a** are marked with *.Purity (HPLC): 96.9 %, t_R_=12.6 and 13.7 min.

### *cis*‐ and *trans*‐1‐(2‐(1‐Methyl‐2‐phenyl‐ piperidin‐4‐yl)ethyl)‐4‐phenylpiperazine (22a)

NaBH(OAc)_3_ (72.0 mg, 0.34 mmol, 1.5 eq) was added to a stirred solution of aldehyde **17a** (50.0 mg, 0.23 mmol, 1.0 eq) and 1‐phenylpiperazine (55.9 mg, 0.34 mmol, 1.5 eq) in CH_2_Cl_2_ (3 mL). The reaction mixture was stirred for 3 h, then quenched with a solution of Na_2_S_2_O_3_ (10 %) and sat. NaHCO_3_ (1 : 1, 5 mL). The organic layer was separated and the aqueous layer was extracted with ethyl acetate (4×10 mL). The combined organic layers wren dried (Na_2_SO_4_), filtered and concentrated in vacuo. The crude product was purified by fc (d=1 cm, l=22 cm, V=12 mL, cyclohexane : ethyl acetate=2 : 1+1,5 % ethyldimethylamine, R_f_=0.34). Colorless resin, yield 57.4 mg (69 %). C_24_H_33_N_3_ (363.6). HR‐MS (APCI): m/z=364.2727 (calcd. 364.2747 for C_24_H_34_N_3_ [M+H]^+^). ^1^H NMR (600 MHz, CDCl_3_): δ [ppm]=1.32–1.37 (m, 0.75H, 3‐H_ax_^#^), 1.44–1.55 (m, 3.75H, 4‐H^#^, 5‐H_ax_, *CH_2_* CH_2_‐N), 1.62 (dt, *J=*13.9/2.5 Hz, 0.25H, 3‐H_ax_*), 1.77 (ddt, *J=*15.9/12.7/3.9 Hz, 1.75H, 5‐H, 3‐H_eq_^#^), 1.90 (s, 0.5 H, 4‐H*, 3‐H_eq_*), 2.00–2.06 (m, 3H, N‐CH_3_), 2.13–2.20 (m, 1H, 6‐H_ax_), 2.36–2.46 (m, 2H, CH_2_ *CH_2_*‐N), 2.55–2.60 (m, 3H, CH_2_‐N‐(*CH_2_*‐CH_2_)_2_‐N‐Ph^#^), 2.60–2.68 (m, 1H, CH_2_‐N‐(*CH_2_*‐CH_2_)_2_‐N−Ph*), 2.78–2.84 (m, 1H, 2‐H_ax_), 3.04–3.10 (m, 1H, 6‐H_eq_), 3.17–3.22 (m, 3H, CH_2_‐N‐(CH_2_‐*CH_2_*)_2_‐N‐Ph^#^), 3.21–3.26 (m, 1H, CH_2_‐N‐(CH_2_‐*CH_2_*)_2_‐N−Ph*), 6.85 (qt, *J=*7.3/1.1 Hz, 1H, 4‐H_phenylpiperazine_), 6.90–6.97 (m, 2H, 2‐H_phenylpiperazine_, 6‐H_phenylpiperazine_) 7.22–7.36 (m, 7H, 3‐H_phenylpiperazine_, 5‐H_phenylpiperazine_, H_phenyl_). The ratio of diastereomers *cis*‐**22a**:*trans*‐**22a** is 75 : 25. Signals of *cis*‐**22a** are marked with ^#^, signals of *trans*‐**22a** with *. ^13^C NMR (151 MHz, CDCl3): δ [ppm]=32.8 (C‐5), 33.8 (*C*H_2_CH_2_‐N), 35.1 (C‐4), 42.6 (C‐3), 44.3 (N‐CH_3_), 49.3, 49.3* (CH_2_‐N‐(CH_2_‐*C*H_2_)_2_‐N−Ph), 53.5, 53.6* (2 C, CH_2_‐N‐(*C*H_2_‐CH_2_)_2_‐N−Ph), 56.4, 57.5* (CH_2_‐*CH_2_*‐N), 57.3 (C‐6), 65.5*, 70.8 (C‐2), 116.1, 116.2* (2 C, C‐2_phenylpiperazine_, C‐6 _phenylpiperazine_), 119.77, 119.83 (C‐4_phenylpiperazine_), 127.2, 127.56, 127.65, 128.6, 128.7, 129.22, 129.25 (7 C, C‐3_phenylpiperazine_, C‐5 _phenylpiperazine_, C_phenyl_), 144.6 (C‐1_phenyl_), 151.5 (C‐1_phenylpiperazine_). Signals of *trans*‐**22a** are marked with *.Purity (HPLC): 99.7 %, t_R_=13.8 min.

### Receptor binding studies

Receptor binding studies were performed as previously described.[^45^, ^46^, ^47^] Details are given in the Supporting Information.

### Molecular dynamics simulations

All simulations were carried out using the Pmemd modules of Amber 20,^[51]^ running on our own CPU/GPU calculation cluster. See Supporting Information for full computational details.

### Analysis of the effects of σ_1_ receptor ligand 4 a on proliferation and morphology of the human tumor cell line A427

The effects of the piperidine derivative 4a on the growth and morphology of human tumor cell lines A 427 were determined with IncuCyte® S3 Live Cell Analysis System (Essen BioScience, Ltd., Royston, Hertfordshire, UK). In particular the confluence and *IC*_50_ values were determined. Details are given in the Supporting Information.

### DU145 cell growth inhibition

Details are given in the Supporting Information.

## Supporting Information

Supporting Information contains the purity data of all test compounds, details of the receptor biding studies and computational details. Experimental details of the effects on A427 and DU145 tumor cell lines are given. Finally, ^1^H and ^13^C NMR spectra are displayed.

## Conflict of interest

The authors declare no conflict of interest.

1

## Supporting information

As a service to our authors and readers, this journal provides supporting information supplied by the authors. Such materials are peer reviewed and may be re‐organized for online delivery, but are not copy‐edited or typeset. Technical support issues arising from supporting information (other than missing files) should be addressed to the authors.

Supporting Information

## Data Availability

The data that support the findings of this study are available in the supplementary material of this article.
