# Supplementary material for: Synthesis of Aminoethyl‐Substituted Piperidine Derivatives as σ1 Receptor Ligands with Antiproliferative Properties
Source: ChemMedChem. 2022 Feb 9;17(7):e202100735. doi: 10.1002/cmdc.202100735 (PMC9303367; doi:10.1002/cmdc.202100735)
Supplement: Supplementary file 1 — Supporting Information [file CMDC-17-0-s001.pdf]

ChemMedChem

Supporting Information

Synthesis of Aminoethyl-Substituted Piperidine Derivatives as σ_1 Receptor Ligands with Antiproliferative Properties

Catharina Holtschulte, Frederik Börgel, Stefanie Westphälinger, Dirk Schepmann, Gianluca Civenni, Erik Laurini, Domenico Marson, Carlo V. Catapano, Sabrina Pricl, and Bernhard Wünsch*

Table of Contents

1.	Purity data of all test compounds	S2
2.	Synthesis of starting compounds	S3
3.	Receptor binding studies	S5
4.	Computational details	S8
5.	Effect of σ_1 receptor ligand **4a** on proliferation and morphology of the human non-small cell lung cancer cell line A427	S11
6.	Growth inhibition of DU145 tumor cells	S16
7.	References	S18
8.	^{1}H and ^{13}C NMR spectra of the synthesized compounds	S20

1. Purity data of all test compounds

compod.	purity by HPLC
**4a**	99.0 %
**4b**	97.8 %
**13a**	96.3 %
**13b**	87.7 %
**18a**	95.6 %
**18b**	96.1 %
**19a**	94.2 %
**19b**	98.3 %
**20a**	96.6 %
**21a**	96.9 %
**22a**	99.7 %

2. Synthesis of starting compounds

2.1. 1-Tosylpiperidin-4-one (6a)[1]

Piperidin-4-one **5** (5.0 g, 32.5 mmol) and K_2CO_3 (15.7 g, 1144 mmol, 3.5 eq) were suspended in CH_3CN (30 mL) and the mixture was cooled to 0 °C with an ice-bath. *p*TsCl (6.82 g, 35.8 mmol, 1.1 eq) dissolved in CH_3CN (70 mL) was added to the suspension, the ice bath was removed and the reaction mixture was stirred for 18 h at rt. Then 1 M aq. HCl was added and the mixture was extracted with ethyl acetate (3 x 100 mL). The combined organic layers were washed with $NaHCO_3$ (50 mL) and brine (50 mL), dried (Na_2SO_4), filtered and concentrated *in vacuo*. The crude product was purified by automated fc (Snap, 340 g, V = 880 mL, cyclohexane:ethyl acetate = 2:1, R_f = 0.33). Colorless solid, mp 130 °C, yield 7.78 g (95 %). $C_{12}H_{15}NO_3S$ (253.3 g/mol). HR-MS (APCI): m/z = 254.0863 (calcd. 254.0845 for $C_{12}H_{16}NO_3S$ $[M+H]^+$). ^1H NMR (400 MHz, $CDCl_3$): δ (ppm) = 2.44 (s, 3H, CH_3), 2.53 (t, J = 6.2 Hz, 4H, 3-CH_2, 5-CH_2), 3.38 (t, J = 6.2 Hz, 4H, 2-CH_2, 6-CH_2), 7.34 (d, J = 8.2 Hz, 2H, 3-H_{Tos}, 5-H_{Tos}), 7.68 (d, J = 8.2 Hz, 2H, 2-H_{Tos}, 6-H_{Tos}). ^{13}C NMR (151 MHz, $CDCl_3$): δ (ppm) = 21.7 (CH_3), 40.8 (C-3, C-5), 46.0 (2C, C-2, C-6), 127.7 (2C, C-2$_{Tos}$, C-6$_{Tos}$), 130.1 (2C, C-3$_{Tos}$, C-5$_{Tos}$), 133.5 (C-1$_{Tos}$), 144.3 (C-4$_{Tos}$), 205.8 (C-4). FT-IR (neat): ν [cm^{-1}] = 1716 (C=O), 1157 (SO_2N). Purity (HPLC): 99.6 %, t_R = 16.4 min.

2.2. 1-Tosyl-2,3-dihydropyridin-4(1*H*)-one (7a)[1]

Iodoxybenzoic acid (IBX with 20% benzoic acid as stabilizer, 5.09 g, 14.5 mmol, 1.5 eq) and 4-methylmorpholin-4-oxide (NMO), (3.79 g, 32.4 mmol, 3.0 eq) were dissolved in DMSO (15 mL) and the piperidone **6a** (2.73 g, 10.8 mmol) dissolved in DMSO (30 mL) was added to the solution. The mixture was stirred for 72 h at 30 °C in the dark. The reaction mixture was poured into a saturated solution of $NaHCO_3$ (50 mL), the mixture was extracted with Et_2O (3 x 50 mL) and the combined Et_2O layers were washed with $NaHCO_3$, brine and water. The organic layer was dried (Na_2SO_4) and concentrated in vacuo. The crude product was purified by automated fc (Snap, 340 g, V = 750 mL, CH_2Cl_2:ethyl acetate = 9:1, R_f = 0.51). Colorless solid, mp 109 °C, yield 2.08 g (77 %) $C_{12}H_{13}NO_3S$ (251.3 g/mol). HR-MS (APCI): m/z =252.0720 (calcd. 252.0689 for $C_{12}H_{14}NO_3S$ $[M+H]^+$) ^1H NMR (600 MHz, $CDCl_3$): δ (ppm) = 2.46 (s, 3H, CH_3), 2.52 (t,

J = 7.7 Hz, 2H, 3-CH_2), 3.71, (t, *J* = 7.7 Hz, 2H, 2-CH_2), 5.37 (d, *J* = 8.4 Hz, 1H, 5-CH), 7.35 – 7.40 (m, 2H, 3-H_{Tos}, 5-H_{Tos}), 7.68 – 7.72 (m, 3H, 6-CH, 2-H_{Tos}, 6-H_{Tos}). ^{13}C NMR (151 MHz, $CDCl_3$): δ (ppm) = 21.8 (CH_3), 35.6 (C-3), 44.1 (C-2), 108.3 (C-5), 127.4 (2C, C-2_{tosyl}, C-6_{tosyl}), 130.5 (2C, C-3_{tosyl}, C-5_{tosyl}), 133.9 (C-1_{tosyl}), 143.7 (C-6), 145.5 (C-4_{tosyl}), 191.8 (C-4). FT-IR (neat): ν [cm^{-1}] = 1665 (C=O), 1594 (C=C), 1124 (SO_2N). Purity (HPLC): 94.0 %, t_R = 17.7 min.

3. Receptor binding studies

3.1. Materials

Guinea pig brains, rat brains and rat livers were commercially available from Harlan-Winkelmann, Borchen, Germany. Pig brains were a donation of the local slaughterhouse (Coesfeld, Germany). The mouse recombinant L(tk) cells stably expressing the GluN2B subunit-containing NMDAR were obtained from Prof. Dr. Dieter Steinhilber (Frankfurt, Germany). Homogenizers: Elvehjem Potter (B. Braun Biotech International, Melsungen, Germany) and Soniprep® 150, MSE, London, UK). Centrifuges: Cooling centrifuge model Rotina® 35R (Hettich, Tuttlingen, Germany) and High-speed cooling centrifuge model Sorvall® RC-5C plus (Thermo Fisher Scientific, Langenselbold, Germany). Multiplates: standard 96-well multiplates (Diagonal, Münster, Germany). Shaker: self-made device with adjustable temperature and tumbling speed (scientific workshop of the institute). Harvester: MicroBeta® FilterMate 96 Harvester. Filter: Printed Filtermat Typ A and B. Scintillator: Meltilex® (Typ A or B) solid state scintillator. Scintillation analyzer: MicroBeta® Trilux (all Perkin Elmer LAS, Rodgau-Jügesheim, Germany).

3.2. Preparation of membrane homogenates from guinea pig brain

Five guinea pig brains were homogenized with the potter (500-800 rpm, 10 up and down strokes) in 6 volumes of cold 0.32 M sucrose. The suspension was centrifuged at 1,200 x g for 10 min at 4 °C. The supernatant was separated and centrifuged at 23,500 x g for 20 min at 4 °C. The pellet was resuspended in 5-6 volumes of buffer (50 mM TRIS, pH 7.4) and centrifuged again at 23,500 x g (20 min, 4 °C). This procedure was repeated twice. The final pellet was resuspended in 5-6 volumes of buffer and frozen (-80 °C) in 1.5 mL portions containing about 1.5 mg protein/mL.

3.3. Preparation of membrane homogenates from rat liver

Two rat livers were cut into small pieces and homogenized with the potter (500-800 rpm, 10 up and down strokes) in 6 volumes of cold 0.32 M sucrose. The suspension was centrifuged at 1,200 x g for 10 min at 4 °C. The supernatant was separated and centrifuged at 31,000 x g for 20 min at 4 °C. The pellet was resuspended in 5-6 volumes of buffer (50 mM TRIS, pH 8.0) and incubated at rt for 30 min. After the incubation, the

suspension was centrifuged again at 31,000 x g for 20 min at 4 °C. The final pellet was resuspended in 5-6 volumes of buffer and stored at -80 °C in 1.5 mL portions containing about 2 mg protein/mL.

3.4. Determination of protein concentration

The protein concentration was determined by the method of Bradford,[2] modified by Stoscheck.[3] The Bradford solution was prepared by dissolving 5 mg of Coomassie Brilliant Blue G 250 in 2.5 mL of EtOH (95 %, v/v). 10 mL deionized H_2O and 5 mL phosphoric acid (85 %, m/v) were added to this solution, the mixture was stirred and filled to a total volume of 50 mL with deionized water. The calibration was carried out using bovine serum albumin as a standard in 9 concentrations (0.1, 0.2, 0.4, 0.6, 0.8, 1.0, 1.5, 2.0 and 4.0 mg /mL). In a 96 well standard multiplate, 10 µL of the calibration solution or 10 µL of the membrane receptor preparation were mixed with 190 µL of the Bradford solution, respectively. After 5 min, the UV absorption of the protein-dye complex at λ = 595 nm was measured with a plate reader (Tecan Genios®, Tecan, Crailsheim, Germany).

3.5. General procedures for the binding assays

The test compound solutions were prepared from the 10 mM stock solution. To obtain the required test solutions for the assay, the DMSO stock solution was diluted with the respective assay buffer. The filtermats were presoaked in 0.5 % aqueous polyethyleneimine solution for 2 h at room temperature before use. All binding experiments were carried out in duplicates in the 96-well multiplates. Generally, the assays were performed by addition of 50 µL of the respective assay buffer, 50 µL of test compound solution in various concentrations (10^{-5}, 10^{-6}, 10^{-7}, 10^{-8}, 10^{-9} and 10^{-10} mol/L), 50 µL of the corresponding radioligand solution and 50 µL of the respective receptor preparation into each well of the multiplate (total volume 200 µL). The receptor preparation was always added last. During the incubation, the multiplates were shaken at a speed of 500-600 rpm at the specified temperature. Unless otherwise noted, the assays were terminated after 120 min by rapid filtration using the harvester. During the filtration, each well was washed five times with 300 µL of water. Subsequently, the

filtermats were dried at 95 °C. The solid scintillator was melted on the dried filtermats at a temperature of 95 °C for 5 min. After solidifying of the scintillator at room temperature, the trapped radioactivity in the filtermats was measured with the scintillation analyzer. Each position on the filtermat corresponding to one well of the multiplate was measured for 5 min with the [^{3}H]-counting protocol. The overall counting efficiency was 20 %.[3] The IC_{50} values were calculated with the program GraphPad Prism® 3.0 (GraphPad Software, San Diego, CA, USA) by non-linear regression analysis. Subsequently, the IC_{50} values were transformed into K_i values using the equation of Cheng and Prusoff.[4] The K_i values are given as mean value ± SEM from three independent experiments.

3.6. σ_1 Receptor affinity

The assay was performed with the radioligand [^{3}H]-(+)-pentazocine (22.0 Ci/mmol; Perkin Elmer). The thawed membrane preparation of guinea pig brain cortex (about 100 µg of the protein) was incubated with various concentrations of test compounds, 2 nM [^{3}H]-(+)-pentazocine, and TRIS buffer (50 mM, pH 7.4) at 37 °C. The non-specific binding was determined with 10 µM unlabeled (+)-pentazocine. The K_d value of (+)-pentazocine is 2.9 nM.[5]

3.7. σ_2 Receptor affinity

The assays were performed with the radioligand [^{3}H]di-*o*-tolylguanidine (specific activity 50 Ci/mmol; ARC, St. Louis, MO, USA). The thawed rat liver membrane preparation (about 100 µg protein) was incubated with various concentrations of the test compound, 3 nM [^{3}H]di-*o*-tolylguanidine and buffer containing (+)-pentazocine (500 nM (+)-pentazocine in TRIS buffer (50 mM TRIS, pH 8.0)) at rt. The non-specific binding was determined with 10 µM non-labeled di-*o*-tolylguanidine. The K_d value of di-*o*-tolylguanidine is 17.9 nM.[6]

4. Computational details

The molecular structures of σ_1 receptor was obtained starting from the available Protein Data Bank file (pdb code: 5HK1)[7] and following a procedure previously described.[8-10] The optimized structure of the new piperidine derivatives was docked into the identified binding pocket using Autodock 4.2.6/Autodock Tools 1.4.6[11] on a win64 platform. The resulting docked conformations were clustered and visualized; then, the structure of each resulting complex characterized by the lowest Autodock interaction energy in the prevailing cluster was selected for further modelling. Each compound/σ_1 complex obtained from the docking procedure was refined in Amber 20[12] following a well validated procedure.[8-10] Briefly, the system density and volume were relaxed in NPT ensemble maintaining the Berendsen barostat for 20 ns. After this step, 50 ns of unrestrained NVT production simulation was run for each system. The free energy of binding (ΔG_{bind}) between each compound and the σ_1 receptor was estimated by resorting to the well validated Molecular Mechanics/Poisson-Boltzmann Surface Area (MM/PBSA) approach[13] implemented in Amber 20. The per residue binding free energy decomposition (PRBFED) was carried out using the Molecular Mechanics/Generalized Boltzmann Surface Area (MM/GBSA) approach,[14] and was based on the same snapshots used in the binding free energy calculation. Molecular graphics images were produced using the UCSF Chimera package (v.1.15).[15] Chimera is developed by the Resource for Biocomputing, Visualization, and Informatics at the University of California, San Francisco (supported by NIGMS P41-GM103311). All other graphs were obtained using GraphPad Prism (v. 8.0).[16]

Supplementary Tables

Table S1: In silico binding thermodynamics for the cyclohexane derivate **3** and the new piperidine derivatives **4a**, **20a**, **21a** and **22a** towards σ_1 receptor. Errors on ΔG_{bind}, ΔH_{bind}, and $T\Delta S_{bind}$ values are within 5%.

compd.	ΔG_{bind} (kcal/mol)	ΔH_{bind} (kcal/mol)	$-T\Delta S_{bind}$ (kcal/mol)
**3**	-11.31	-20.69	9.38
**4a**	-9.48	-18.31	8.83
**20a**	-10.06	-19.33	9.27
**21a**	-10.12	-19.18	9.06
**22a**	-9.97	-18.93	8.96

Table S2: Per-residue binding enthalpy decomposition (ΔH_{res}) for the cyclohexane derivate **3** and the new piperidine derivatives **4a**, **20a**, **21a** and **22a** towards σ_1 receptor. Only those amino acids critical for the binding are listed. Errors on ΔH_{res} values are within 5%.

compd.	ΔH_{res} (kcal/mol)				
	E172, Y103	F107	I124	L105, T181, A185	L182, L186, T202, Y206
**3**	-5.69	-1.27	-1.11	-3.03	-3.57
**4a**	-4.93	-1.23	-1.18	-2.23	-2.68
**20a**	-5.11	-1.18	-1.07	-2.34	-3.09
**21a**	-5.06	-1.16	-1.19	-2.38	-3.16
**22a**	-5.09	-1.19	-1.15	-2.42	-2.95

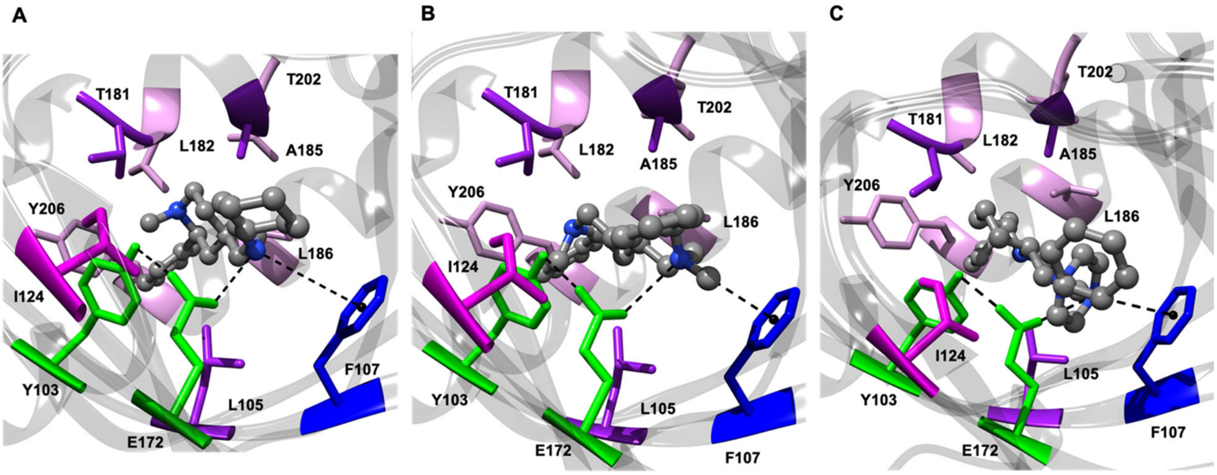

Figure S1: Details of compounds **20a** (**A**), **21a** (**B**) and **22a** (**C**), in the binding pocket of the σ_1 receptor. Compounds are shown as atom-colored sticks-and-balls (C, grey, N, blue, O, red) while the side chains of the σ_1 protein residues are depicted as colored sticks and labelled. Hydrogen atoms, water molecules, ions, and counterions are omitted for clarity.

5. Effect of σ_1 receptor ligand 4a on proliferation and morphology of the human non-small cell lung cancer cell line A427

4.1. Materials

The A427 cells were kindly provided by Prof. Dr. Joachim Jose (Münster, Germany). Medium: RPMI 1640 w/ L-glutamine sterile filtered and phenol red indicator (Biowest, Nuaillé, France), FBS Superior (Biochrom AG, Berlin, Germany), penicillin/streptomycin (10,000 µg/mL) (Biochrom GmbH, Berlin, Germany). PBS Dulbecco w/o Ca^{2+}, w/o Mg^{2+}, low endotoxin and trypsin 0.25 % (w/v) in PBS w/o Ca^{2+}, w/o Mg^{2+} (both Biochrom GmbH, Berlin, Germany). Bovine serum albumin (VWR International GmbH, Darmstadt, Germany). Triton X 100 (Carl Roth GmbH & Co. KG, Karlsruhe, Germany). Biological safety cabinet: Thermo Scientific™ Safe 2020. Incubator: Heracell 150i CO2 Incubator (both Thermo Fisher Scientific Langenselbold, Germany). Autoclave: Systec DX-65 (Systec GmbH, Linden, Germany). Water bath: Julabo ED heating circulator with open bath (Julabo GmbH, Seelbach, Germany). Centrifuge: Centrifuge 5804 R (Eppendorf, Hamburg, Germany). Vortexer: VTX-3000L (LMS Co., Ltd., Tokyo, Japan). Cell counting: ScepterTM cell counter and ScepterTM 40 µm sensor (Merck KGaA, Darmstadt, Germany). Live Cell Imager: IncuCyte® S3 (Essen BioScience Ltd., Hertfordshire, UK). Plate reader: Mithras LB 940 (Berthold Technologies, Bad Wildbad, Germany).

5.2. Cultivation and subcultivation of A427 cells

For the preparation of the medium for the A427 cells 450 mL of RPMI 1640, 50 mL of FBS Superior and 5 mL of penicillin/streptomycin (10,000 µg/mL) were warmed to 37 °C in the water bath and mixed under sterile conditions in the biological safety cabinet. The content of one 2.0 mL cryovial (approx. 1 to 2 million cells) was added to 12 mL of the prepared medium in a 75 mL culture flask (VWR® Tissue Culture Flasks, VWR, Langenselbold, Germany). This flask was then stored in the incubator at 37 °C and a water saturated atmosphere with 5 % CO2. The unused medium was stored at 5 °C. The medium in the flask was changed when the indicator changed its color.

At a confluence of 80-90 %, the A427 cells of one flask were split over three flasks (VWR). The medium, PBS Dulbecco and trypsin were warmed to 37 °C in the water bath. The

medium was removed from the flask and the cells were rinsed with 12 mL of PBS. After the removal of the PBS, 4 mL of trypsin were added. The cells were incubated in the incubator at 37 °C for 5-10 min until approx. 80 % of the cells were detached from the surface. Then, 15 mL of medium were added. The mixture was centrifuged at 800 × g for 10 min at 4 °C. The supernatant was discarded and the pellet was resuspended in 12 mL of medium by manual vortexing. The medium with the cells was equally distributed over three culture flasks (VWR) and filled up with medium to a total volume of 12 mL. The cultivation conditions were the same as described above.

5.3. Live Cell imaging using the IncuCyte® S3 Live Cell Analysis System

The measurement of the cell confluence and the determination of the IC50 values were performed with the IncuCyte® S3 Live Cell Analysis System (Essen BioScience, Ltd., Royston, Hertfordshire, UK)

5.4. Preparation of 96-well plates

For the preparation of the 96-well plates for the Live Cell Imager, A427 cells from the flask were detached and centrifuged. The pellet was resuspended in 20 mL of the medium using the vortexer. 1 mL was used to determine the number of cells and the volume required for 8,000-12,000 cells was calculated. This volume was then added to each well of a sterile 96-well plate (Tissue Culture Testplates 96F 92696, TPP, Trasadingen, Switzerland) and the well was filled up to a total volume of 150 µL with medium.

At a confluence of 30-40 % the test compounds were added. Dilutions of concentrations of 200, 100, 40, 20, 10 and 4 µM were prepared in medium with 2 % DMSO. 50 µL of each dilution were added to the wells, resulting in a final assay concentration of 50, 25, 10, 5, 2.5 and 1 µM, respectively. Each concentration was measured in triplicates on each plate and on three different days.

5.5. Measurement of cell confluence

The cell confluence was measured using the IncuCyte® S3. For the scanning with the company’s own program (IncuCyte S3 2017A), the HD phase imaging mode was chosen

at a 10-fold magnification. Four pictures were taken from each well every 2 h until the growth curve of the cells reached a plateau (approx. 5 d). The mask for the analysis of the confluence in the pictures had a segmentation adjustment of 1.1, a cleanup adjustment size of -1 pixel and an area filter of minimum 200 µm2. After the analysis, a graph showing the development of the confluence over time was produced.

5.6. Competitive confluence assay

The plates were prepared as described above with the exception that the cells were grown in 100 µL instead of 150 µL of medium. After the cell confluence reached 30-40 %, the cells were treated with the respective test compounds and the agonist (+)-pentazocine (final concentration: 10 µM).

5.7. Calculation of IC_{50} values

The cell confluence (in %), was exported to Microsoft Excel (Microsoft Corporation, Redmond, USA) to calculate the inhibition of the cell growth. The last time point from the measurement of each concentration was taken as the maximal confluence was then reached. The calculation was done as described in equation 1.

Eq. 1: $inhibition\ (\%) = 100 * (1 - \frac{confluence\ (under\ treatment)[\%]}{confluence\ (without\ treatment)[\%]})$

The inhibition value was then transferred to Origin 2018b (OriginLab, Northampton, USA) to generate a graph showing the inhibition in dependence of the common logarithm of the concentration. First, a scatter diagram was generated. The fitting of the curve was done by non-linear, sigmoidal, dose/response regression. The non-linear regression was calculated with equation 2.

Eq. 2: $y = A1 + \frac{A2-A1}{1+10^{(\log x_0 - x)*p}}$

with y being the inhibition in %, A1 being the horizontal approach to the lowest value in inhibition (bottom (left) asymptote), A2 being the horizontal approach to the highest value

in inhibition (top (right) asymptote), A1<A2, logx0 being the center of the curve, x being the common logarithm of the concentration and p being the hill slope. At an inhibition of 50 % the x value is equivalent to the log10(IC50) which then had to be transformed to the IC50 value. From the obtained values, the mean value and the corresponding standard deviation (SD) were calculated.

5.8. Results

Tumor cells with high σ_1 receptor expression, such as the non-small cell lung cancer cell line A427,[17] show reduced cell proliferation after treatment with σ_1 receptor ligands. To determine the effects of the moderate σ_1 receptor ligand **4a** on this tumor cell line, A427 cells were incubated with the piperidine **4a** and the cells were observed with the Live Cell Imager IncuCyte®. IncuCyte® allows the continuous observation of the morphology, behavior and growth of cells over a long period of time without interruption of the incubation process. **4a** was selected due to its high lipophilic ligand efficiency (see Section 2.4.) and good water solubility at higher concentrations.

Table S3: Inhibition of cell proliferation by **4a** and two σ_1 receptor reference compounds. The growth curve of the cells reached a plateau after approx. 5 d. The last time point from the measurement of each concentration was taken to calculate the IC_{50} values (n = 3).

compd.	σ_1 affinity K_i ± SEM [nM]	Inhibition of cell confluency IC_{50} ± SD [µM]	Inhibition of cell confluency in presence of (+)-pentazocine (10 µM); IC_{50} ± SD [µM][a)]
**4a**	165	17 ± 2	30 ± 4
haloperidol	6.6 ± 0.9	16 ± 3	24 ± 2
(+)-pentazocine	5.4 ± 0.5	no effect	-

A strong inhibition of the A427 cell proliferation was observed for **4a** resulting in an IC_{50} value of 17 µM (Table S3). The σ_1 antagonist haloperidol showed a reduction of cell proliferation in the same concentration range (IC_{50} = 16 µM), whereas the selective σ_1 agonist (+)pentazocine did not affect A427 cell proliferation. In order to prove that the inhibition of cell growth was mediated by the σ_1 receptor, A427 cells were co-incubated

with **4a** and (+)-pentazocine (10 µM) or haloperidol and (+)-pentazocine (10 µM), respectively. In the presence of the agonist (+)-pentazocine, the inhibitory effects of **4a** and haloperidol on cell proliferation were considerably reduced. The IC_{50} value of **4a** and haloperidol was shifted from 18 µM to 30 µM and from 16 µM to 24 µM, respectively.

Altogether, the observed *in vitro* effects of **4a** on A427 tumor cells are comparable with those of the σ_1 antagonist haloperidol. Moreover, they can be reduced by co-incubation of a σ_1 receptor agonist. Therefore, it can be concluded that **4a** behaves as σ_1 receptor antagonist in this A427 tumor cell proliferation assay.

6. Growth inhibition of DU145 tumor cells

DU145 cells were purchased from ATCC and maintained in adherent conditions in RPMI 1640 medium (21875-034, Gibco®) supplemented with 2 % (*S*)-glutamine, 10 % fetal bovine serum (FBS) (10437-036, Gibco®) and 1 % penicillin-streptomycin (15140-122, Gibco®). For the proliferation assays, DU145 cells were seeded at a density of 2 x 10^3 cells/well in 96-well tissue culture plates. 24 h later, the cells were treated with the compounds (2.5-10 µM) for 72 h. Then, the cells were fixed with 10 % (w/v) trichloroacetic acid and stained with sulforhodamine B for 30 min. The excess of the dye was removed by washing repeatedly with 1 % (v/v) acetic acid and the protein-bound dye was solubilized with Tris base solution for optical density (OD) measurement at 510 nm with a microplate reader. Cell proliferation and viability were expressed as percentages relative to those of DMSO treated cells. The data represent the results from three independent experiments. Statistical analysis was done using T-test and Anova. Figure S2 shows the growth inhibition of the DU145 cell line by three piperidines (**20a**, **21a**, **22a**) and two reference σ_1 receptor antagonists.

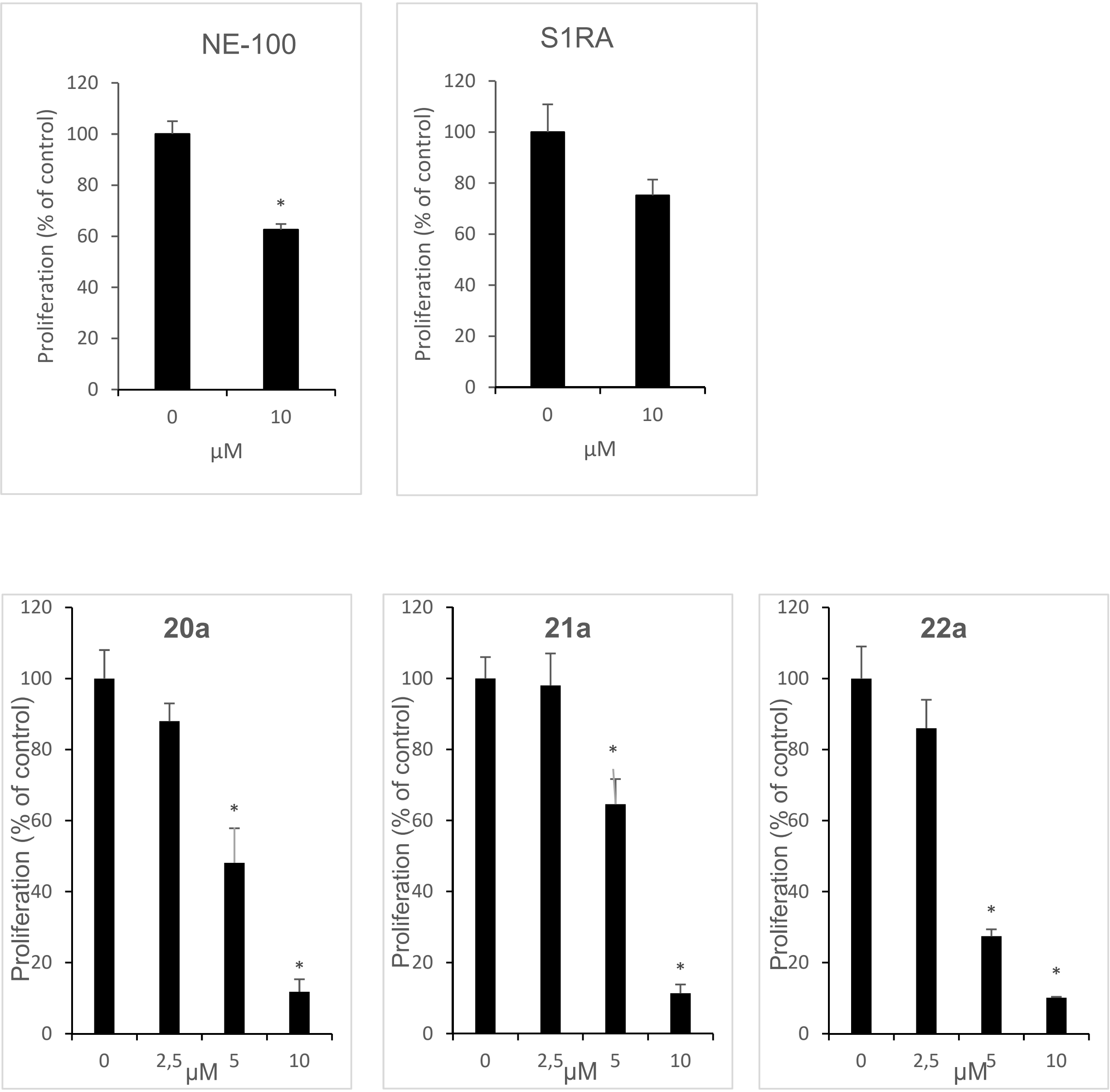

Figure S2: Inhibition of the growth of the androgen negative human prostate tumor cell line DU145 by the piperidines **20a**, **21a** and **22a** (bottom panels) and the reference σ_1 antagonists NE100 and S1RA (top panels). Data are mean ± SD (n = 3). *$p \leq 0.05$ vs control.

7. References

1. R. Sebesta, M.G. Pizzuti, A.J. Boersma, A.J. Minnaard, B.L. Feringa, Catalytic enantioselective conjugate addition of dialkylzinc reagents to N-substituted-2,3-dehydro-4-piperidones, Chem. Commun. (2005) 1711–1713.
2. M. M. A. Bradford, Rapid and sensitive Method for the Quantitation of Microgram Quantities of Protein Utilizing the Principle of Protein-Dye Binding, Anal. Biochem. *72* (1976) 248–254.
3. C. Stoscheck, Quantification of Protein, Method. Enzymol. 182 (1990) 50–68.
4. Y. C. Cheng, W. H. Prusoff, Relationship between the inhibition constant (KI) and the concentration of inhibitor which causes 50 per cent inhibition (I50) of an enzymatic reaction, Biochem. Pharmacol. 22 (1973) 3099–3108.
5. D. L. DeHaven-Hudkins, L. C. Fleissner, F. Y. Ford-Rice, Characterization of the binding of [3H]-(+)-pentazocine to σ recognition sites in guinea pig brain, Eur. J. Pharm-Molec. Ph. 227 (1992) 371–378.
6. R. H. Mach, C. R. Smith, S. R. Childers, Ibogaine possesses a selective affinity for σ2 receptors, Life Sci. 57 (1995) PL57–PL62.
7. H. R. Schmidt, S. D. Zheng, E. Gurpinar, A. Koehl, A. Manglik, A. C. Kruse, Crystal structure of the human sigma 1 receptor, Nature 532(7600) (2016) 527–530.
8. E. Kronenberg, F. Weber, S. Brune, D. Schepmann, C. Almansa, K. Friedland, E. Laurini, S. Pricl, B. Wünsch, Synthesis and Structure−Affinity Relationships of Spirocyclic Benzopyrans with Exocyclic Amino Moiety, J. Med. Chem. 62 (2019) 4204–4217.
9. N. Kopp, G. Civenni, D. Marson, E. Laurini, S. Pricl, C. V. Catapano, H.-U. Humpf, C. Almansa, F. R. Nieto, D. Schepmann, B. Wünsch, Chemoenzymatic synthesis of 2,6-disubsttuted tetrahydropyrans with high σ_1 receptor affinity, antitumor and analgesic activity. Eur. J. Med. Chem. 219 (2021) 113443.
10. N. Kopp, C. Holtschulte, F. Börgel, K. Lehmkuhl, K. Friedland, G. Civenni, E. Laurini, C. V. Catapano, S. Pricl, H.-U. Humpf, D. Schepmann, B. Wünsch, Novel σ_1 antagonists designed for tumor therapy: structure - activity relationships of aminoethyl substituted cyclohexanes, Eur. J. Med. Chem. 210 (2021) 112950.

11. G. M. Morris, R. Huey, W. Lindstrom, M. F. Sanner, R. K. Belew, D. S. Goodsell, A. J. Olson, AutoDock4 and AutoDockTools4: Automated docking with selective receptor flexibility. J. Comput. Chem. 30 (2009) 2785-2791.
12. D.A. Case, H.M. Aktulga, K. Belfon, I.Y. Ben-Shalom, S.R. Brozell, D.S. Cerutti, T.E. Cheatham, III, G.A. Cisneros, V.W.D. Cruzeiro, T.A. Darden, R.E. Duke, G. Giambasu, M.K. Gilson, H. Gohlke, A.W. Goetz, R. Harris, S. Izadi, S.A. Izmailov, C. Jin, K. Kasavajhala, M.C. Kaymak, E. King, A. Kovalenko, T. Kurtzman, T.S. Lee, S. LeGrand, P. Li, C. Lin, J. Liu, T. Luchko, R. Luo, M. Machado, V. Man, M. Manathunga, K.M. Merz, Y. Miao, O. Mikhailovskii, G. Monard, H. Nguyen, K.A. O'Hearn, A. Onufriev, F. Pan, S. Pantano, R. Qi, A. Rahnamoun, D.R. Roe, A. Roitberg, C. Sagui, S. Schott-Verdugo, J. Shen, C.L. Simmerling, N.R. Skrynnikov, J. Smith, J. Swails, R.C. Walker, J. Wang, H. Wei, R.M. Wolf, X. Wu, Y. Xue, D.M. York, S. Zhao, and P.A. Kollman (2021), Amber 2021, University of California, San Francisco.
13. I. Massova,P. A. Kollman, Combined molecular mechanical and continuum solvent approach (MM-PBSA/GBSA) to predict ligand binding. Perspect. Drug Discov. 18 (2000) 113–135.
14. A. Onufriev, D. Bashford, D. A. Case, Modification of the generalized born model suitable for macromolecules. J. Phys. Chem. 104 (2000) 3712–3720.
15. E. F. Pettersen, T. D. Goddard, C. C. Huang, G. S. Couch, D. M. Greenblatt, E. C. Meng, T. E. Ferrin, UCSF Chimera--a visualization system for exploratory research and analysis, J. Comput. Chem. 25(13) (2004) 1605–1612.
16. GraphPad Prism version 6.00 for Windows, GraphPad Software, La Jolla California USA, www.graphpad.com.
17. C. Geiger, C. Zelenka, M. Weigl, R. Fröhlich, B. Wibbeling, K. Lehmkuhl, D. Schepmann, R. Grünert, P. J. Bednarski, B. Wünsch, Synthesis of bicyclic σ receptor ligands with cytotoxic activity, J. Med. Chem. 50 (2007) 6144 - 6153.

8. ^1H and ^{13}C NMR spectra

1-Tosylpiperidin-4-one (6a)

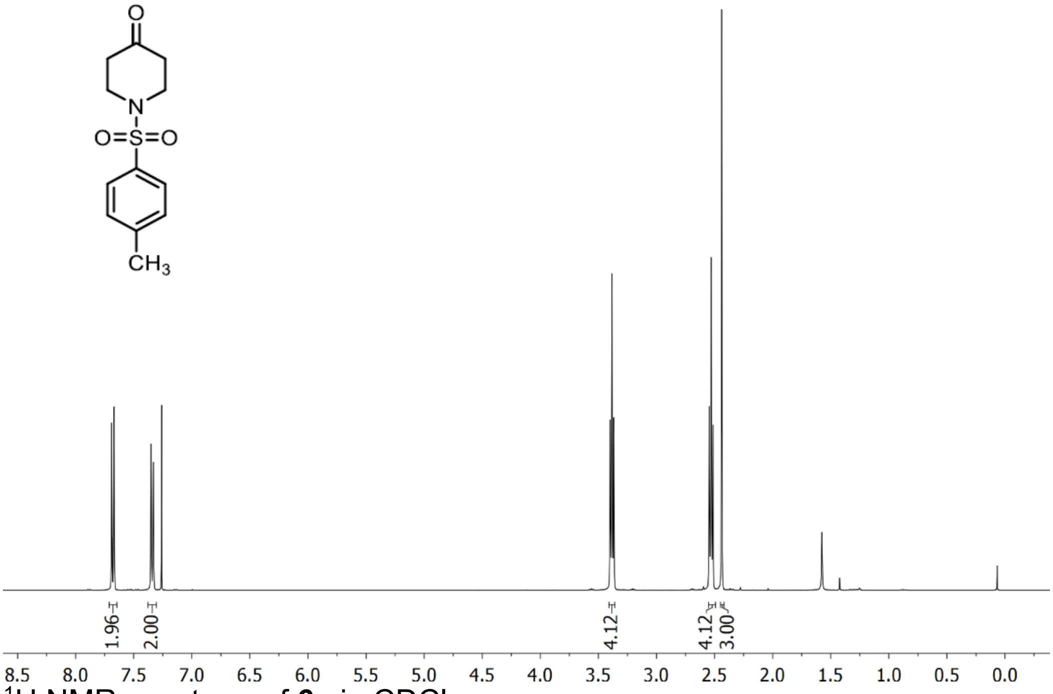

^1H NMR spectrum of **6a** in $CDCl_3$.

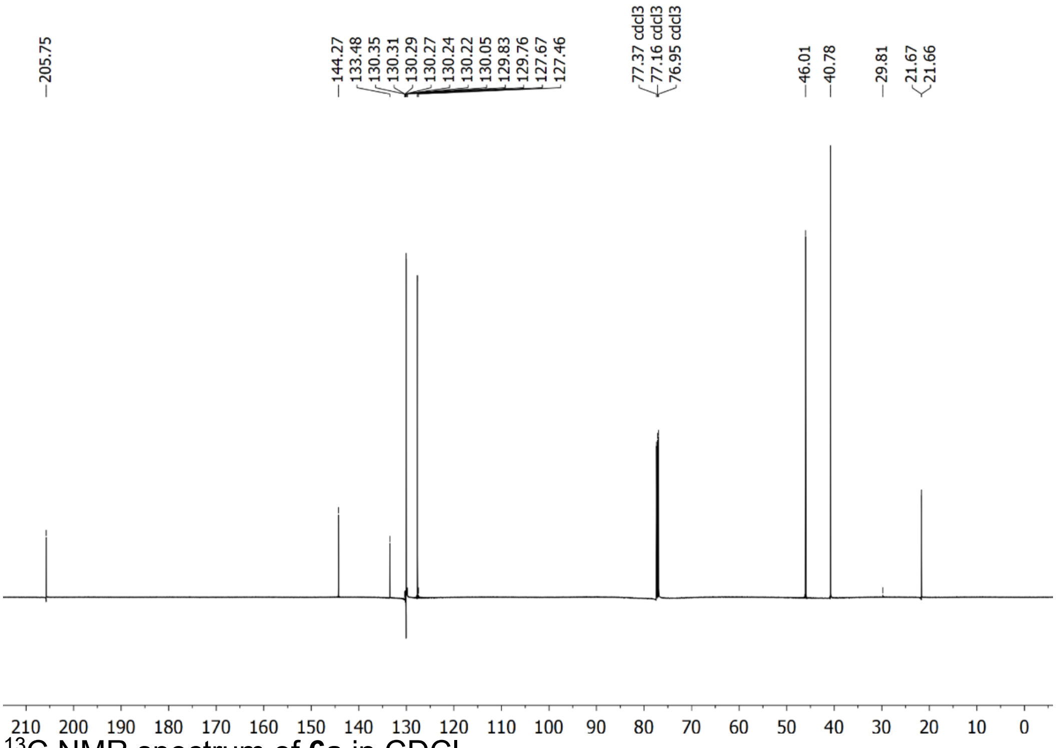

^{13}C NMR spectrum of **6a** in $CDCl_3$.

1-Tosyl-2,3-dihydropyridin-4(1*H*)-one (7a)

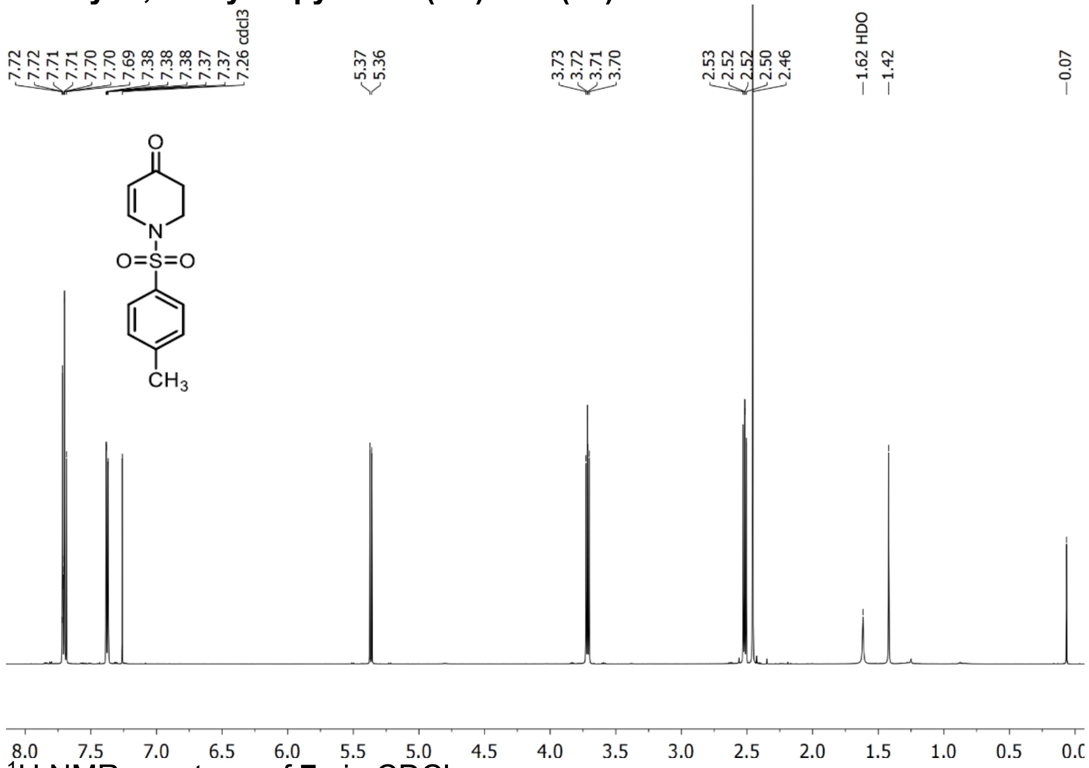

^{1}H NMR spectrum of **7a** in $CDCl_3$.

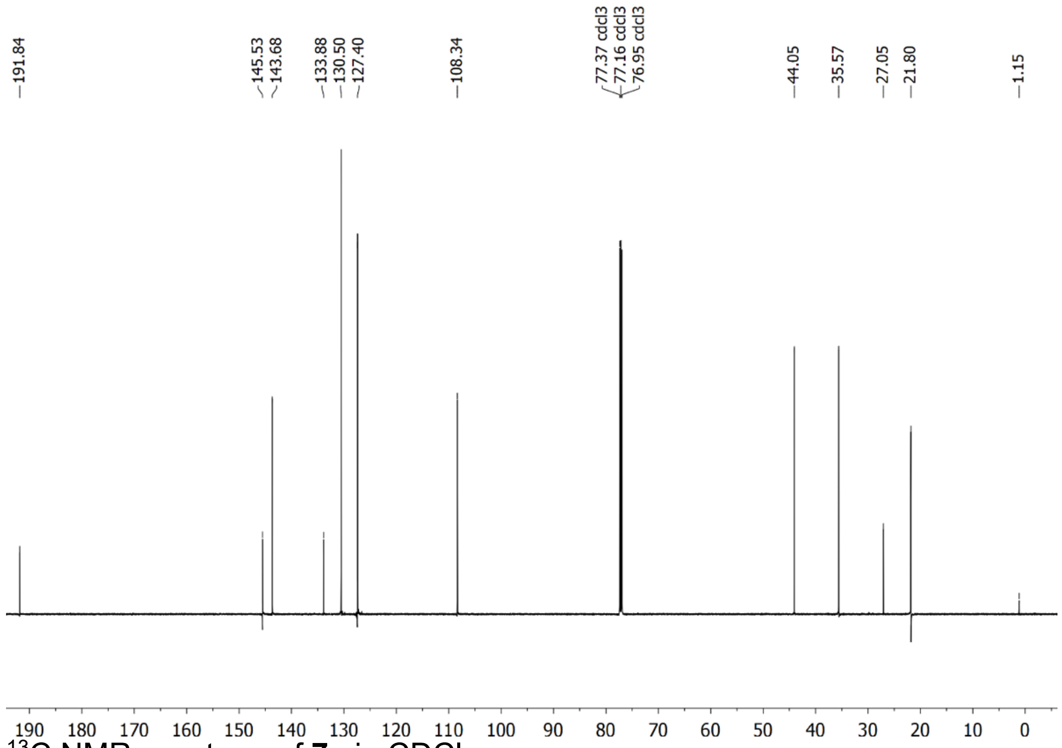

^{13}C NMR spectrum of **7a** in $CDCl_3$.

Benzyl 4-oxo-5,6-dihydropyridine-1(*2H*)-carboxylate (7b)

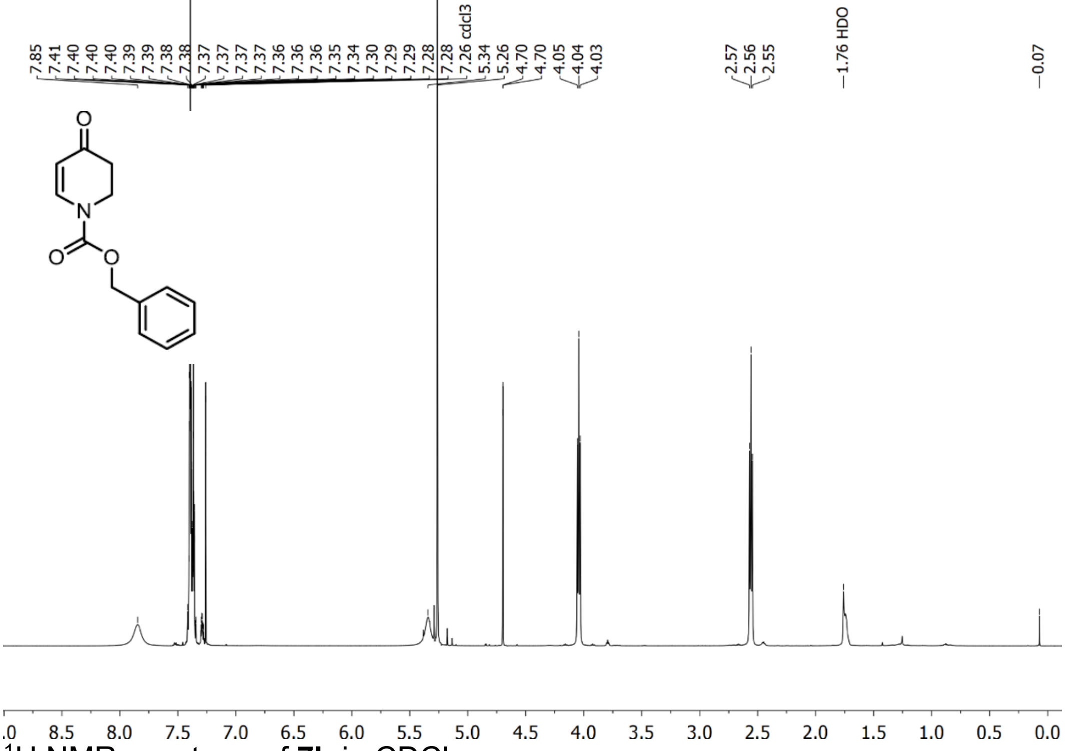

^{1}H NMR spectrum of **7b** in $CDCl_3$.

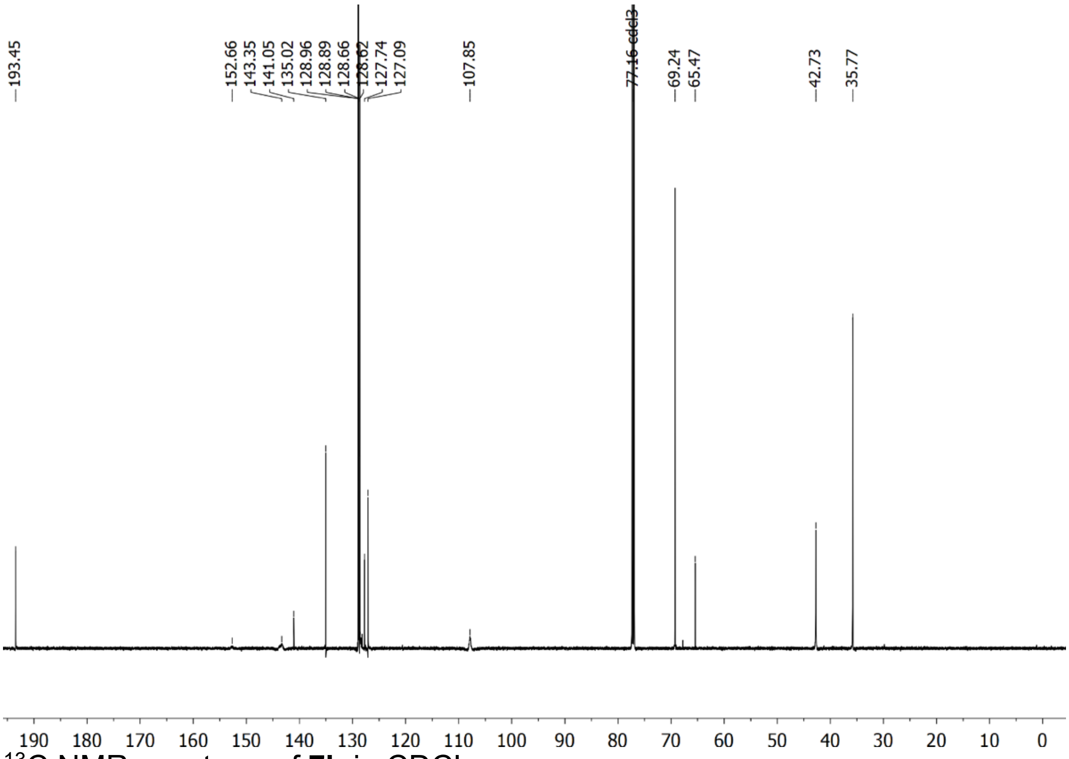

^{13}C NMR spectrum of **7b** in $CDCl_3$.

***tert*-Butyl 4-oxo-3,4-dihydropyridine-1(2*H*)-carboxylate (7c)**

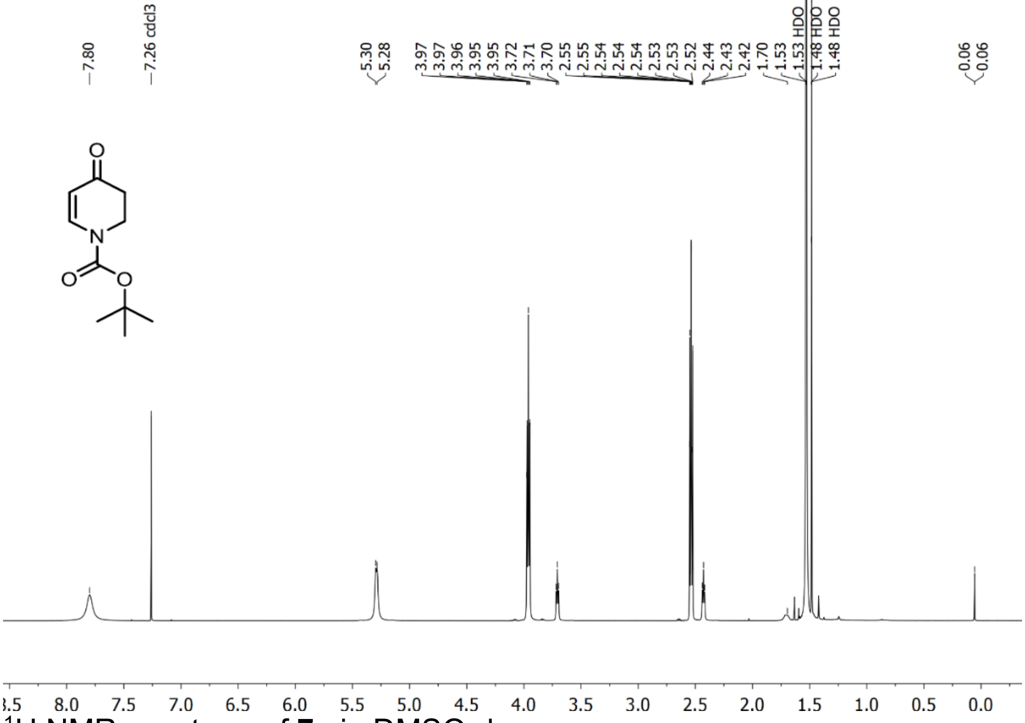

^{1}H NMR spectrum of **7c** in DMSO-d$_6$.

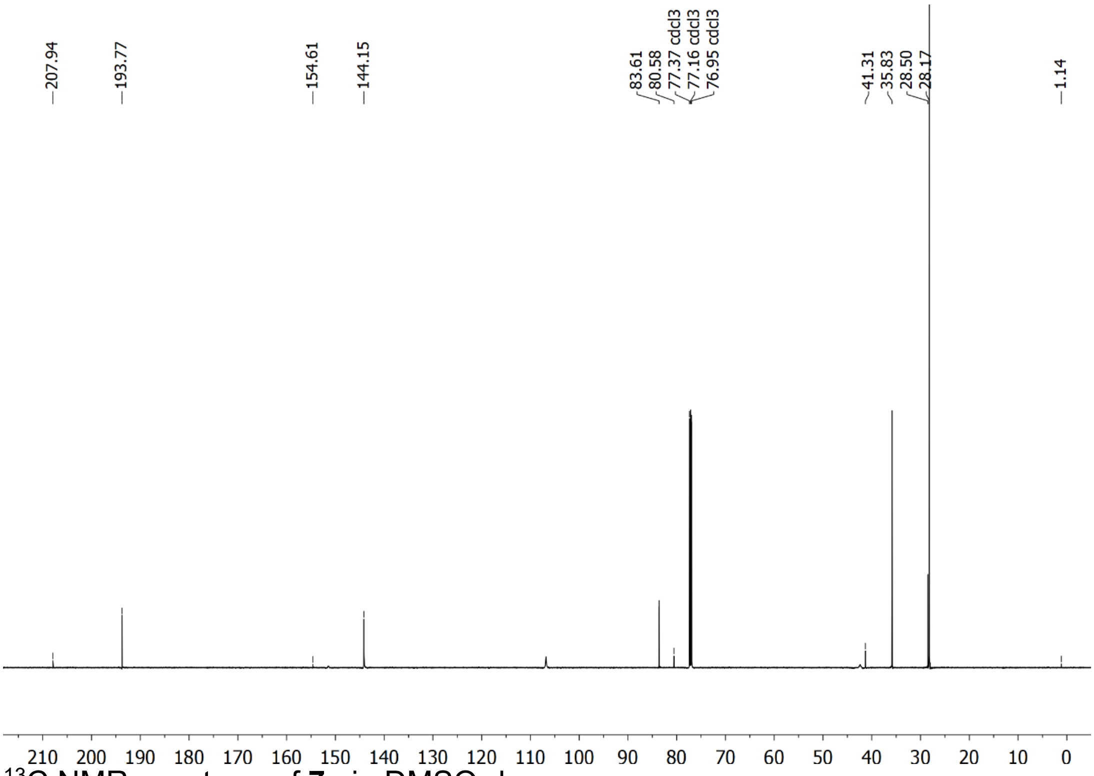

^{13}C NMR spectrum of **7c** in DMSO-d$_6$.

2-Phenyl-1-tosylpiperidin-4-one (8a)

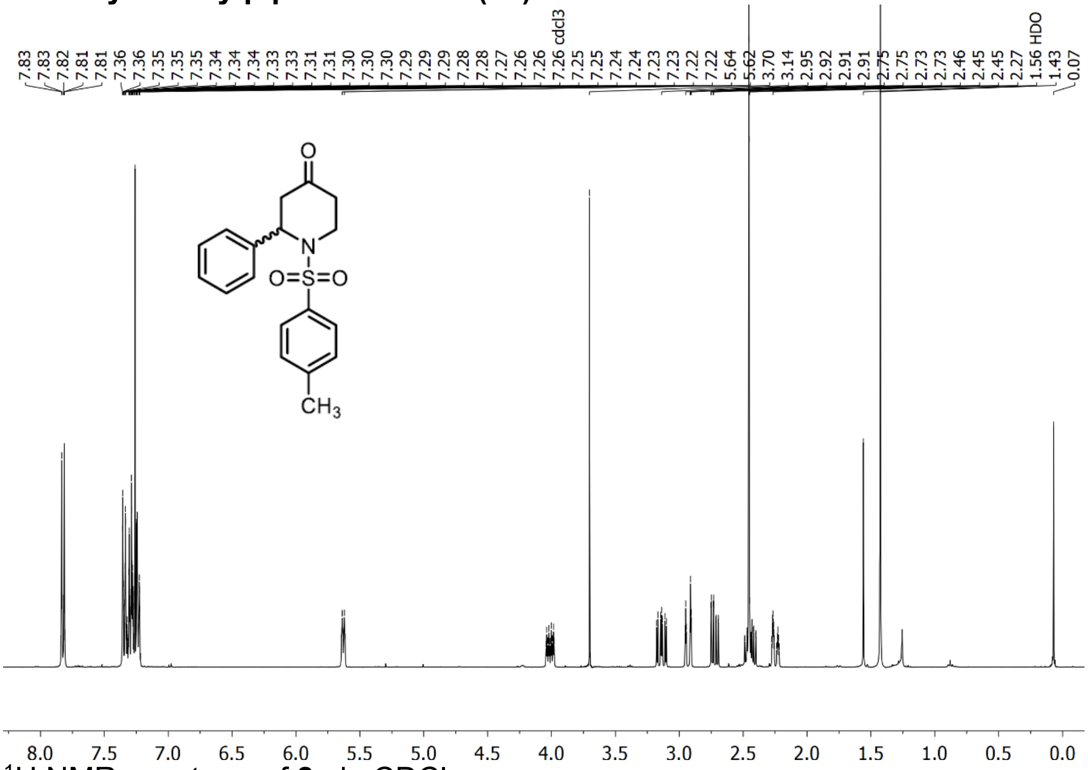

^{1}H NMR spectrum of **8a** in $CDCl_3$.

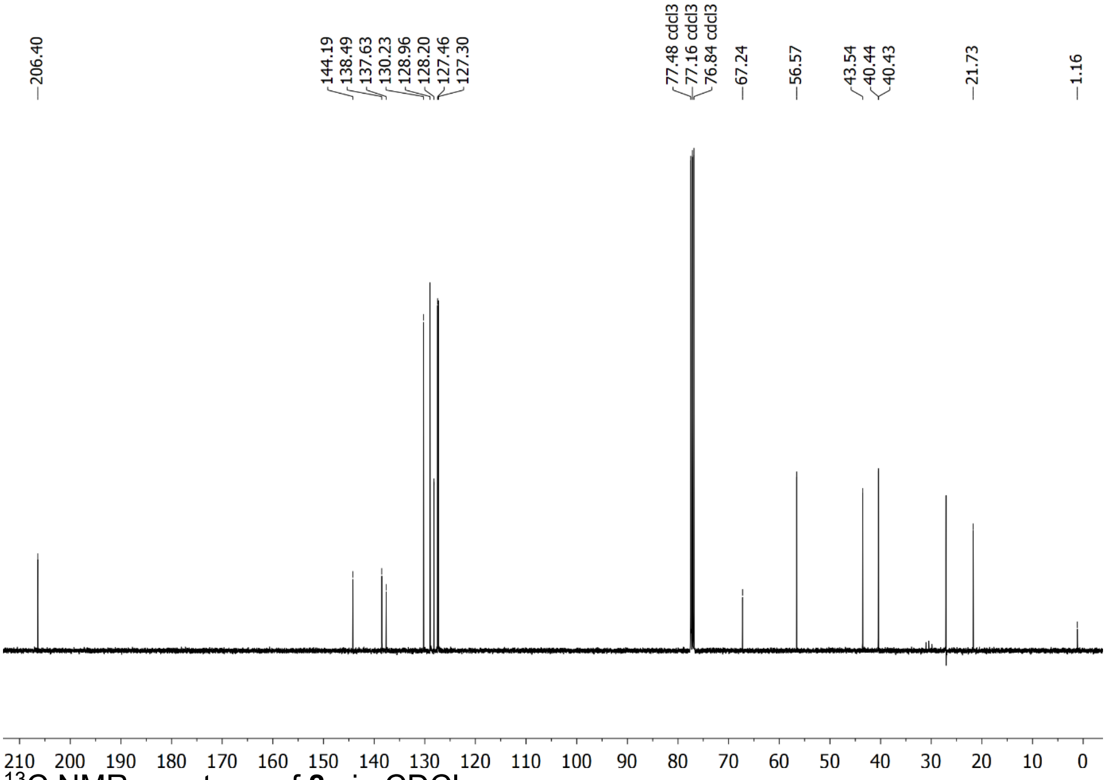

^{13}C NMR spectrum of **8a** in $CDCl_3$.

Benzyl 4-oxo-2-phenylpiperidine-1-carboxylate (8b)

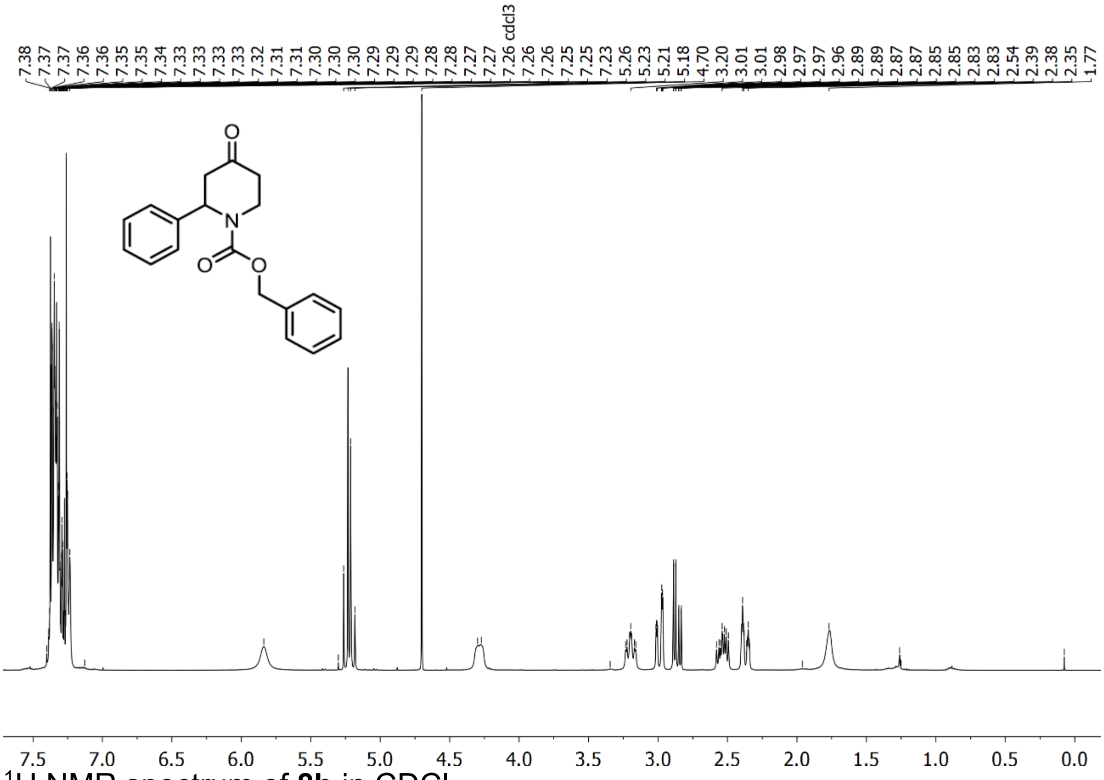

^{1}H NMR spectrum of **8b** in $CDCl_3$.

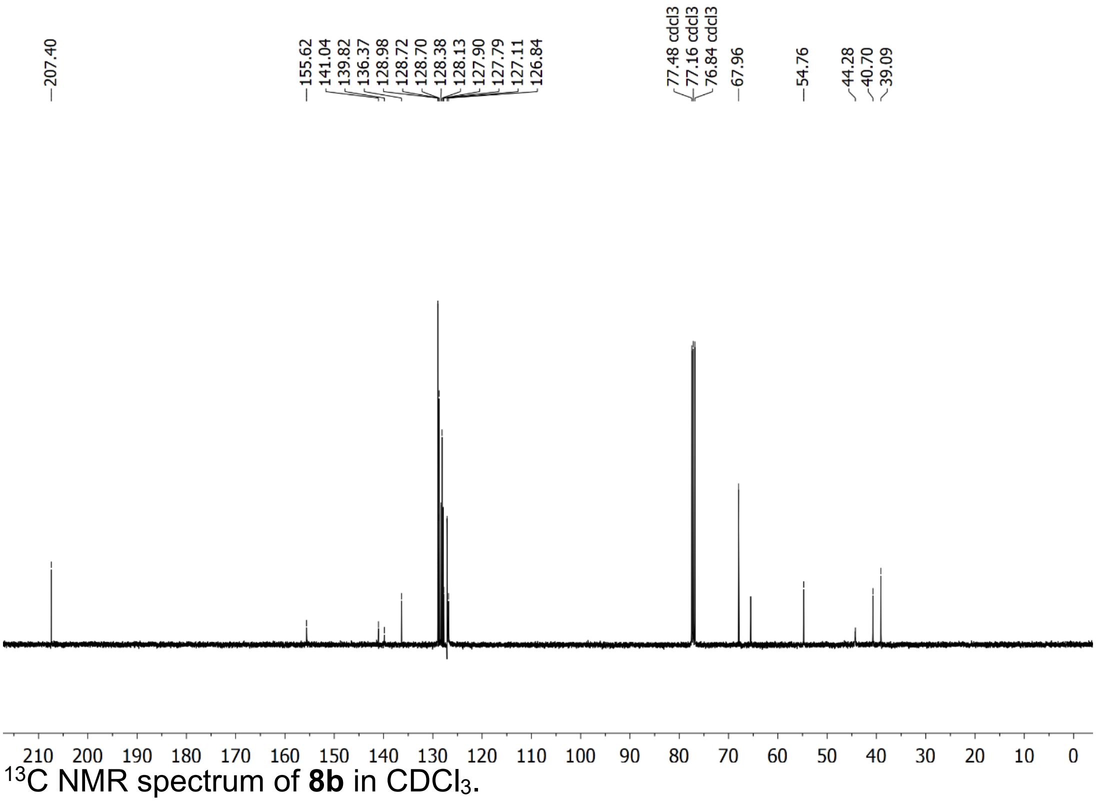

^{13}C NMR spectrum of **8b** in $CDCl_3$.

Ethyl (*E*)- and (*Z*)-2-(2-phenyl-1-tosylpiperidin-4-ylidene)acetate (9a)

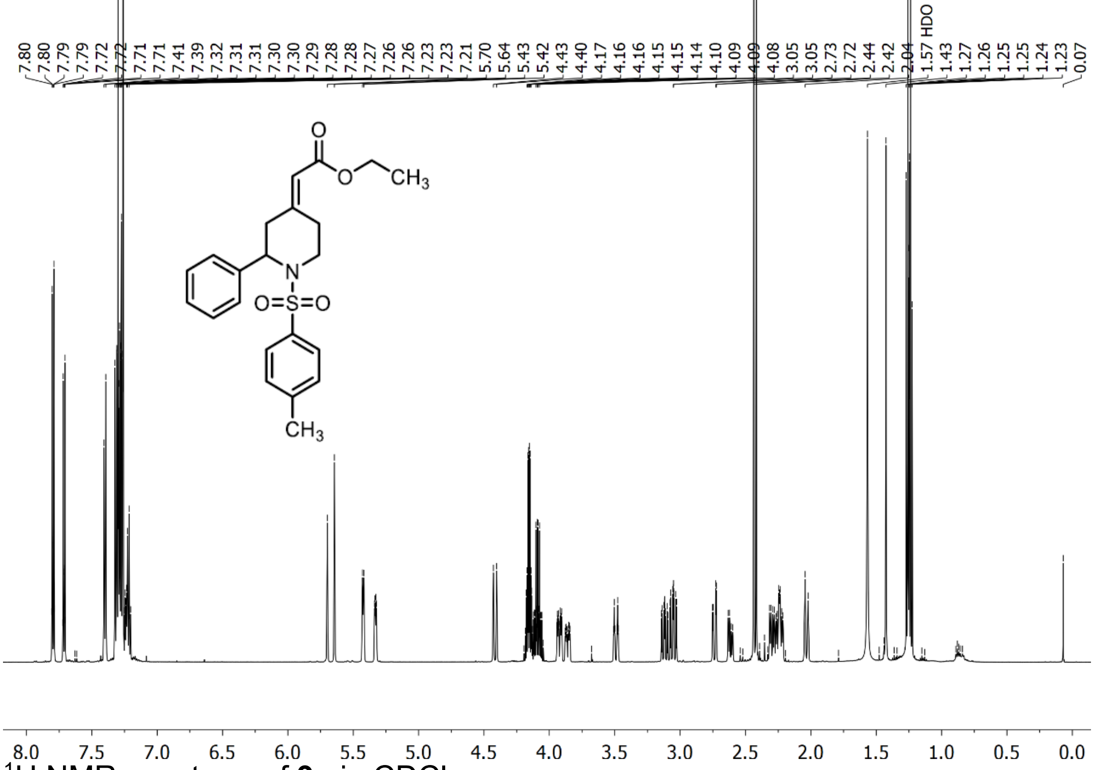

^{1}H NMR spectrum of **9a** in $CDCl_3$.

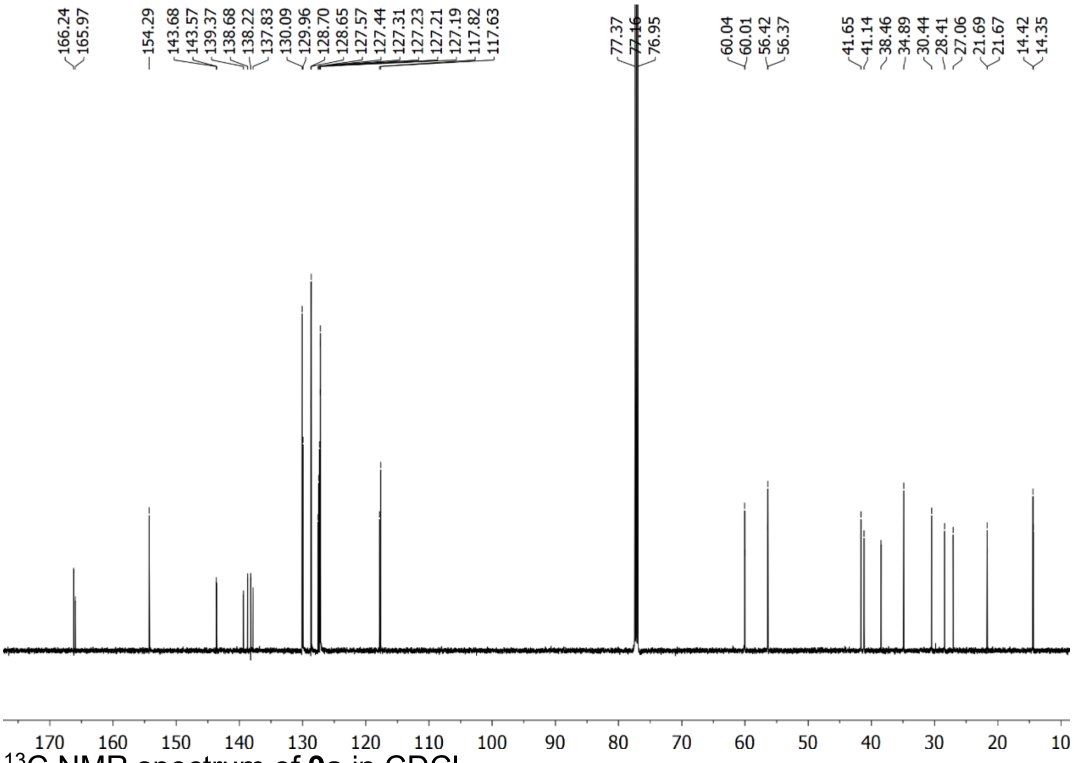

^{13}C NMR spectrum of **9a** in $CDCl_3$.

Benzyl (*E*)- and (*Z*)-4-(ethoxycarbonylmethylene)-2-phenylpiperidine-1-carboxylate (9b)

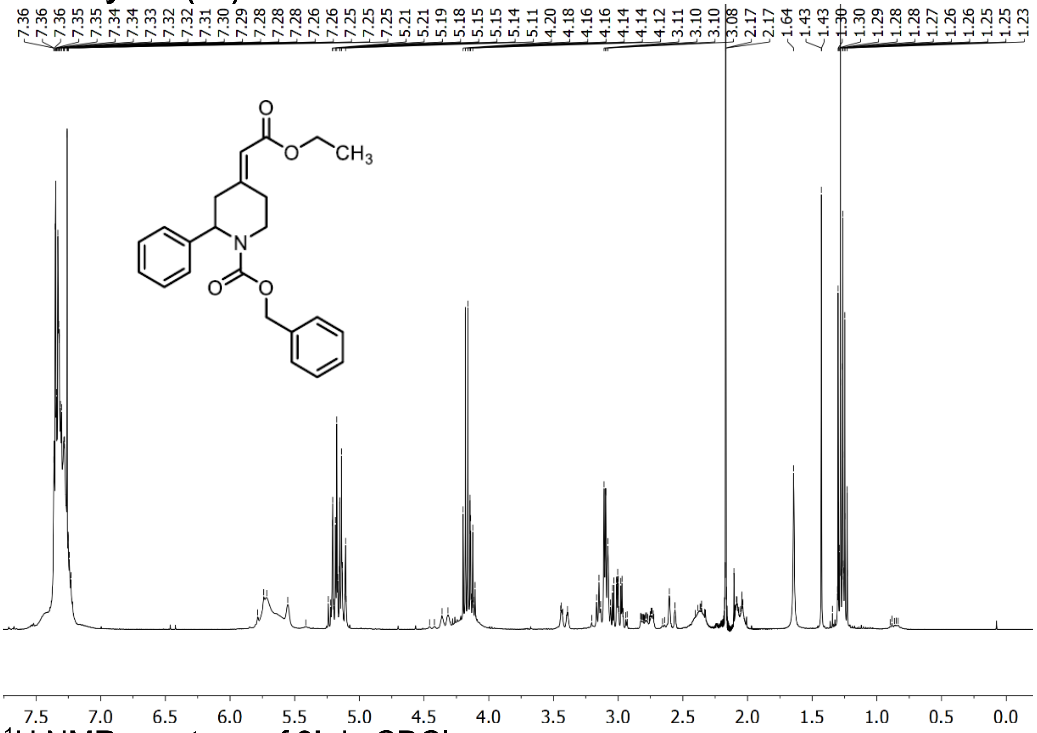

^{1}H NMR spectrum of **9b** in $CDCl_3$.

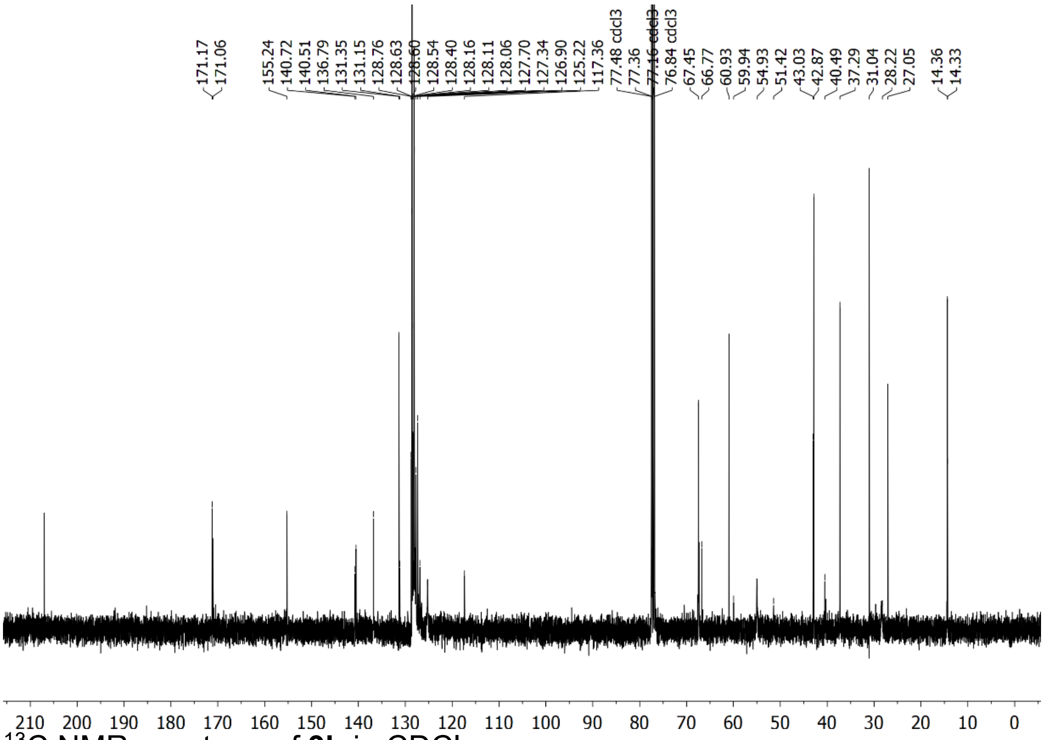

^{13}C NMR spectrum of **9b** in $CDCl_3$.

Ethyl *cis*- and *trans*-2-(2-phenyl-1-tosylpiperidin-4-yl)acetate (*cis*-10 and *trans*- 10)

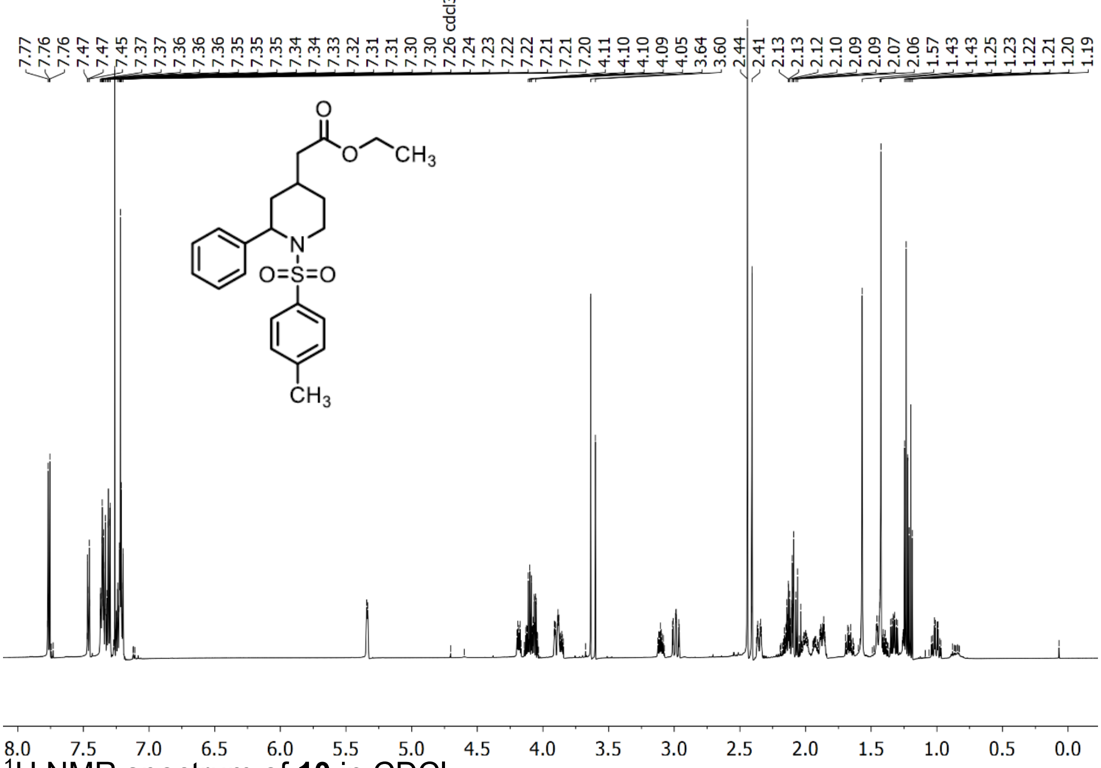

^{1}H NMR spectrum of **10** in $CDCl_3$.

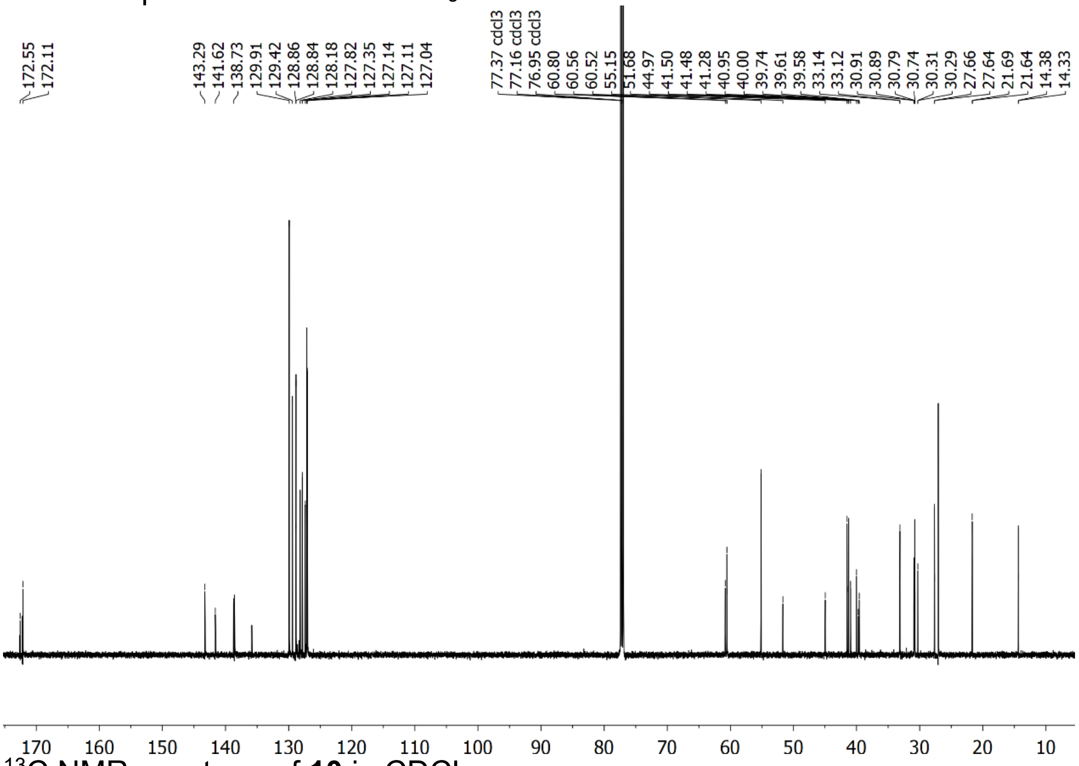

^{13}C NMR spectrum of **10** in $CDCl_3$.

cis- and _trans_-2-(2-Phenyl-1-tosylpiperidin-4-yl)ethan-1-ol (_cis_-11 and _trans_-11)

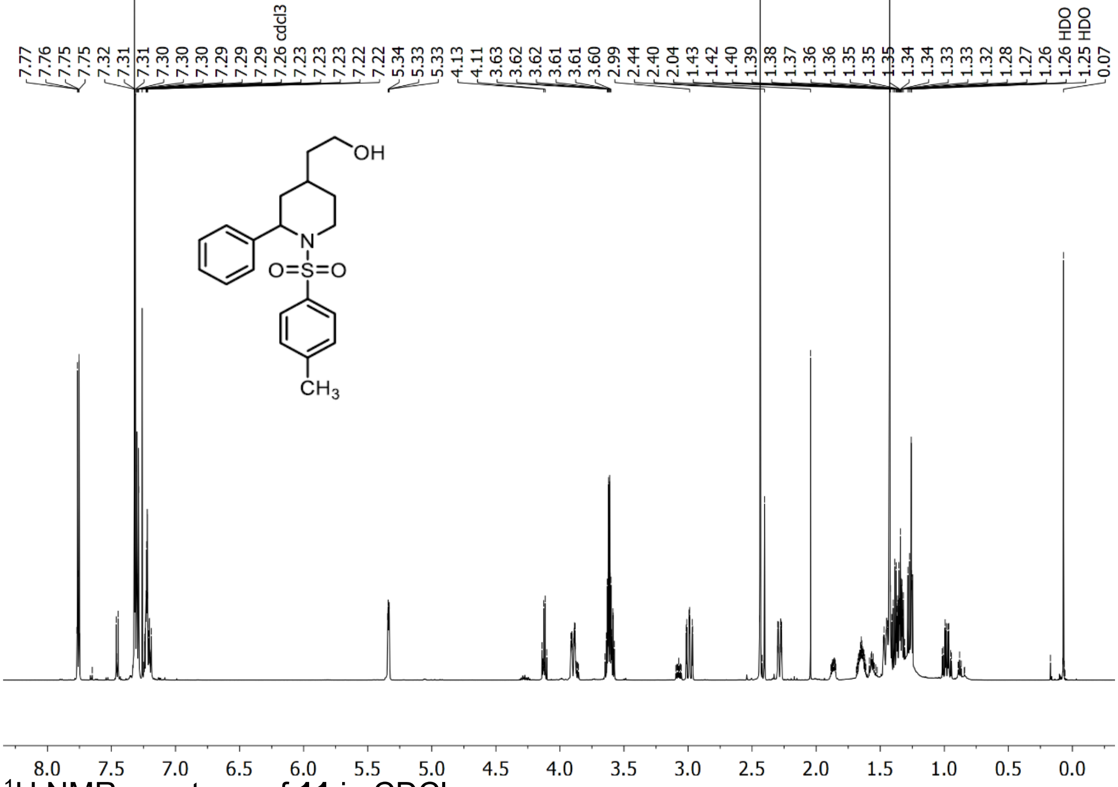

^{1}H NMR spectrum of **11** in $CDCl_3$.

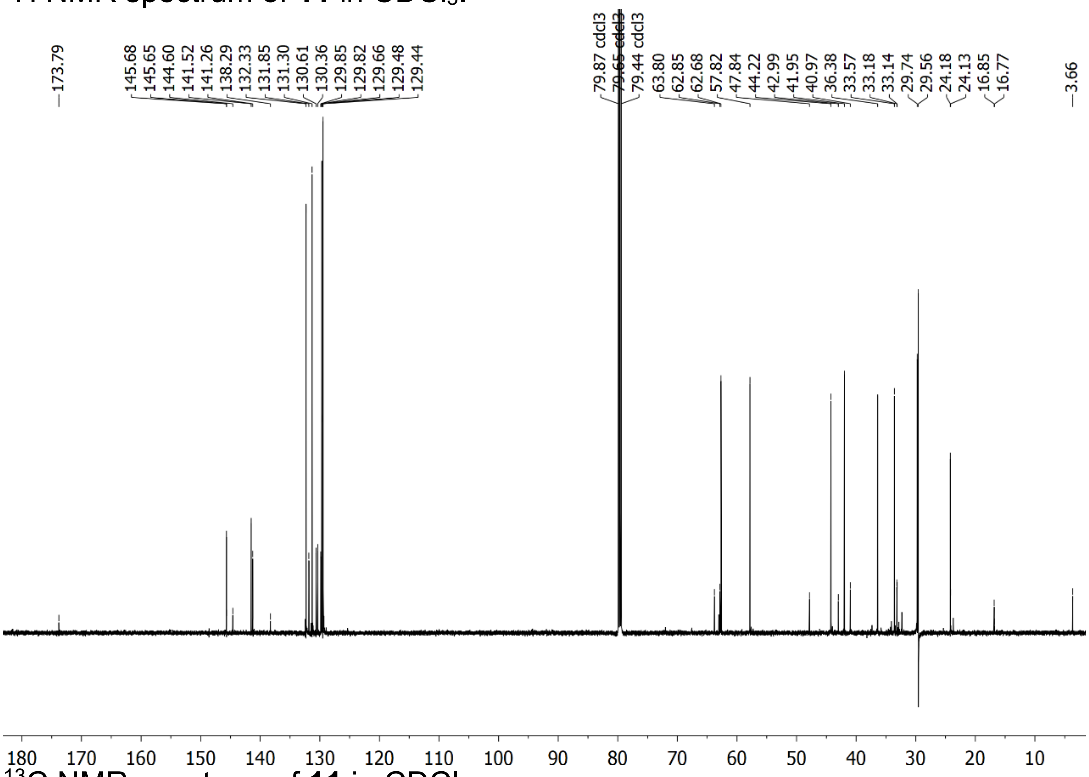

^{13}C NMR spectrum of **11** in $CDCl_3$.

cis- and *trans*-2-(2-Phenyl-1-tosylpiperidin-4-yl)ethyl methanesulfonate (*cis*-12 and *trans*-12)

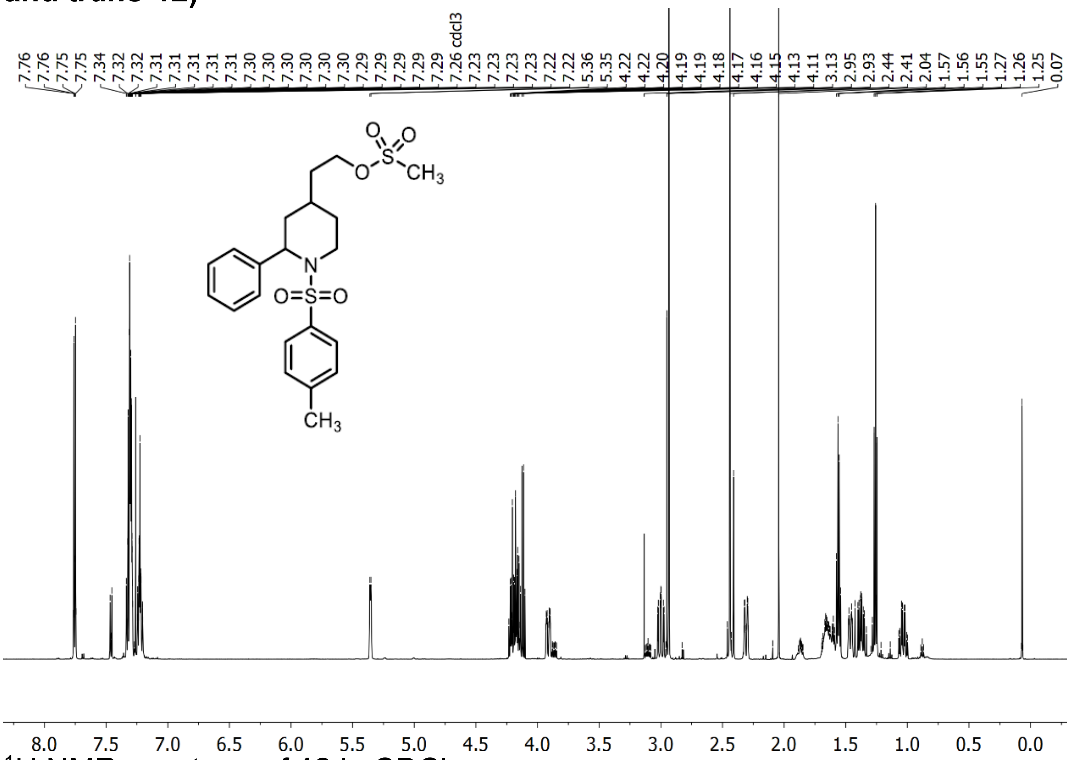

^1H NMR spectrum of **12** in $CDCl_3$.

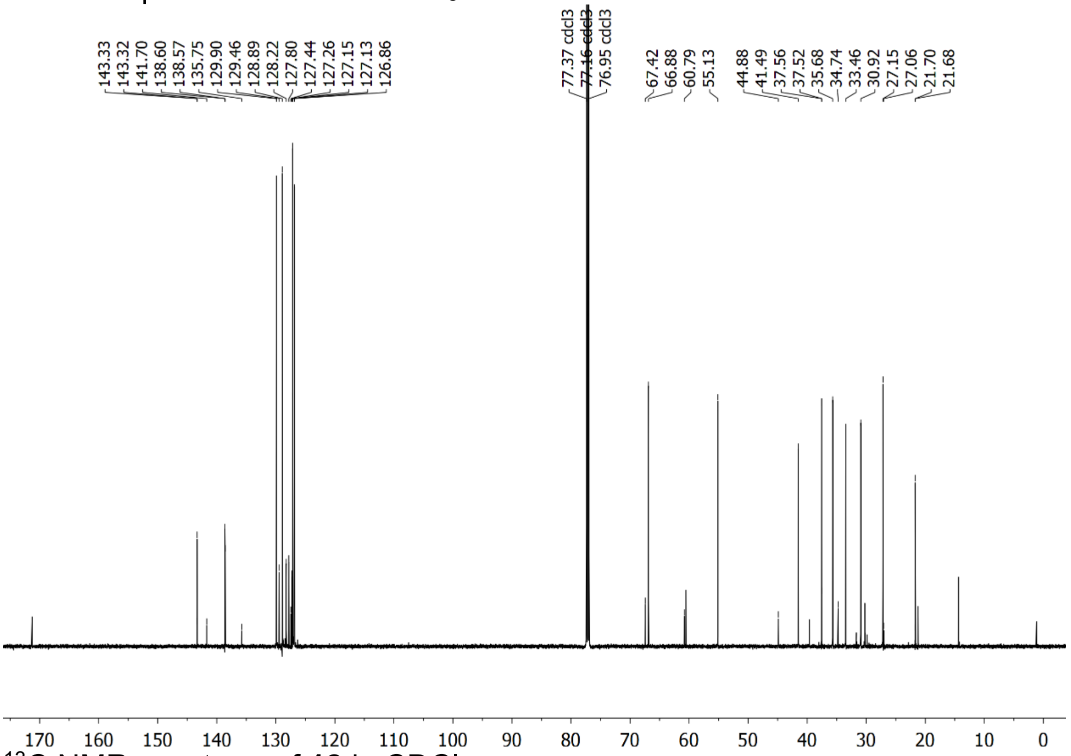

^{13}C NMR spectrum of **12** in $CDCl_3$.

cis- and *trans*-N-Benzyl-2-(2-phenyl-1-tosylpiperidin-4-yl)ethan-1-amine (*cis*-13a and *trans*-13a)

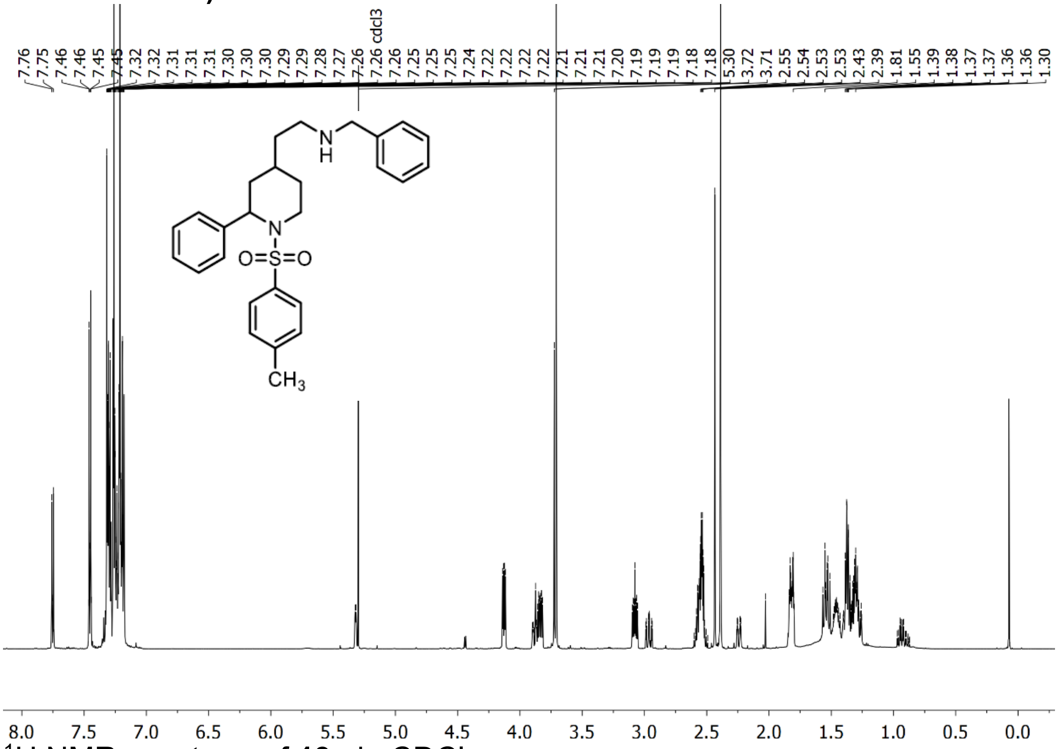

^{1}H NMR spectrum of **13a** in $CDCl_3$.

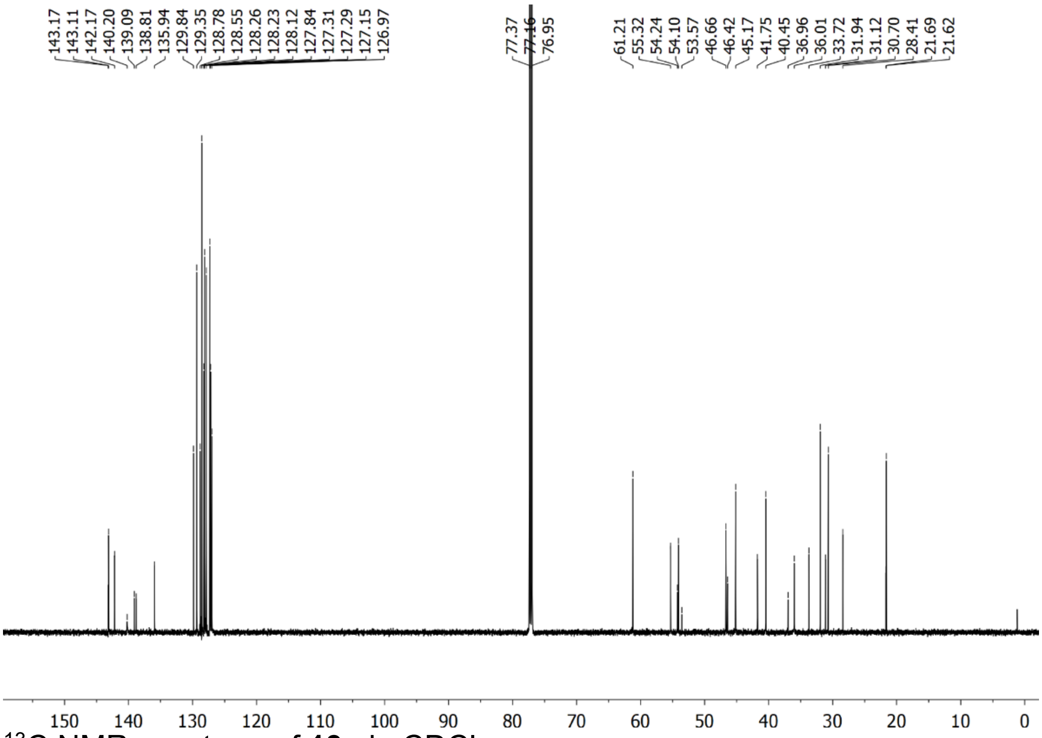

^{13}C NMR spectrum of **13a** in $CDCl_3$.

cis- and _trans_-3-Phenyl-N-[2-(2-phenyl-1-tosylpiperidin-4-yl)ethyl]propan-1- amine (_cis_-13b and _trans_-13b)

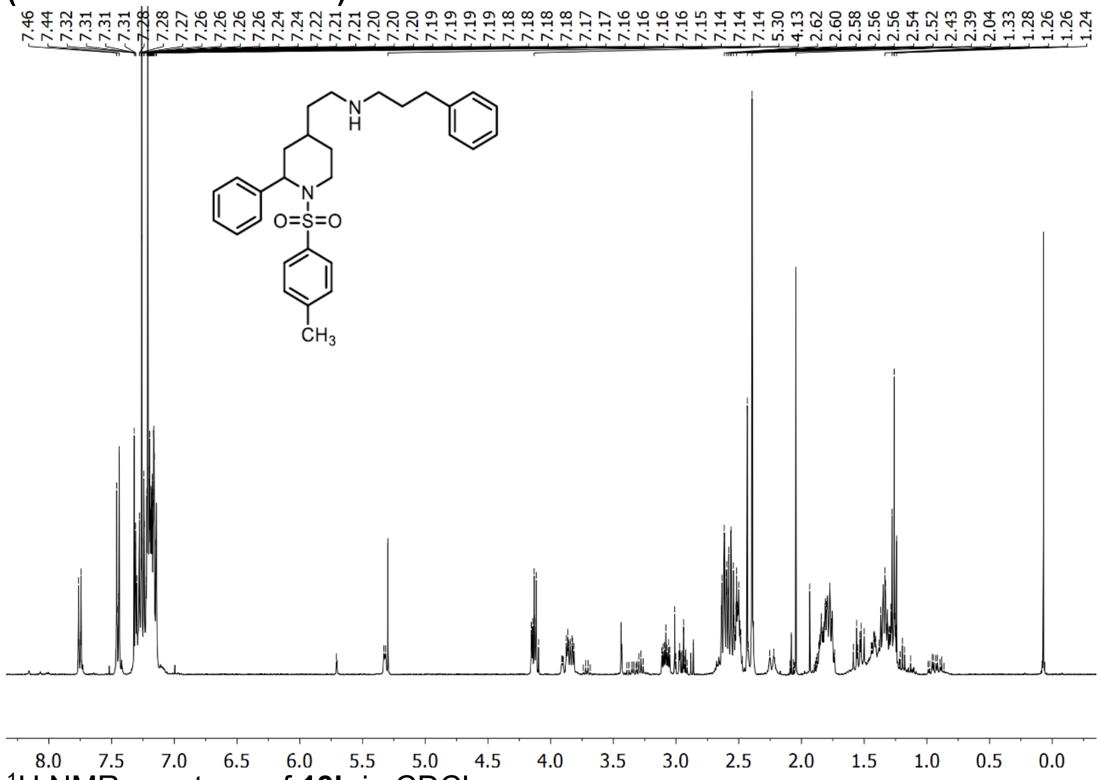

^{1}H NMR spectrum of **13b** in $CDCl_3$.

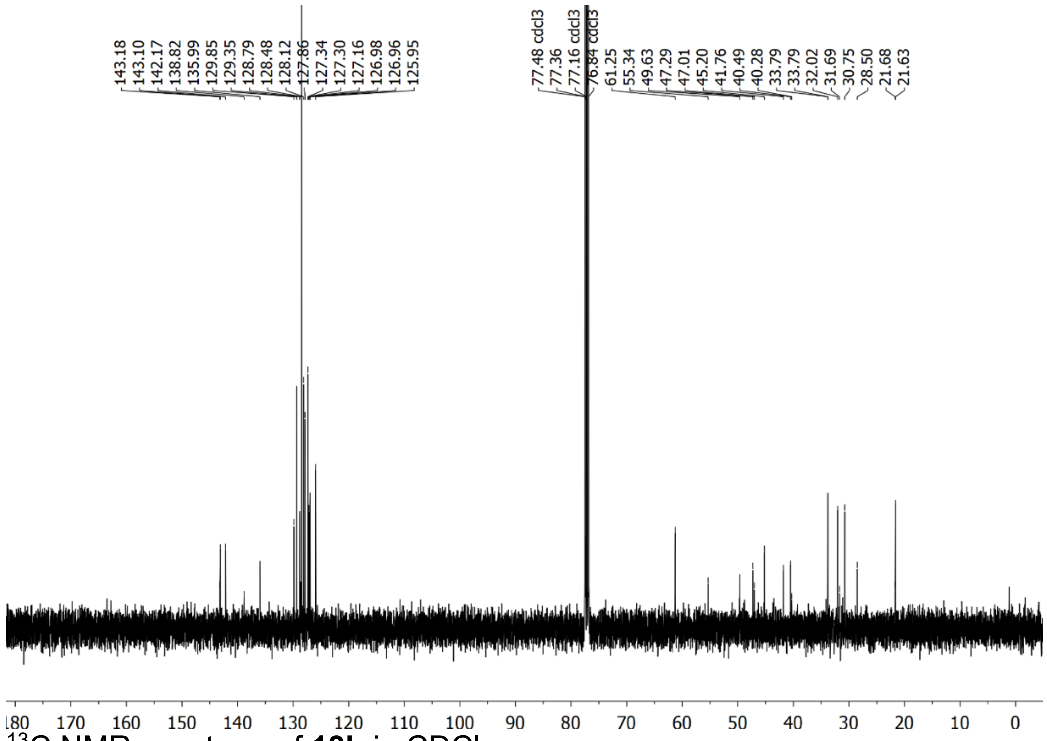

^{13}C NMR spectrum of **13b** in $CDCl_3$.

***cis*- and *trans*-N-Benzyl-2-(2-phenylpiperidin-4-yl)ethan-1-amine (*cis*-4a and *trans*-4a)**

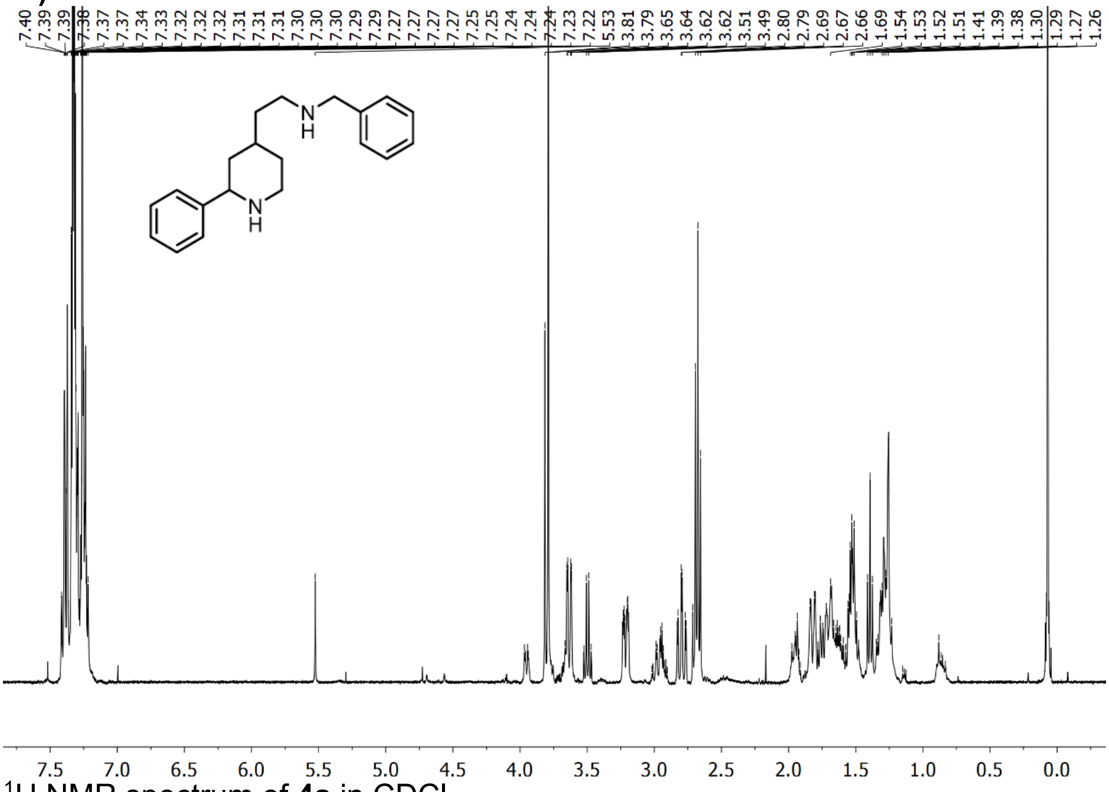

^{1}H NMR spectrum of **4a** in $CDCl_3$.

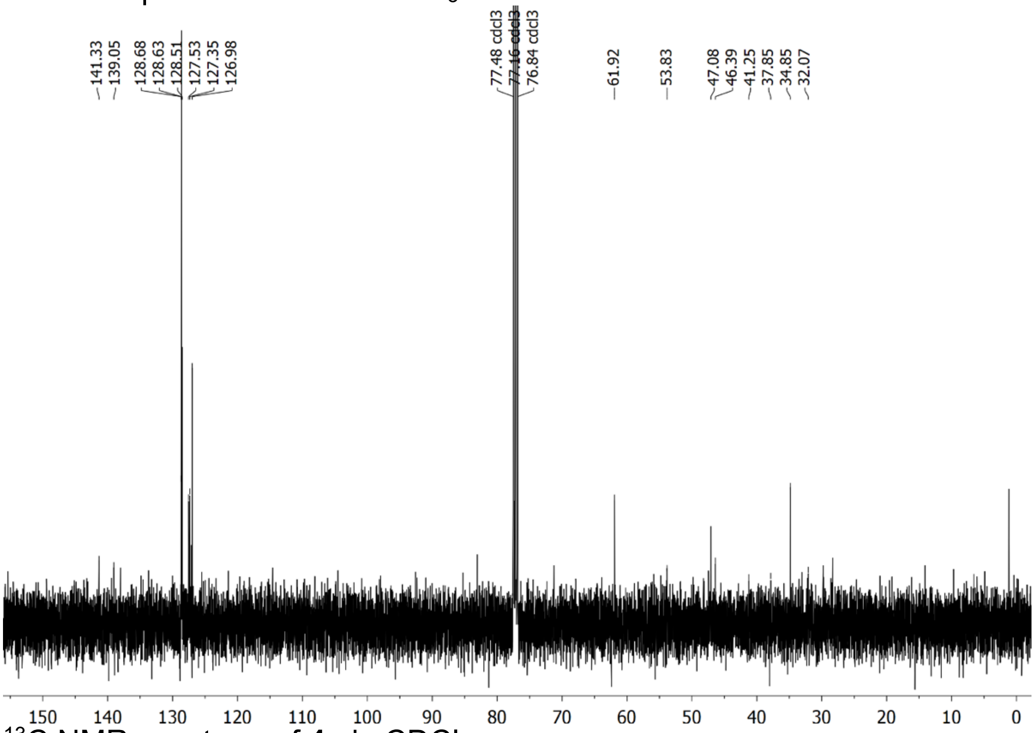

^{13}C NMR spectrum of **4a** in $CDCl_3$.

***cis*- and *trans*-3-Phenyl-N-[2-(2-phenylpiperidin-4-yl)ethyl]propan-1-amine (*cis*-4b /*trans*-4b)**

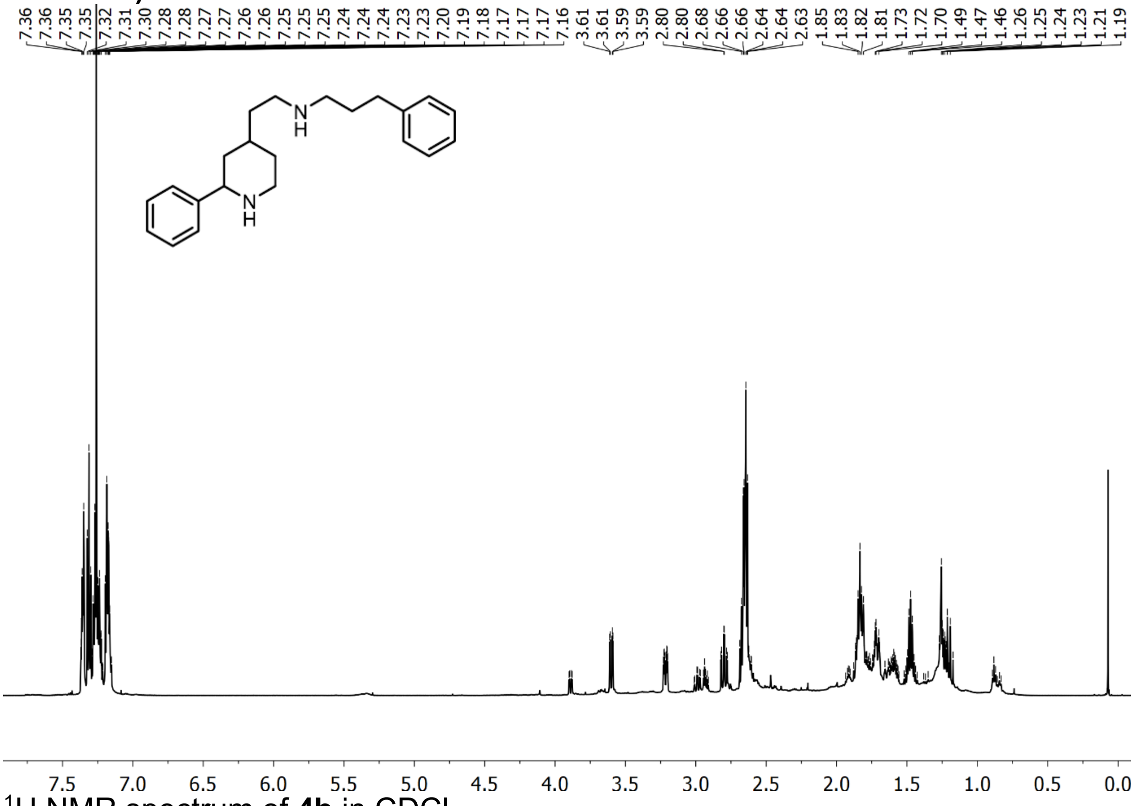

^{1}H NMR spectrum of **4b** in $CDCl_3$.

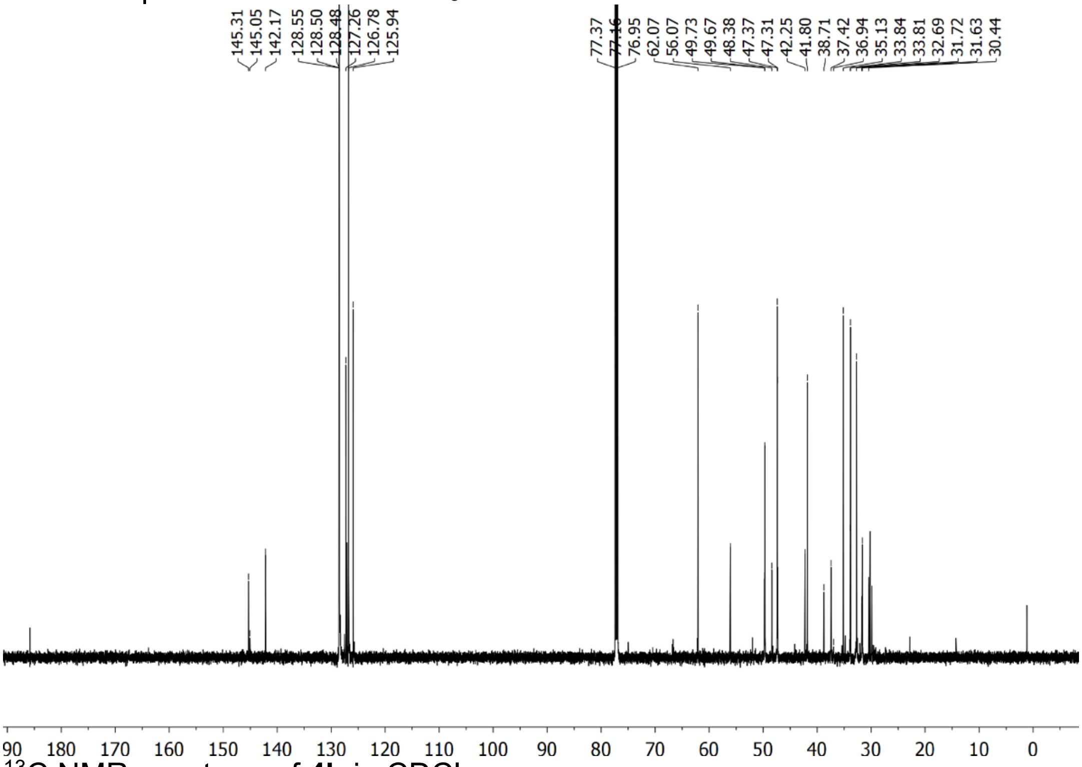

^{13}C NMR spectrum of **4b** in $CDCl_3$.

Ethyl 2-(2-phenylpiperidin-4-yl)acetate (*cis*-14 and *trans*-14)

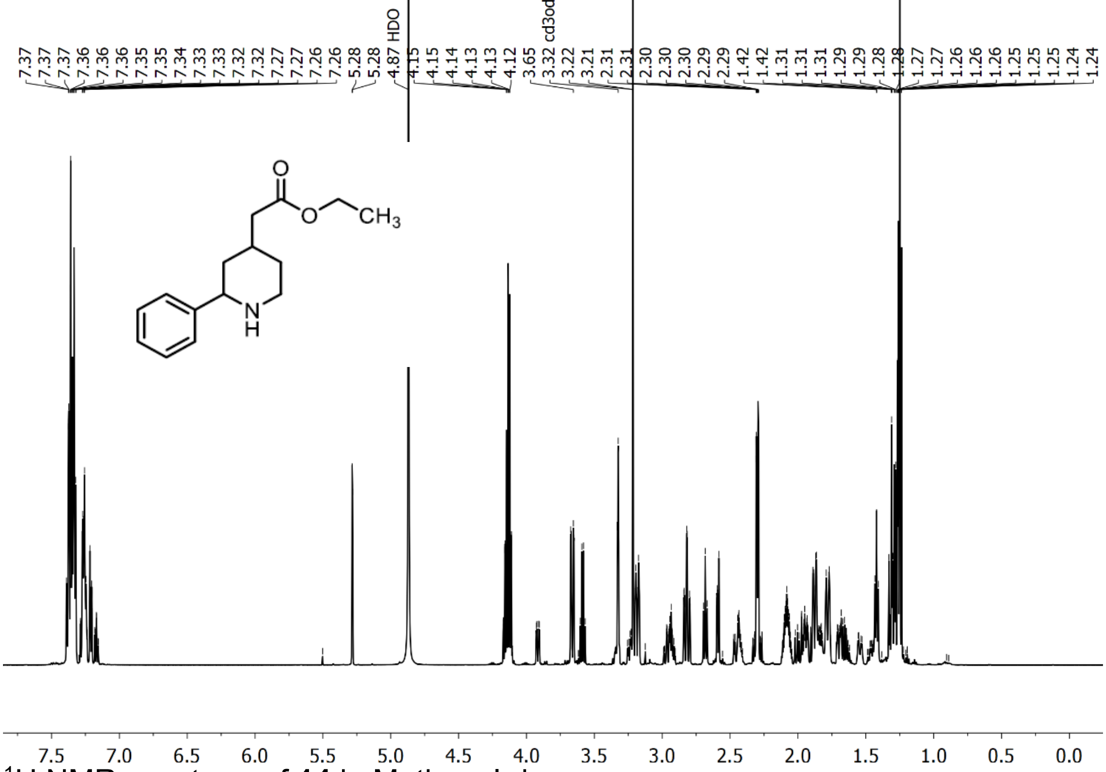

^{1}H NMR spectrum of **14** in Methanol-d$_4$.

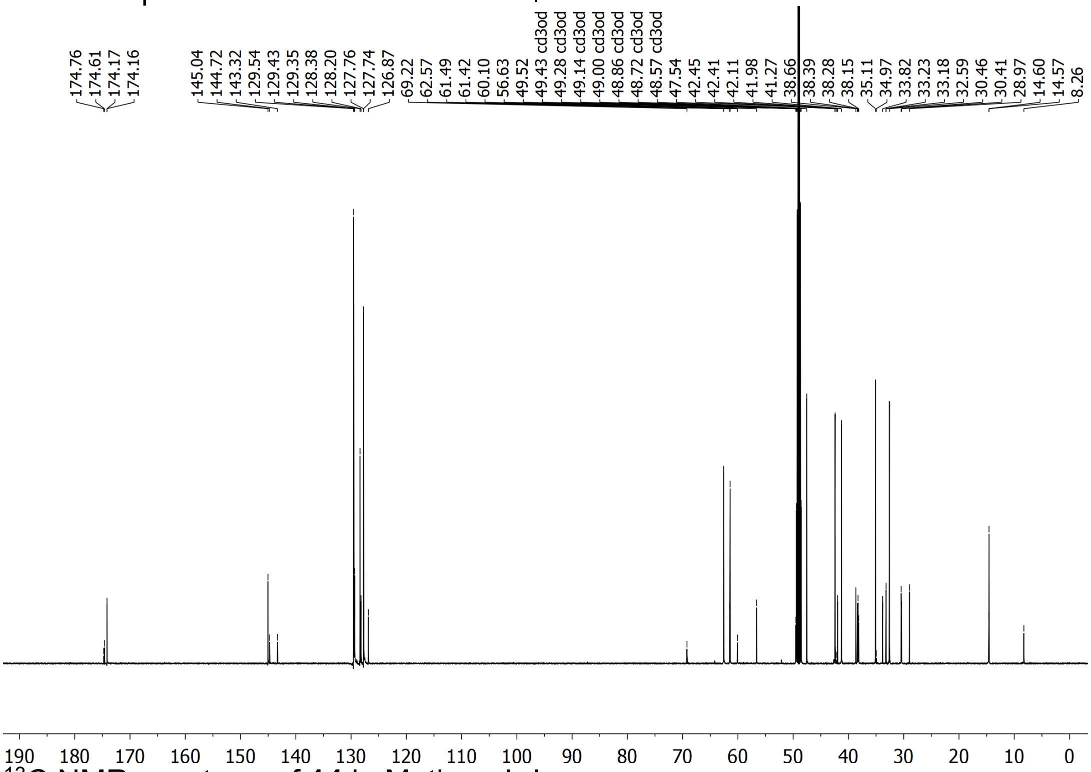

^{13}C NMR spectrum of **14** in Methanol-d$_4$.

Ethyl *cis*- and *trans*-2-(1-methyl-2-phenylpiperidin-4-yl)acetate (*cis*-15aa and *trans*-15a)

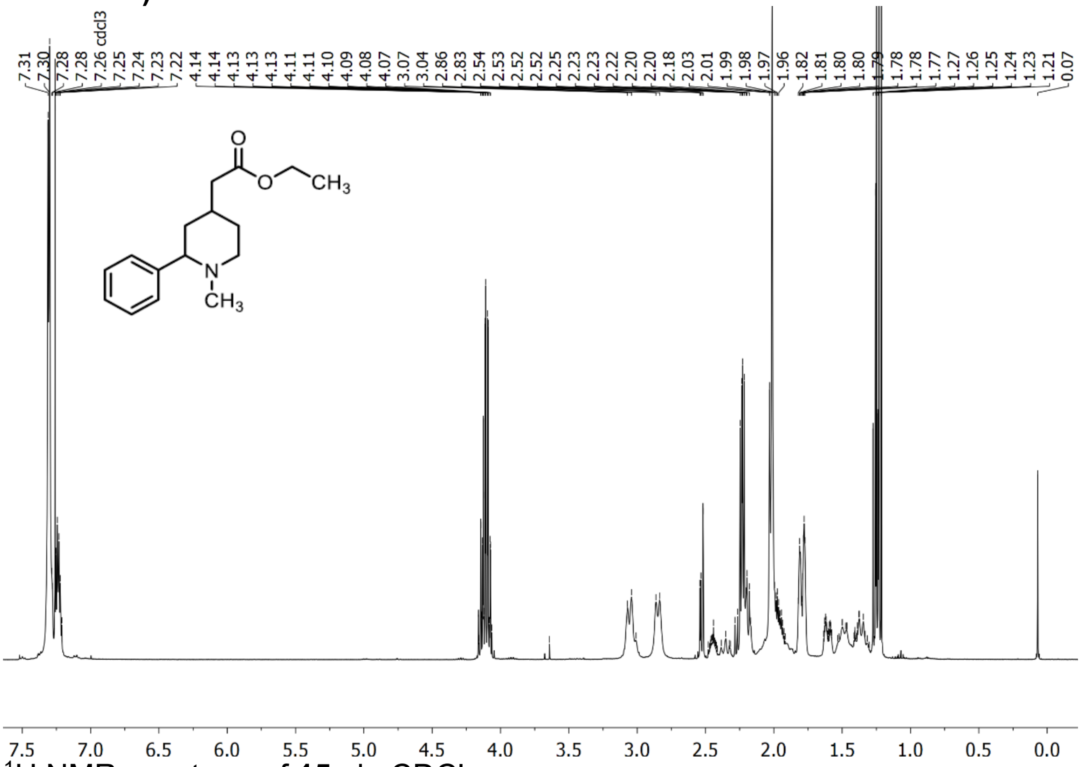

^{1}H NMR spectrum of **15a** in $CDCl_3$.

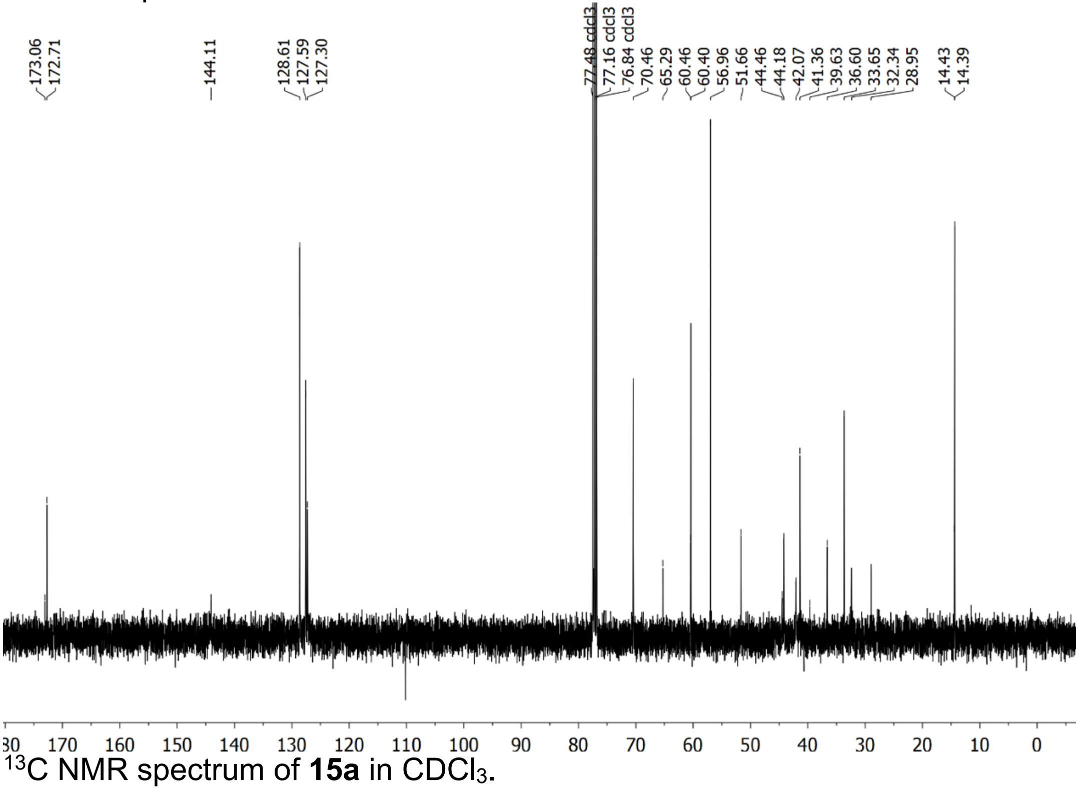

^{13}C NMR spectrum of **15a** in $CDCl_3$.

Ethyl *cis*- and *trans*-2-(1-ethyl-2-phenylpiperidin-4-yl)acetate (*cis*-15b and *trans*-15b)

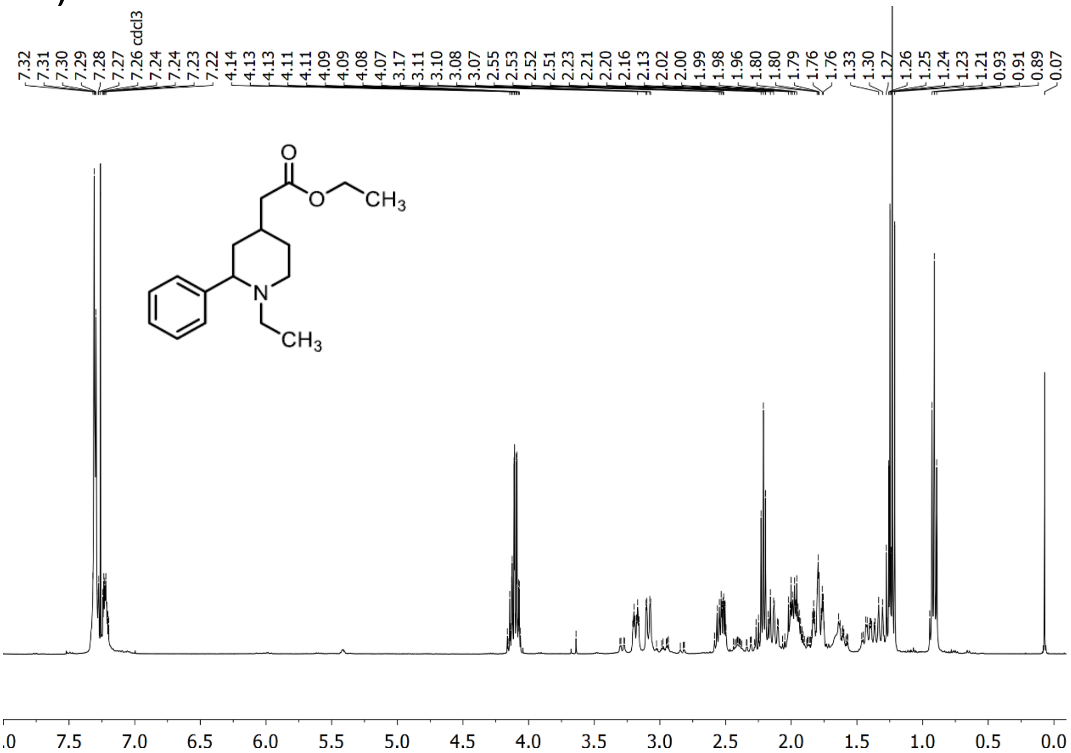

^{1}H NMR spectrum of **15b** in $CDCl_3$.

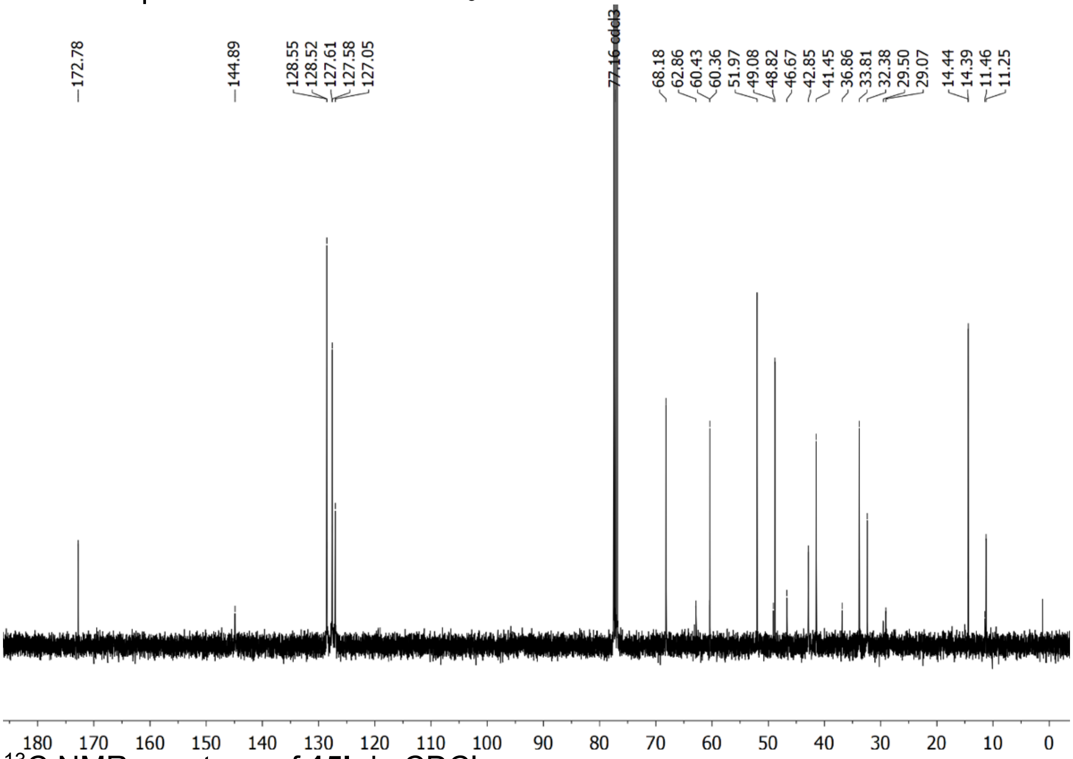

^{13}C NMR spectrum of **15b** in $CDCl_3$.

cis- and _trans_-2-(1-Methyl-2-phenylpiperidin-4-yl)ethan-1-ol (_cis_-16a and _trans_-16a)

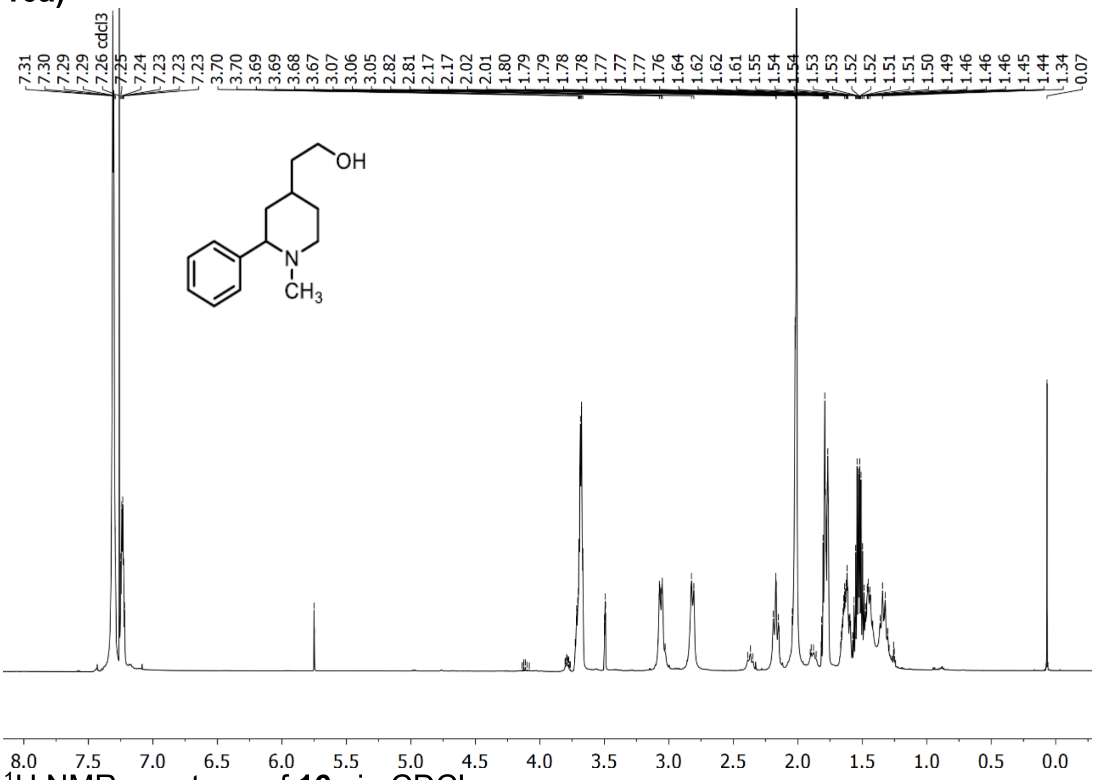

^{1}H NMR spectrum of **16a** in $CDCl_3$.

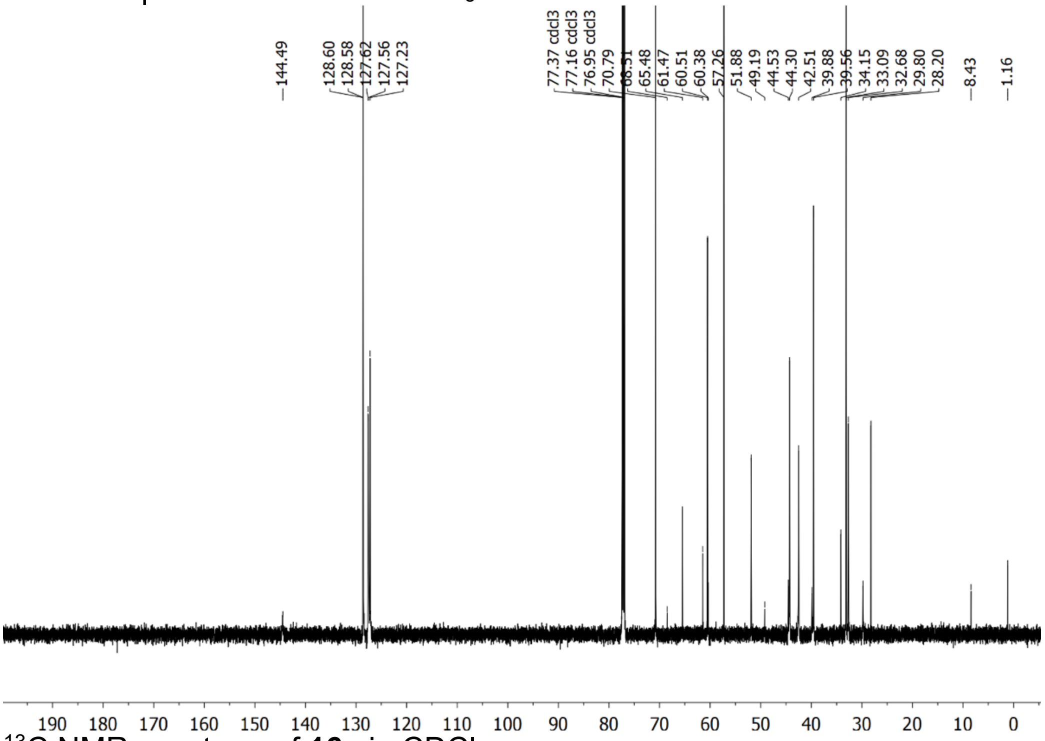

^{13}C NMR spectrum of **16a** in $CDCl_3$.

cis- and _trans_-2-(1-Ethyl-2-phenylpiperidin-4-yl)ethan-1-ol (_cis_-16b and _trans_-16b)

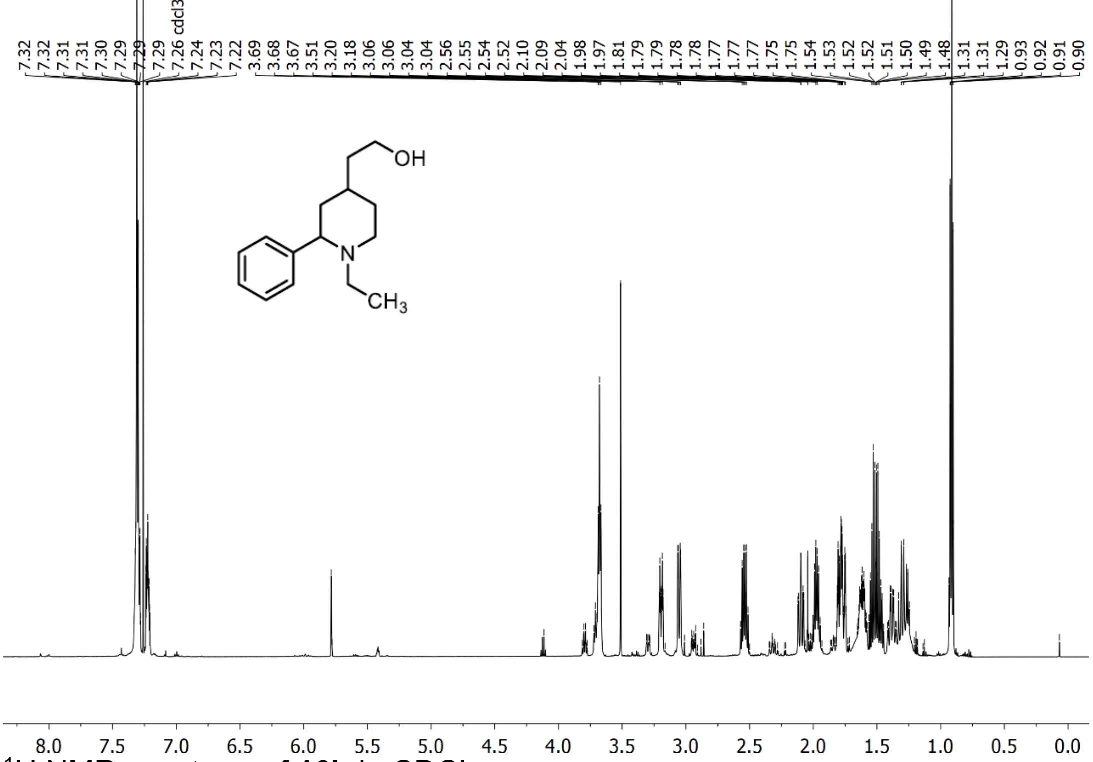

^{1}H NMR spectrum of **16b** in $CDCl_3$.

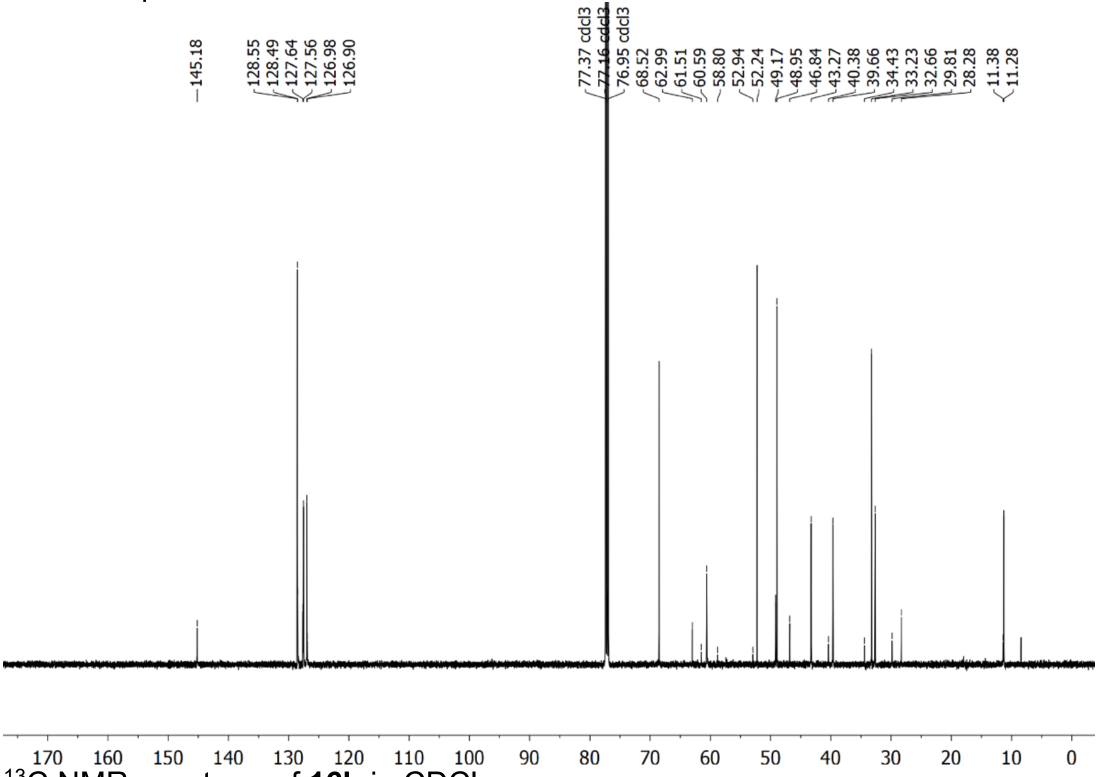

^{13}C NMR spectrum of **16b** in $CDCl_3$.

***cis*- and *trans*-2-(1-Methyl-2-phenylpiperidin-4-yl)acetaldehyde (*cis*-17a and *trans*-17a)**

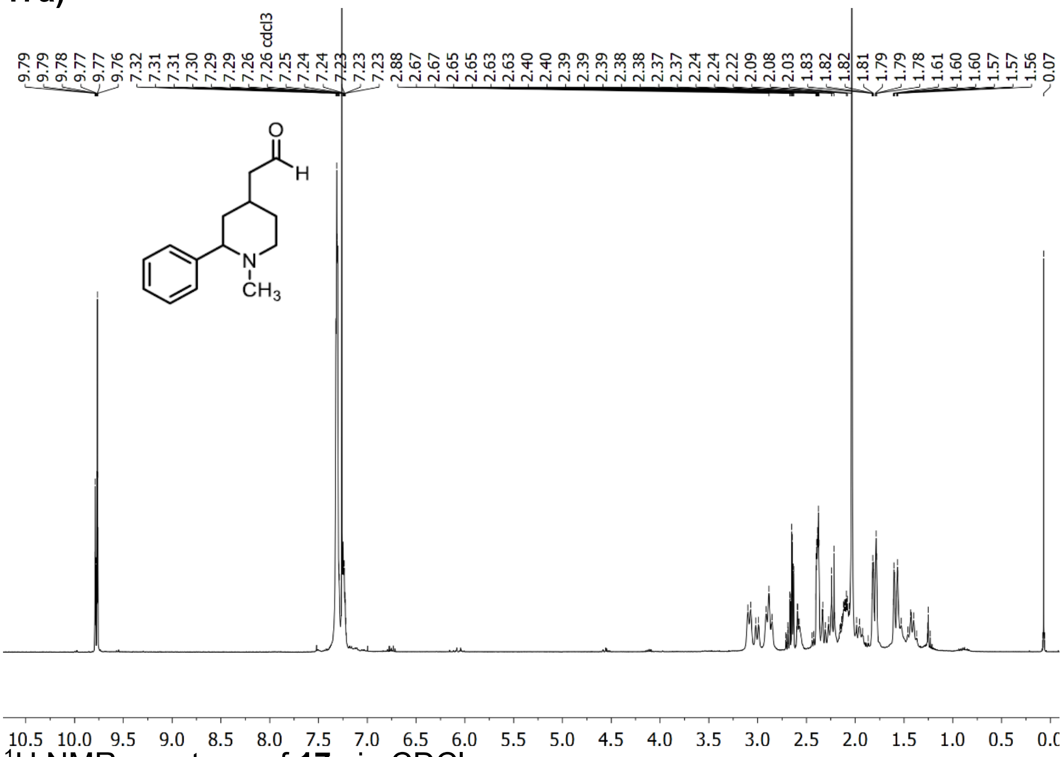

^{1}H NMR spectrum of **17a** in $CDCl_3$.

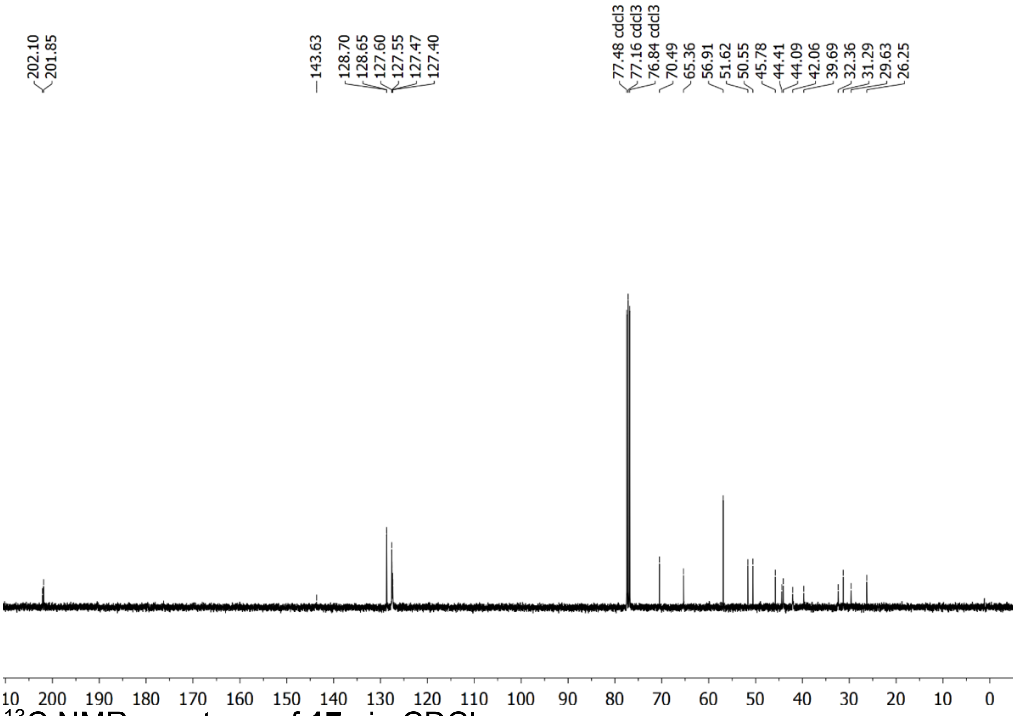

^{13}C NMR spectrum of **17a** in $CDCl_3$.

cis- and *trans*-2-(1-Ethyl-2-phenylpiperidin-4-yl)acetaldehyde (*cis*-17b and *trans*-17b)

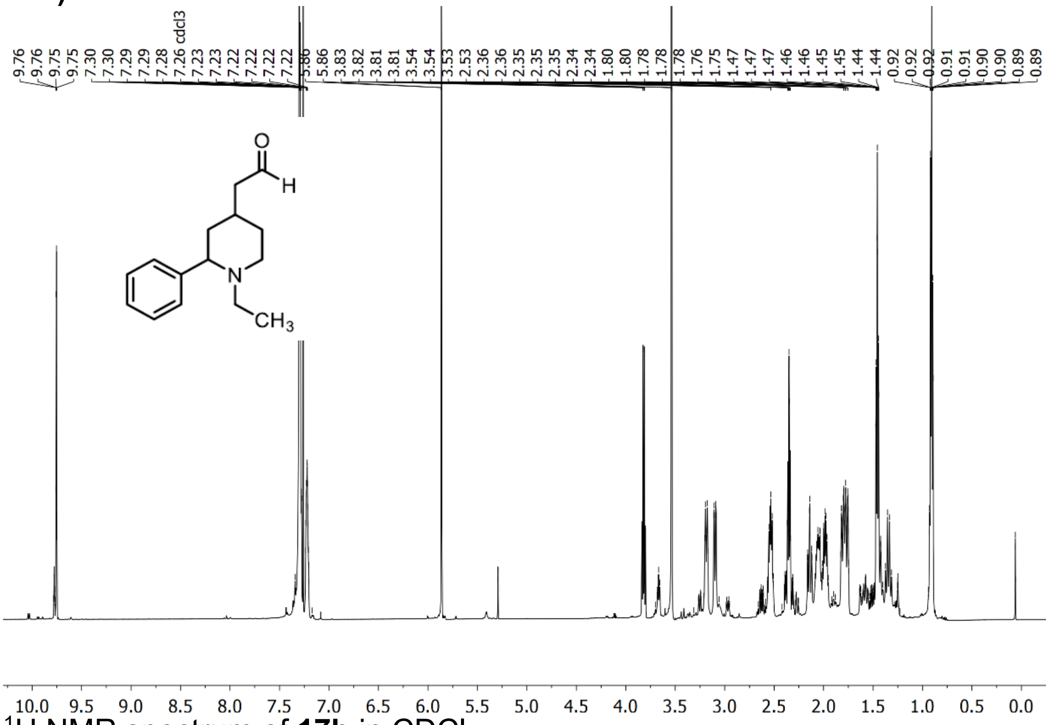

^{1}H NMR spectrum of **17b** in $CDCl_3$.

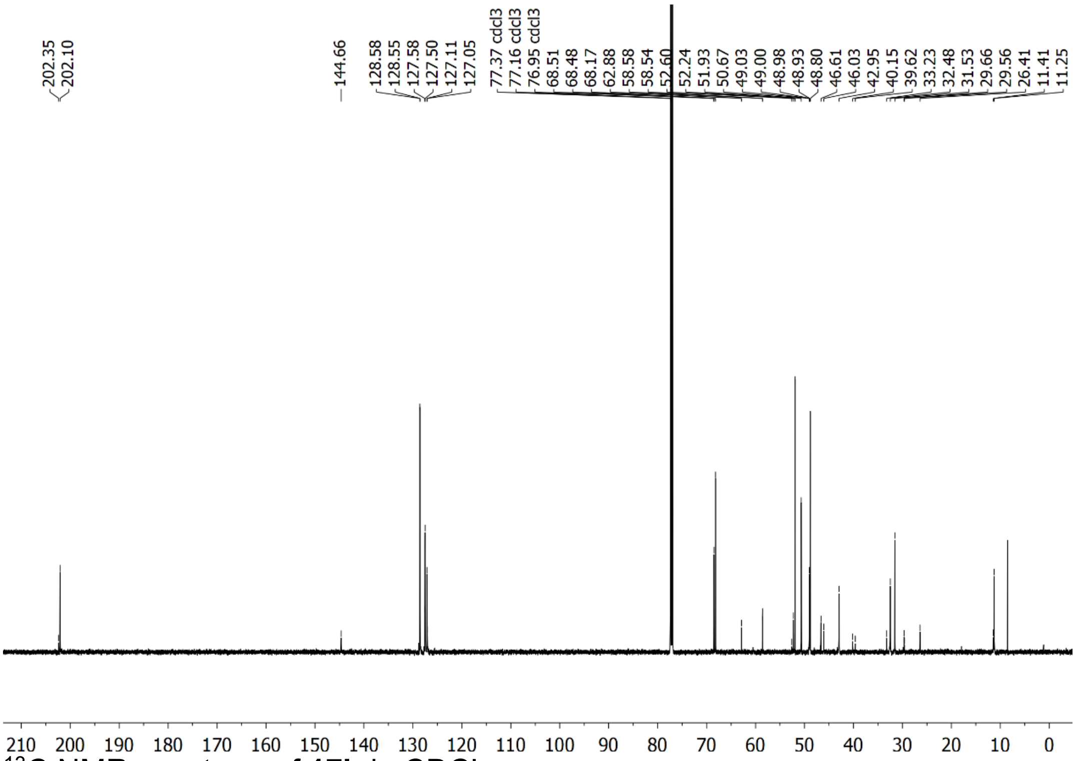

^{13}C NMR spectrum of **17b** in $CDCl_3$.

cis- and *trans*-N-Benzyl-2-(1-methyl-2-phenylpiperidin-4-yl)ethan-1-amine (*cis*-18a and *trans*-18a)

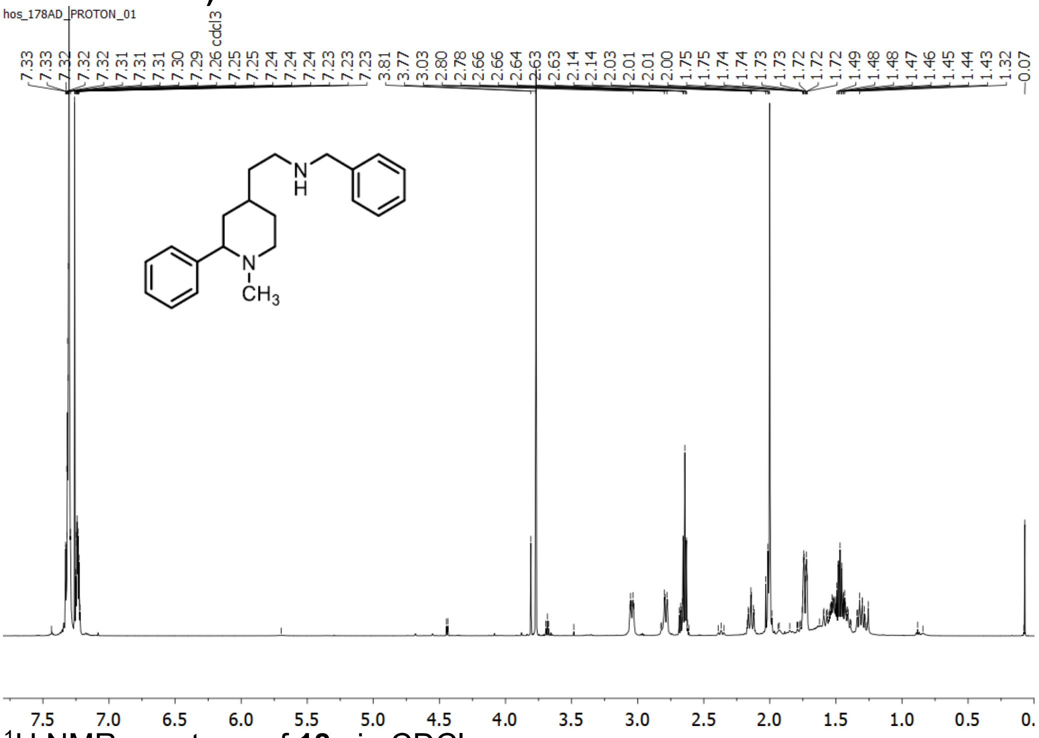

^{1}H NMR spectrum of **18a** in $CDCl_3$.

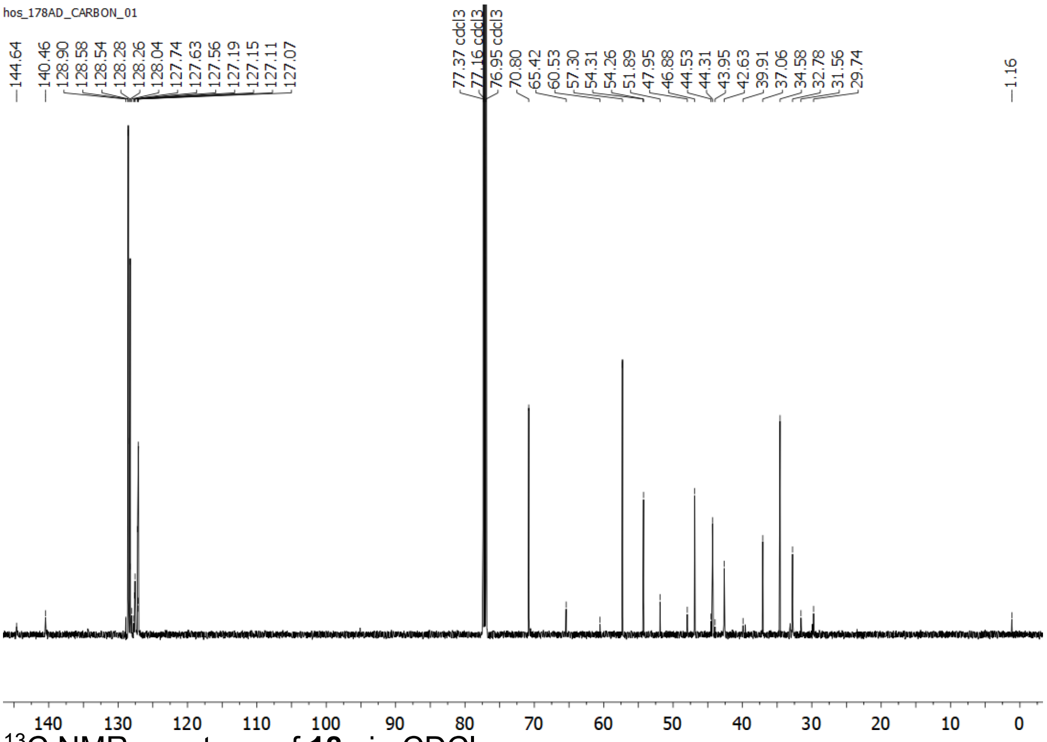

^{13}C NMR spectrum of **18a** in $CDCl_3$.

***cis*- and *trans*-N-(2-(1-Methyl-2-phenylpiperidin-4-yl)ethyl)-3-phenylpropan-1-amine (*cis*-19a and *trans*-19a)**

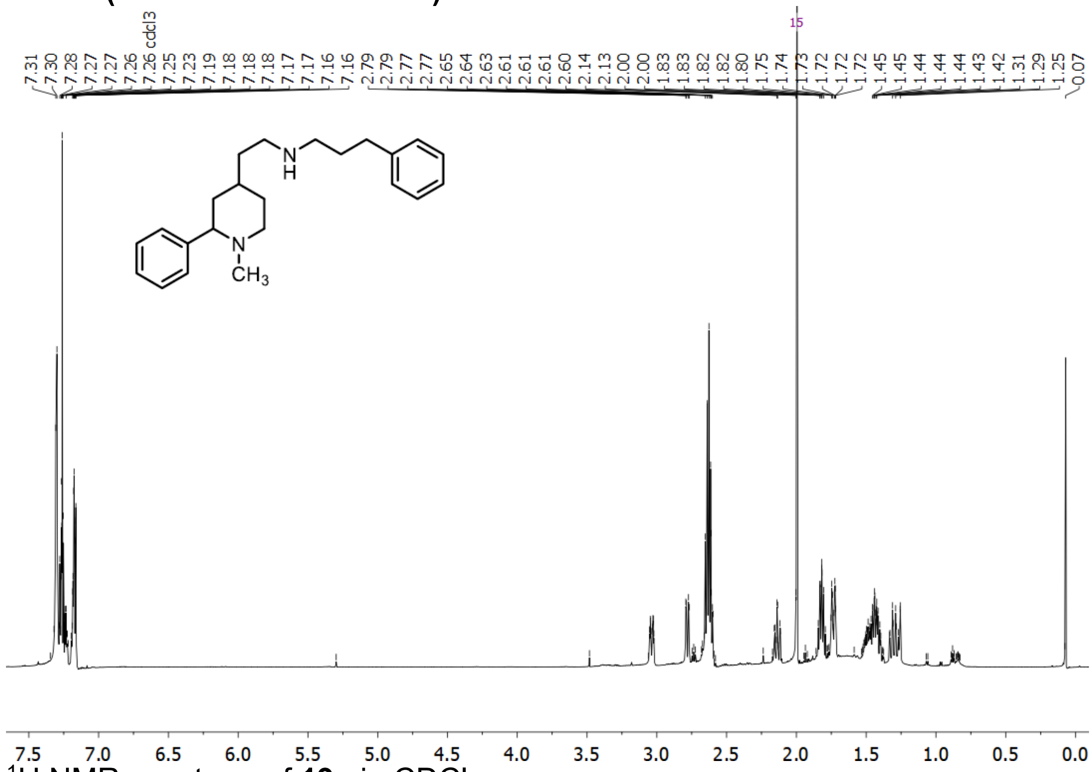

^{1}H NMR spectrum of **19a** in $CDCl_3$.

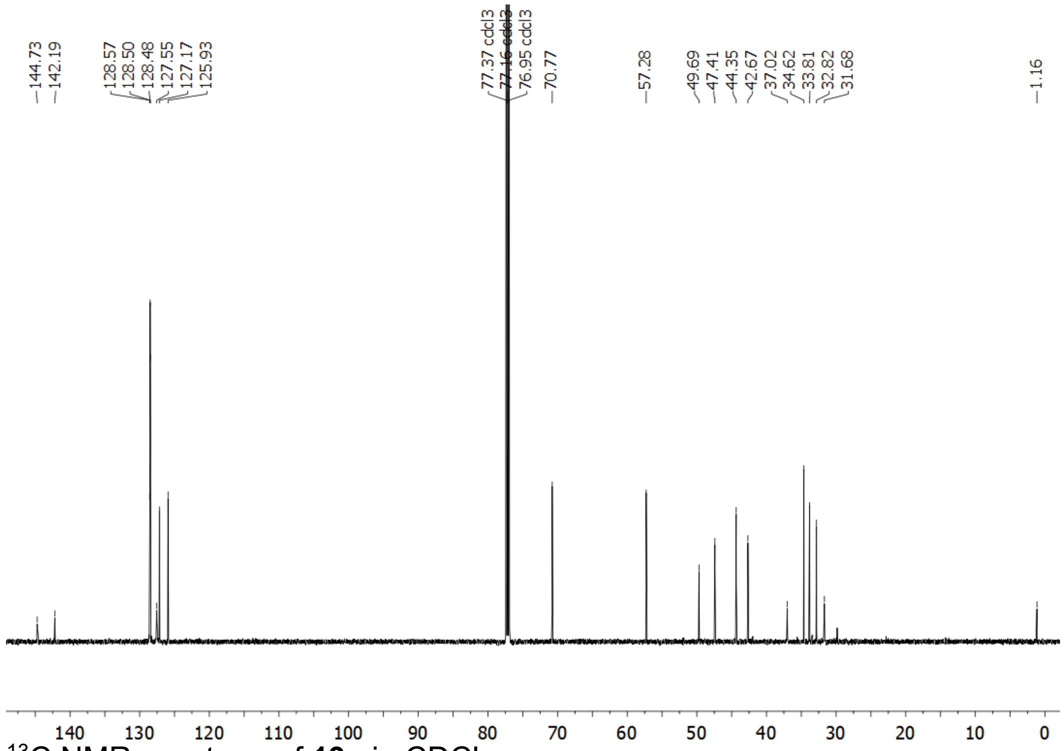

^{13}C NMR spectrum of **19a** in $CDCl_3$.

cis- and _trans_-N-Benzyl-2-(1-ethyl-2-phenylpiperidin-4-yl)ethan-1-amine (_cis_-18b and _trans_-18b)

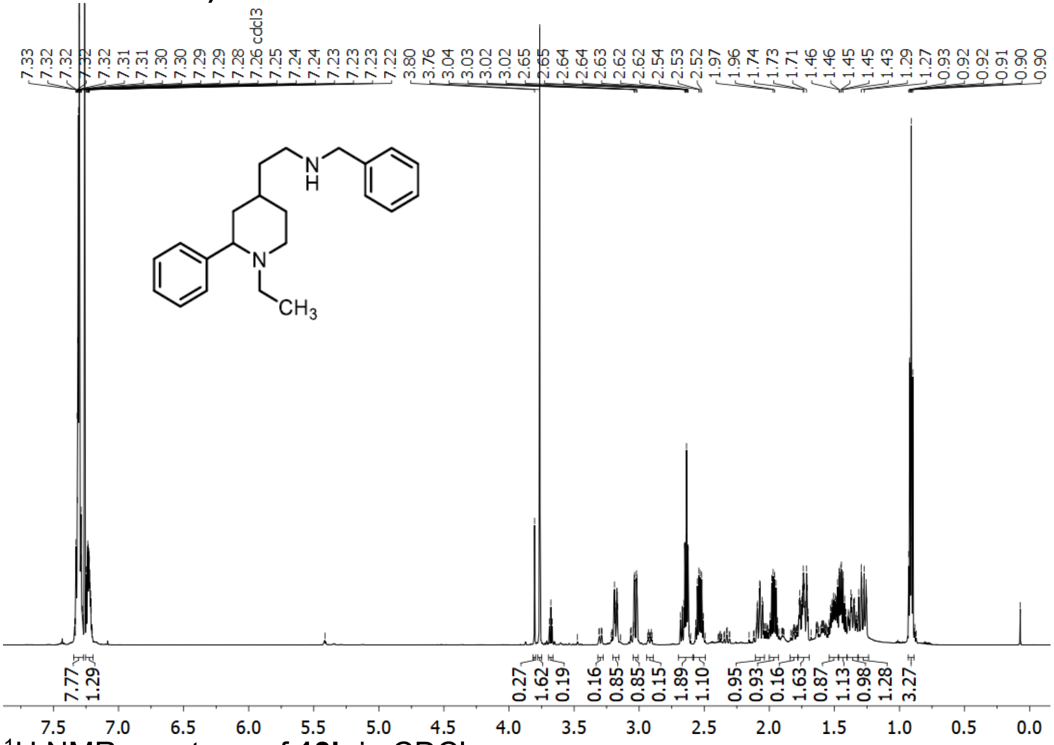

^{1}H NMR spectrum of **18b** in $CDCl_3$.

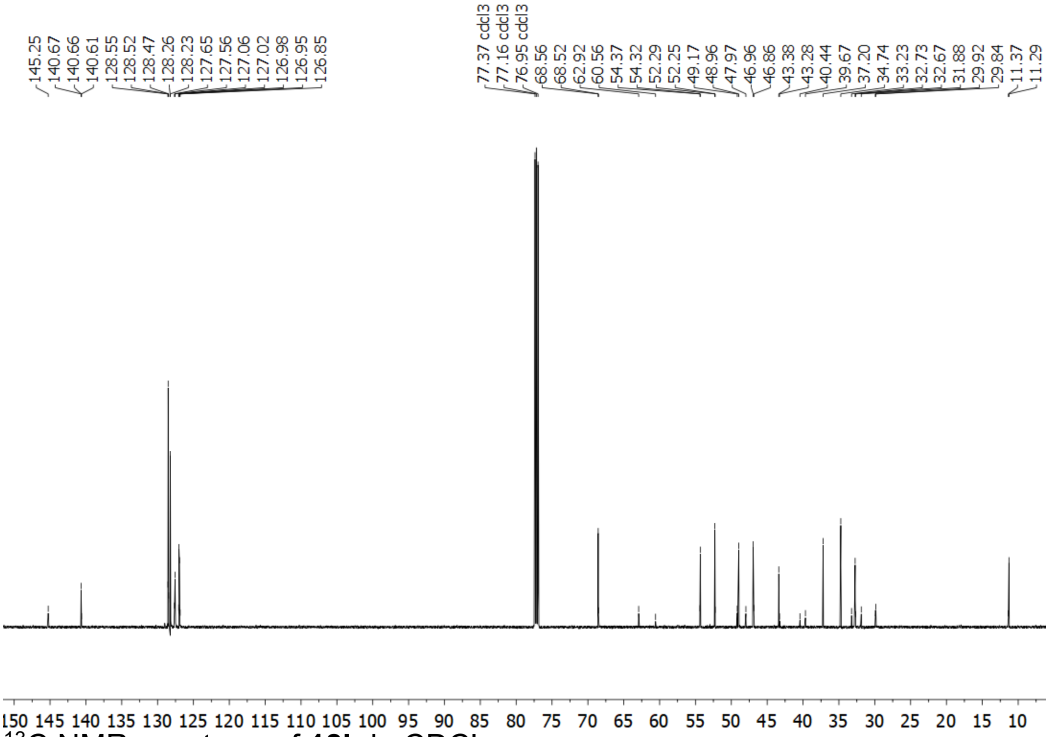

^{13}C NMR spectrum of **18b** in $CDCl_3$.

cis- and _trans_-N-(2-(1-Ethyl-2-phenylpiperidin-4-yl)ethyl)-3-phenylpropan-1-amine (_cis_-19b and _trans_-19b)

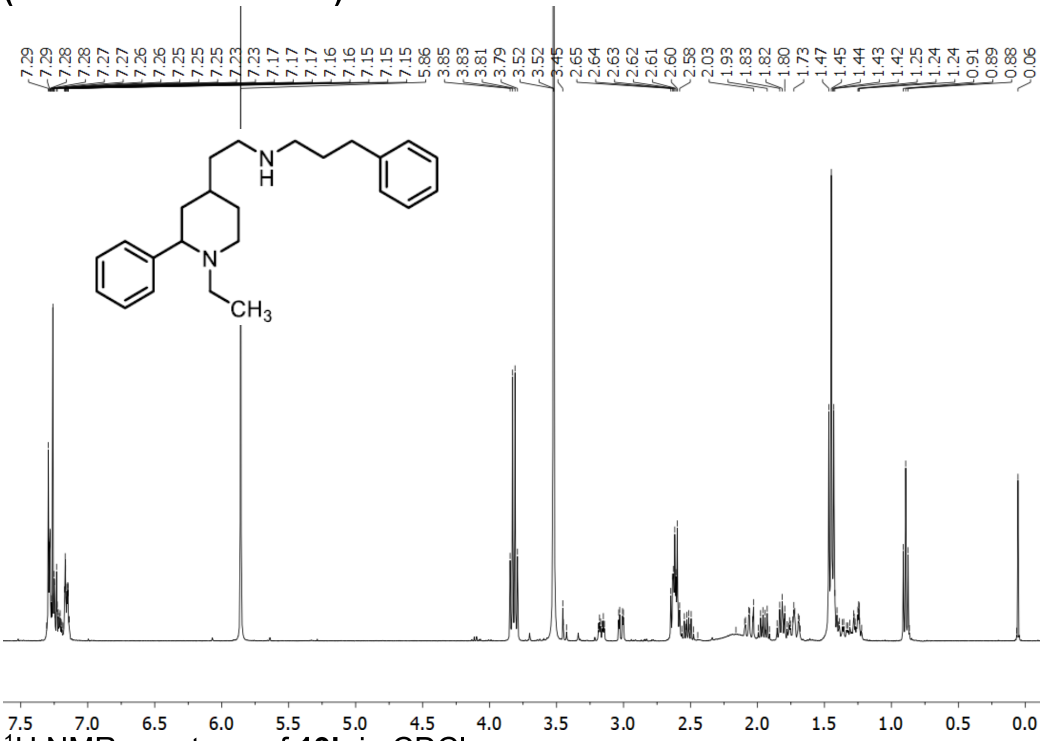

^{1}H NMR spectrum of **19b** in $CDCl_3$.

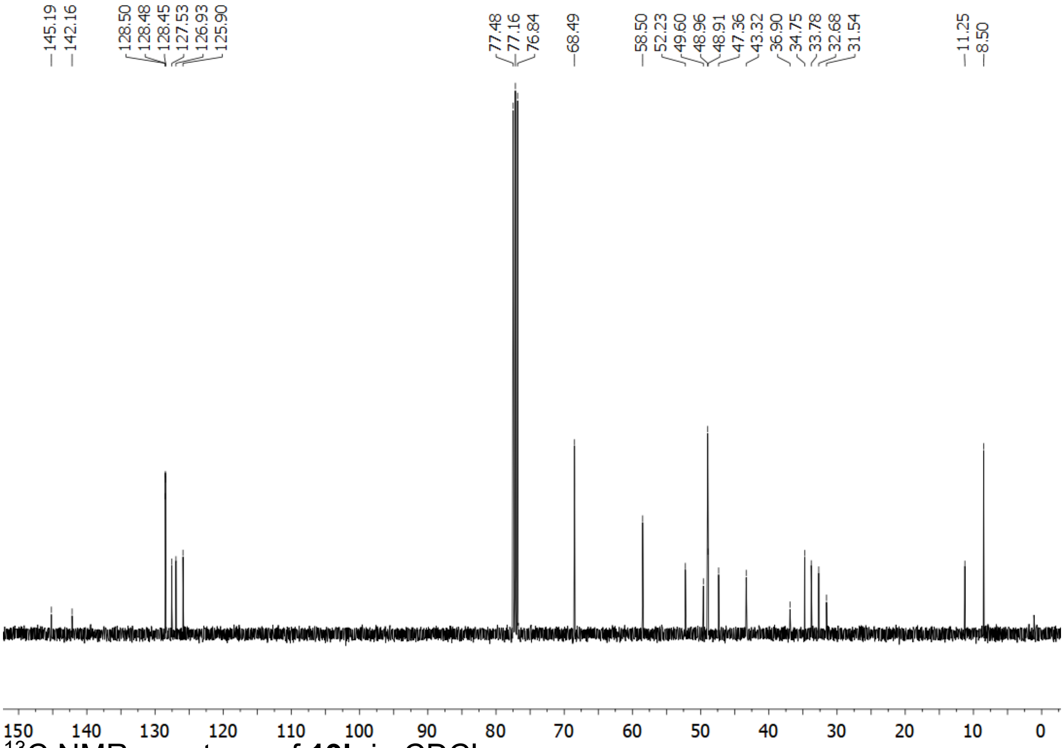

^{13}C NMR spectrum of **19b** in $CDCl_3$.

***cis*- and *trans*-N-(Cyclohexylmethyl)-2-(1-methyl-2-phenylpiperidin-4-yl)ethan-1-amine (*cis*-20a and *trans*-20a)**

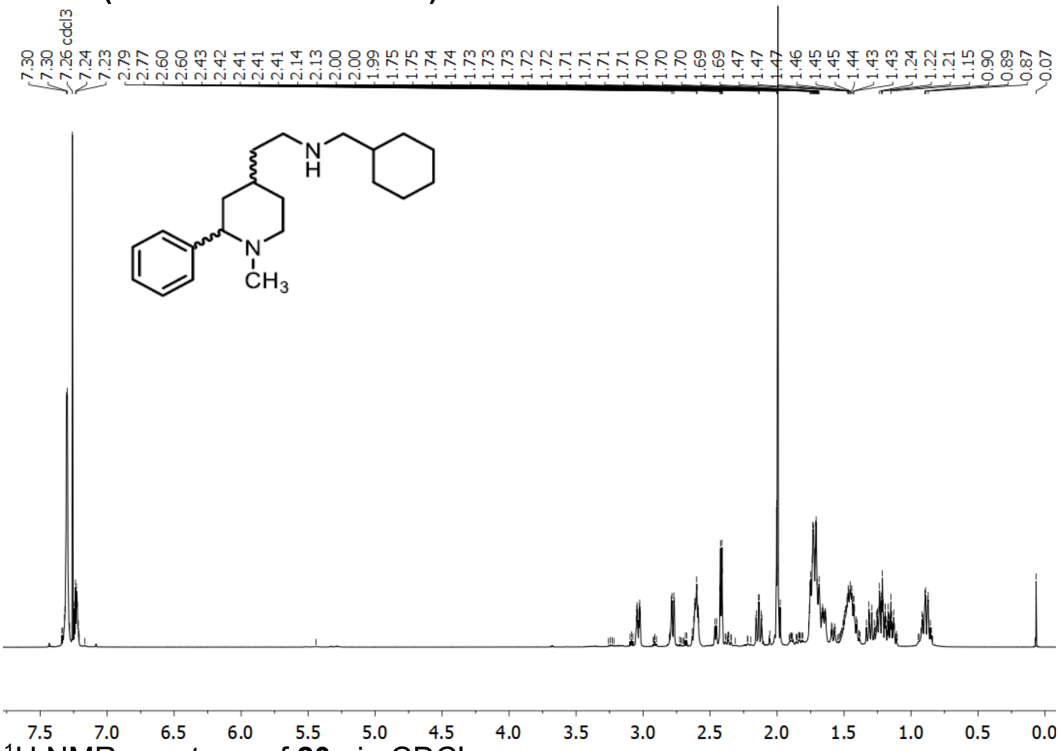

^{1}H NMR spectrum of **20a** in CDCl$_3$.

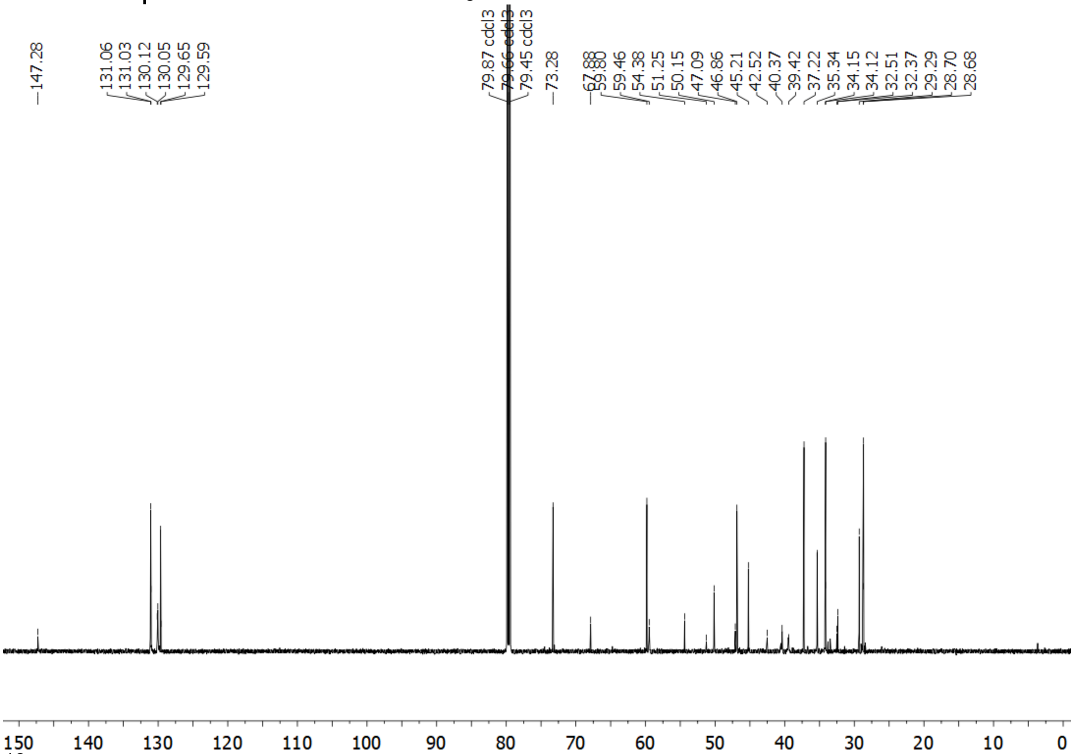

^{13}C NMR spectrum of **20a** in CDCl$_3$.

cis- and *trans*-N-Benzyl-N-methyl-2-(1-methyl-2-phenylpiperidin-4-yl)ethan-1-amine (*cis*-21a and *trans*-21a)

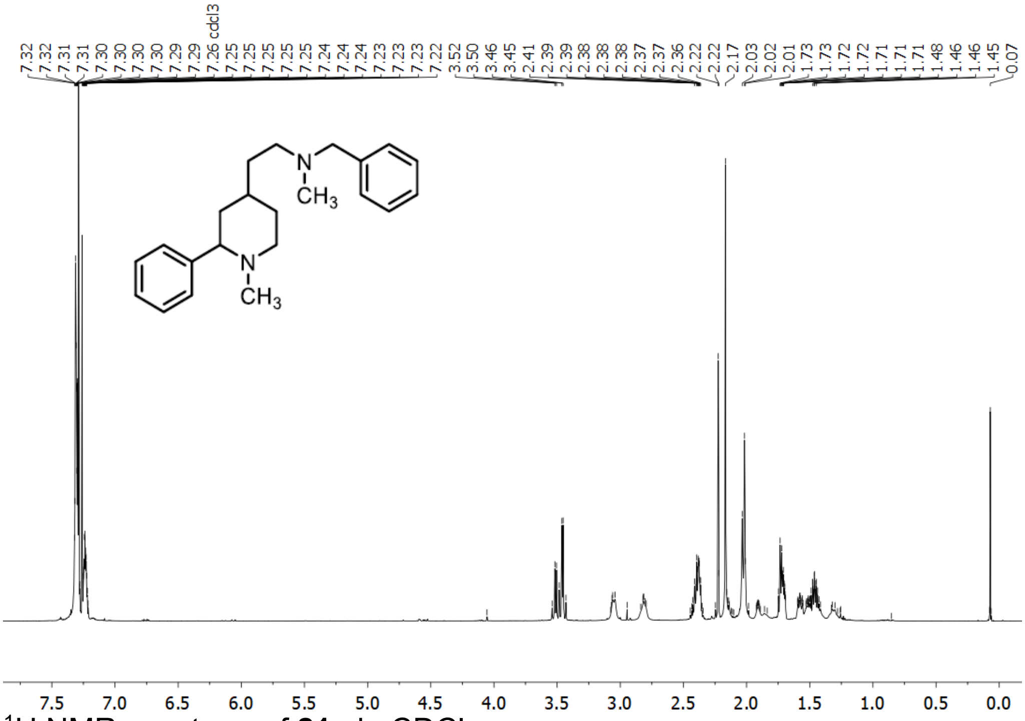

^{1}H NMR spectrum of **21a** in $CDCl_3$.

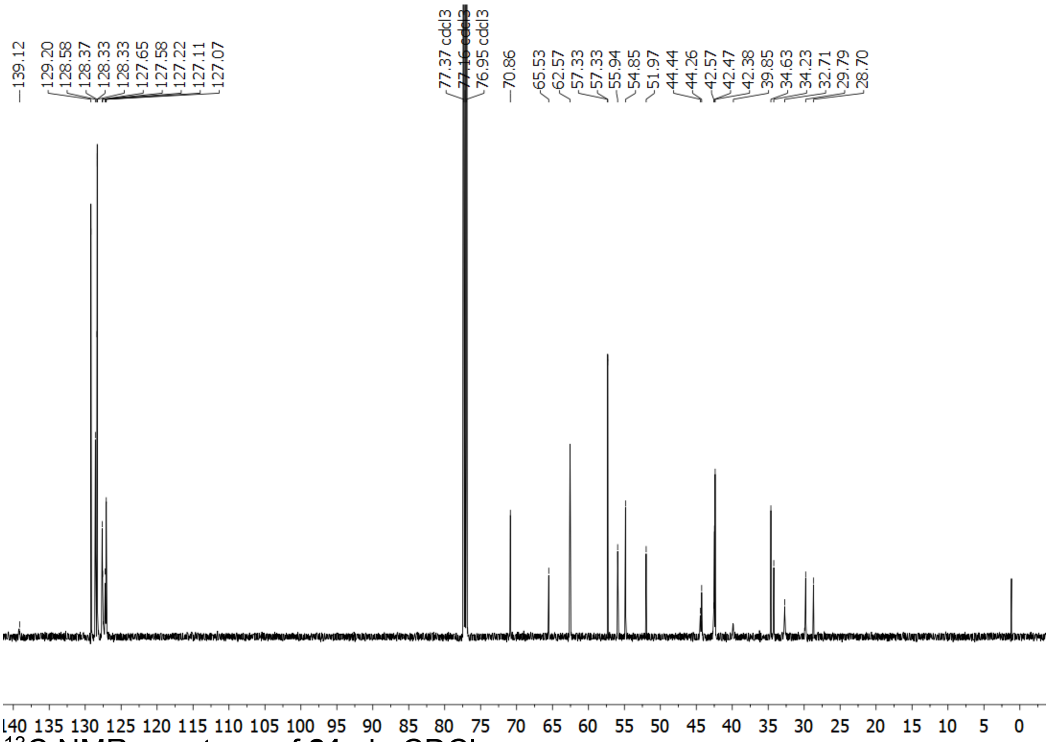

^{13}C NMR spectrum of **21a** in $CDCl_3$.

cis- and _trans_-1-(2-(1-Methyl-2-phenylpiperidin-4-yl)ethyl)-4-phenylpiperazine (_cis_-22a and _trans_-22a)

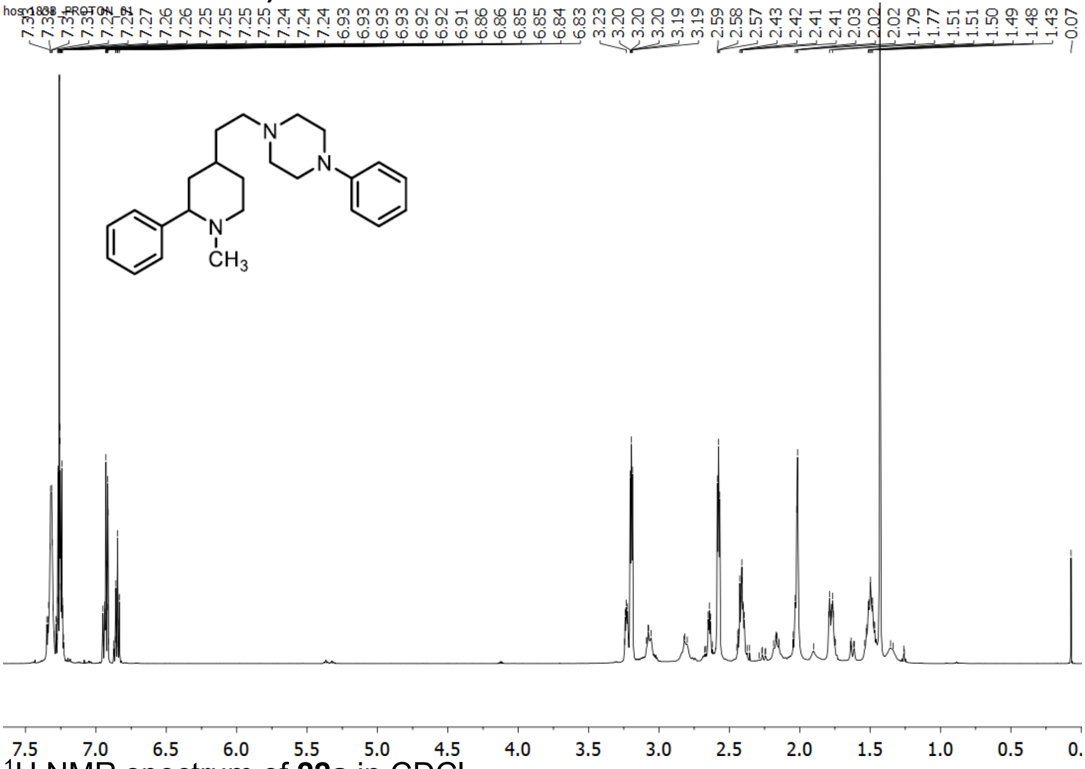

^{1}H NMR spectrum of **22a** in $CDCl_3$.

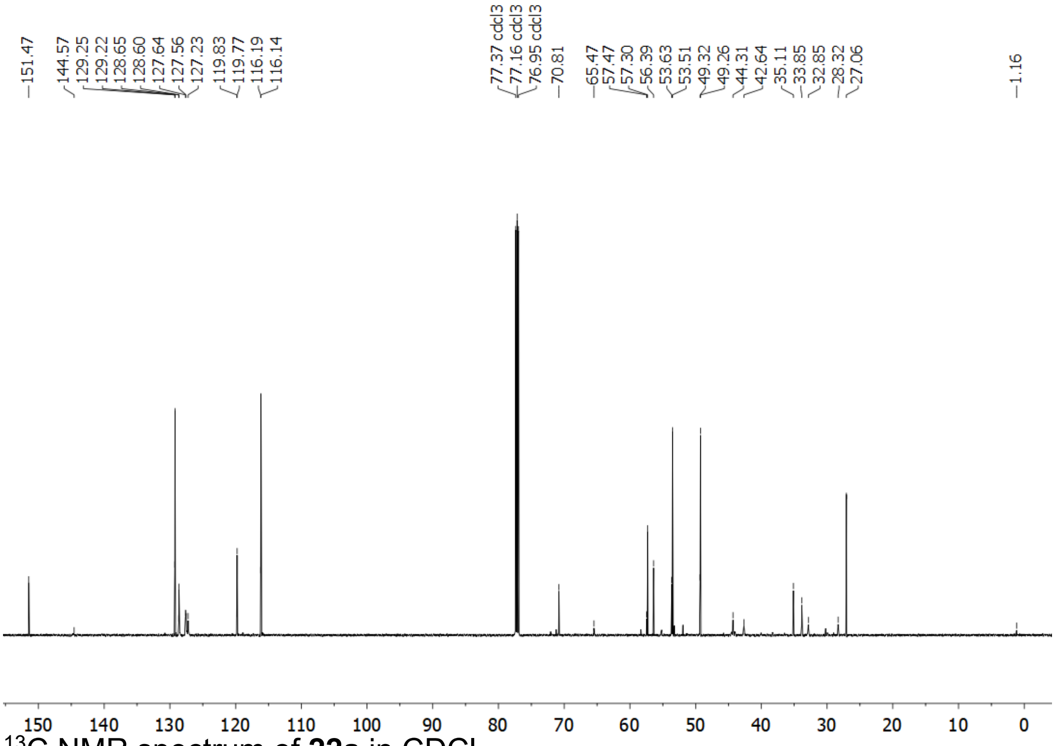

^{13}C NMR spectrum of **22a** in $CDCl_3$.
